# Lactate transmission from hypoxic tumor cells promotes macrophage senescence and M2 polarization via the DNMT1-NHE7 axis to accelerate endometrial cancer progression

**DOI:** 10.1038/s41419-026-08411-y

**Published:** 2026-01-30

**Authors:** Shizhou Yang, Yuejiang Ma, Tingting Wu, Xiufeng Huang

**Affiliations:** 1 Department of Gynecology, Women’s Hospital, Zhejiang University School of Medicine, Hangzhou, Zhejiang China; 2 Zhejiang Key Laboratory of Precision Diagnosis and Therapy for Major Gynecological Diseases, Hangzhou, Zhejiang China

**Keywords:** Cancer models, Cancer metabolism

## Abstract

Although hypoxia is a well-known key driver of metabolic reprogramming in endometrial cancer (EC), its role in lactate-mediated macrophage activation remains unclear. This study investigates whether hypoxia-mediated lactate metabolism reprogramming facilitated EC progression via macrophages. Our data demonstrated that hypoxia-inducible factor 1 subunit alpha (HIF1A) drives a lactate-regulated metabolic cascade, elevating glycolytic genes and monocarboxylate transporter 3 (MCT3) in EC cells to produce and export more lactate. This lactate is transported to macrophages by MCT1 to drive M2 macrophage polarization. Mechanistically, lactate induces lactylation of Histone 3 in the promoter of DNA methyltransferase 1 (DNMT1) gene and activates transcription in macrophages, leading to the silencing of NHE7 gene expression, a key regulator of intracellular pH. Critically, NHE7 downregulation drives M2 polarization and senescence through the mitogen-activated protein kinase (MAPK) pathway activation in macrophages, ultimately facilitating EC progression. In vivo, we successfully established a xenograft tumor model using Ishikawa cells, and the data further confirmed that NHE7-overexpressing macrophages effectively abrogate exogenous lactate-accelerated xenograft tumor growth, as well as its M2 polarization and senescence. These findings uncover that hypoxia-mediated lactate production and transmission promote tumor-macrophage crosstalk via the DNMT1-NHE7 axis and EC progression, which offers novel therapeutic targets for EC.

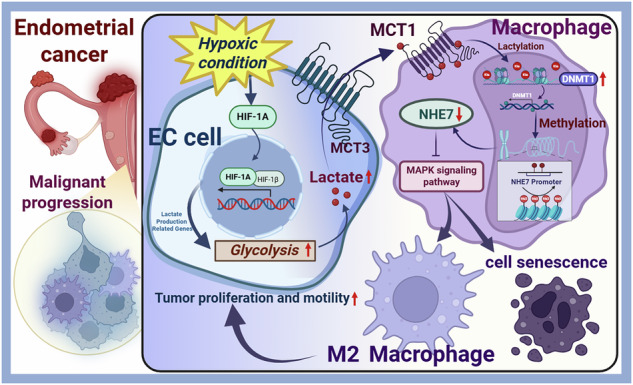

## Introduction

Endometrial cancer (EC), a malignancy arising from endometrial epithelial cells, exhibits a rising global incidence, ranking as the sixth most common female cancer with over 417,000 new cases and 97,000 deaths annually [1, 2]. Current therapeutic strategies, including surgery, radiotherapy, and chemotherapy, remain the cornerstone of EC management. However, tumor recurrence, metastasis, and resistance often limit these approaches, particularly in advanced stages [3, 4]. Emerging evidence highlights the pivotal role of the tumor microenvironment (TME) in driving EC progression and therapeutic evasion [5, 6], positioning it as a critical target for novel interventions. Pan *et al*. reported that the endometrium undergoes dynamic remodeling under cyclic hormonal regulation, characterized by estrogen-driven sterile inflammation and chronic immune activation, which fosters a unique microenvironment enriched with infiltrating lymphocytes and macrophages [7]. This immunogenic landscape suggests ECs’ potential responsiveness to immunotherapy. However, approved immunotherapies are scarce and challenged by resistance [8]. Understanding the EC immune microenvironment and its underlying mechanisms is critical for elucidating tumor progression and developing novel therapies, offering profound clinical and scientific implications.

Hypoxia, a hallmark of the TME, arises from uncontrolled tumor proliferation and aberrant vascularization and has recently emerged as a key target in cancer therapy [9, 10]. It activates hypoxia-inducible factor 1 subunit alpha (HIF1A), which promotes tumor cell survival, invasion, and immunosuppression by reshaping metabolic pathways such as glycolysis [11]. Metabolic reprogramming is a fundamental trait of tumor cells, allowing them to adapt to external stresses such as hypoxia and nutrient deprivation [12]. Additionally, metabolites derived from this reprogramming regulate effector functions of diverse immune cells in the TME, thereby driving tumor malignancy [13]. For instance, accumulated lactate in the TME can induce an immunosuppressive environment by mediating T/NK cell dysfunction and influencing macrophage polarization, contributing to tumor immune evasion [14, 15]. Similarly, methionine metabolic reprogramming drives T-cell exhaustion in hepatocellular carcinoma [16], and elevated 25-hydroxycholesterol levels in the TME mediate an immunosuppressive macrophage phenotype, correlating with poorer survival in pan-cancers [17]. Emerging studies underscore the pivotal role of metabolic dysregulation in EC pathogenesis, where the metabolic syndrome is a recognized risk factor [18], and lipid/glucose metabolic reprogramming critically drives tumor initiation and progression [19, 20].

Tumor-associated macrophages (TAMs), the most abundant immune infiltrates in the TME [15], are polarized into immunosuppressive M2 phenotypes under hypoxic conditions, facilitating angiogenesis, metastasis, and immune evasion [21, 22]. Critically, the interaction between cancer cells and TAMs in the hypoxic TME significantly influences tumorigenesis and could be a promising therapeutic target in cancer [23]. Emerging single-cell RNA sequencing (scRNA-seq) studies highlight hypoxia’s role in orchestrating intercellular communication within the TME, significantly altering ligand-receptor interactions among malignant and stromal cells [24]. Among the key mediators of this crosstalk, hypoxia-inducible chemokines and tumor-derived exosomes have emerged as critical conduits for bidirectional signaling between TAMs and cancer cells [25, 26]. Previous work by our group has established a foundational context for EC research: We systematically evaluated diagnostic methodologies, reinforcing the need for accurate early detection, while our data also identified that the Na^+^/H^+^ exchanger 7 (NHE7) transporter promotes tumor progression via the cAMP/CREB/GRIN2B axis, implicating calcium signaling and delayed senescence as key oncogenic mechanisms [27, 28]. Despite these insights, the mechanistic interplay between EC-specific metabolic alterations and immune remodeling remains poorly characterized. Elucidating these cross-talk mechanisms is critical to uncovering actionable metabolic targets that could disrupt immune tolerance, enhance antitumor immunity, and ultimately improve therapeutic outcomes.

In this study, we investigated the intricate molecular mechanisms linking hypoxia-induced metabolic changes in EC to macrophage polarization. We focused on how reduced oxygen levels trigger a cascade of biochemical reactions, leading to alterations in cellular metabolism in EC. These metabolic shifts were observed to influence the polarization states of macrophages, driving them towards an anti-inflammatory M2 phenotype. The study delved into specific signaling pathways, such as HIF1A activation, glycolytic reprogramming, and mitogen-activated protein kinase 1 (MAPK) pathways. Additionally, we examined the impact of these metabolic adaptations on macrophage function and feedback regulation in EC.

## Results

### HIF1A drives lactate-mediated glycolytic reprogramming and correlates with poor prognosis in EC

**Fig. 1:**
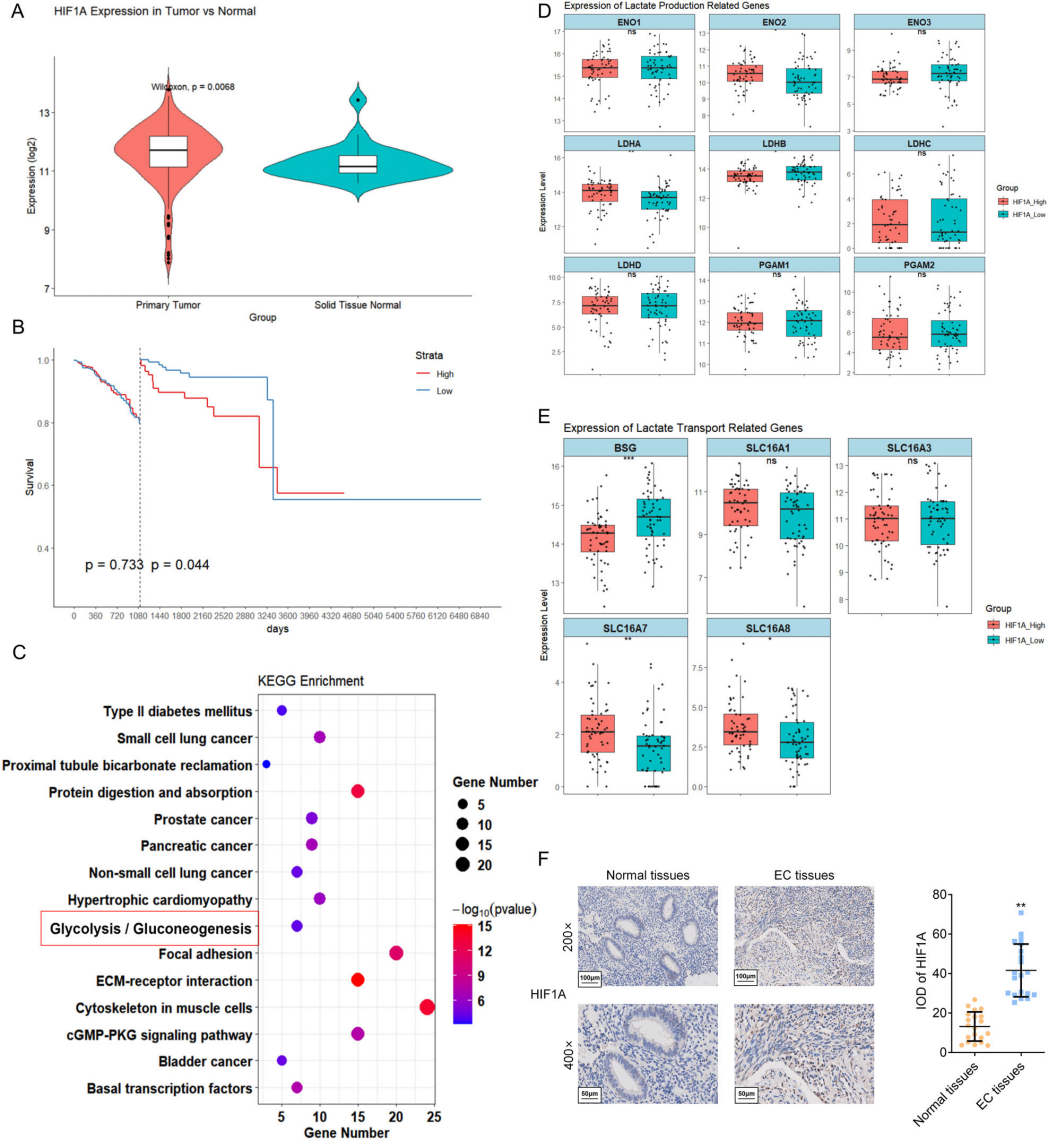
The core role of HIF1A in lactate metabolism of EC. **A** A boxplot visualized the expression of HIF1A in normal tissues and primary EC tissues, using the EC transcriptome data downloaded from the TCGA database. **B** Kaplan-Meier survival analysis was performed to evaluate the association between HIF1A expression levels and patient prognosis. **C** Signal pathways of DEGs enriched by HIF1A in EC were clarified through KEGG enrichment analysis. **D**,**E** Box plots were used to analyze the expression of **D** lactate production-related genes or E lactate transport-related genes in EC cells with HIF1A_High or _Low expression. *P < 0.05, **P < 0.01. **F** The expression of HIF1A in EC tissues and normal tissues was detected by IHC assay. Scale bar: 100 μm (200×), Scale bar: 50 μm (400×); *N* = 20; **P < 0.01.

In The Cancer Genome Atlas (TCGA) EC cohort, 194 tumor samples were stratified into hypoxia_High and _Low groups based on median single-sample gene set enrichment analysis (ssGSEA) scores, revealing a significant difference in pathway enrichment between these groups (Fig. S1A). Stratification of EC by hypoxia score revealed significant transcriptomic alterations. 1200 differentially expressed genes (DEGs) were detected (199 up/1,001 down) between hypoxia_High and _Low groups (Fig. S1B), highlighting hypoxia-responsive genes. Functional enrichment analysis confirmed that these DEGs were significantly involved in hallmark hypoxic processes, including the HIF1A signaling pathway, cAMP signal pathway, and glycolysis (Fig. S1C, D). Crucially, the Least Absolute Shrinkage and Selection Operator (LASSO)-derived hypoxia scoring model assigned the highest weight to HIF1A (Fig. S1E), alongside the high weights of its established targets [29, 30], reinforcing the signature’s link to hypoxic pathways. To explore HIF1A’s role in hypoxic EC progression, we analyzed the TCGA data and revealed that HIF1A was significantly upregulated in primary EC tissues compared with normal controls, as indicated by a boxplot (*p* = 0.0068, Fig. 1A). Additionally, high HIF1A expression correlated with poorer patient prognosis through the Kaplan-Meier survival analysis (*p* = 0.044) (Fig. 1B). Transcriptome analysis identified 785 DEGs between HIF1A_High and _Low groups in EC (Fig. S2A), which were significantly enriched in glycolysis-related pathways, as depicted by Kyoto Encyclopedia of Genes and Genomes (KEGG) (Fig. 1C) and Gene Ontology (GO) (Fig. S2B) analyses. Focusing on glycolysis, we observed notable upregulation of key enzymes, including hexokinase 2 (HK2), lactate dehydrogenase A (LDHA), enolase 2 (ENO2), aldolase, fructose-bisphosphate B (ALDOB), and phosphoglycerate kinase 1 (PGK1) in HIF1A_High tumors (Fig. S2C). Notably, lactate synthesis genes (LDHA and ENO2) and the lactate exporter MCT3 (solute carrier family 16 member 8: SLC16A8), key factors in lactate production and secretion [31] were also elevated (Fig. 1D, E), suggesting glycolysis reprogramming. Furthermore, IHC staining of clinical samples further demonstrated higher HIF1A protein levels in EC tissues (N = 20) than in adjacent normal tissues (Fig. 1F). Collectively, these findings establish HIF1A as the central mediator of hypoxia in EC, where its upregulation, linked to poor patient prognosis, orchestrates a transcriptional program driving glycolytic reprogramming and lactate metabolism.

### Hypoxia promotes EC malignant progression and lactate metabolism through HIF1A in vitro

**Fig. 2:**
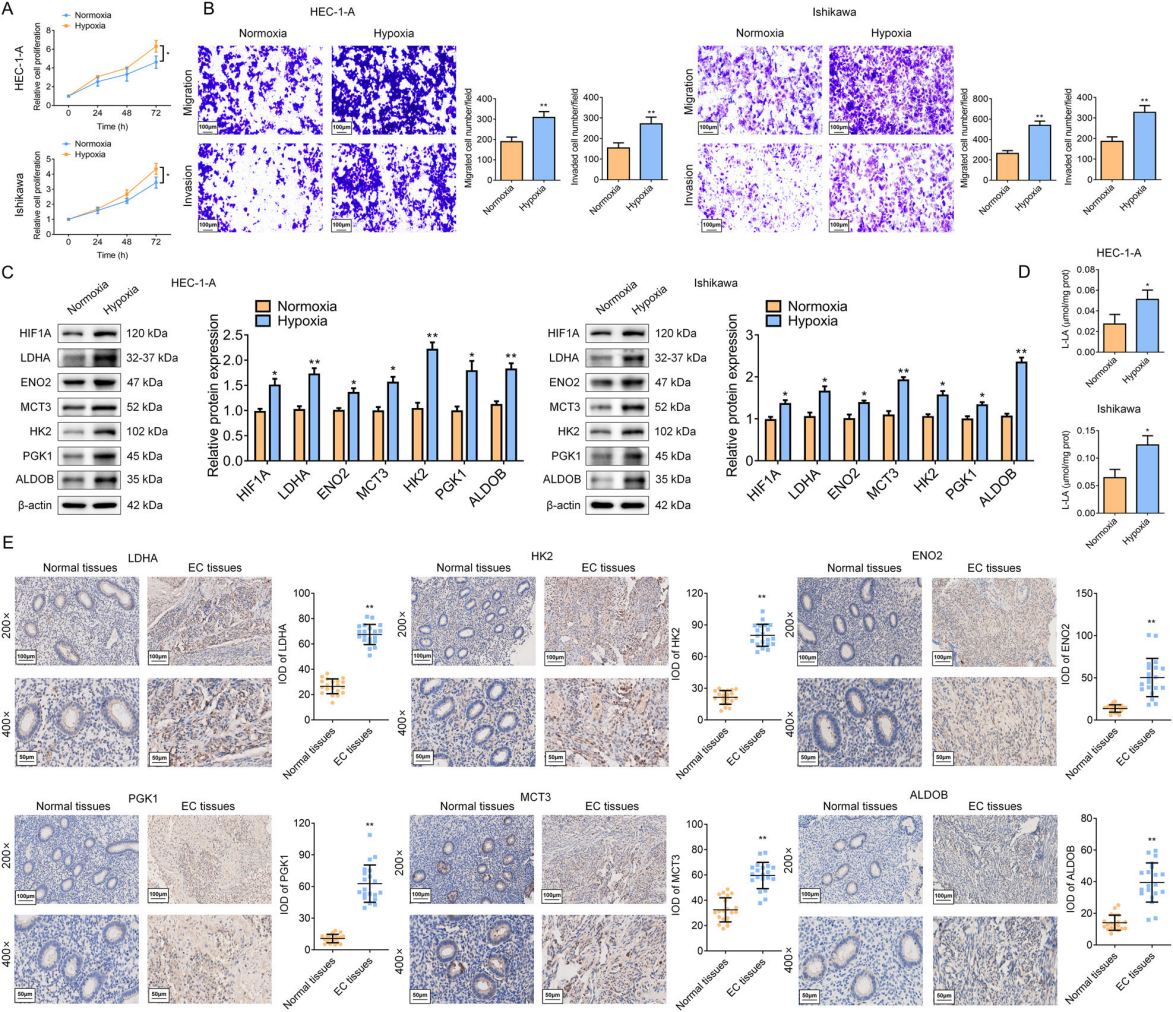
HIF1A promotes lactate-mediated glycolytic reprogramming and aggressiveness in EC. **A**, **B** The effects of normoxic and hypoxic conditions on the proliferation, migration, and invasion abilities of Ishikawa and HEC-1-A cells were examined using the MTT assay (**A**) and Transwell assay (**B**). Scale bar: 100 μm; *N* = 3; *P < 0.05, **P < 0.01. **C** The effect of hypoxia on HIF1A, LDHA, ENO2, MCT3, HK2, PGK1, and ALDOB protein levels was detected by WB. *N* = 3; *P < 0.05, **P < 0.01. **D** The changes in lactate levels in the SN of Ishikawa and HEC-1-A cells under hypoxic or normoxic conditions were detected using a Lactate Assay Kit. *N* = 3; *P < 0.05. **E** The expression of LDHA, ENO2, MCT3, HK2, PGK1, and ALDOB in clinical EC tissues or normal tissues was detected by IHC. Scale bar: 100 μm (200×), Scale bar: 50 μm (400×); N = 20; *P < 0.05, **P < 0.01.

Next, we subjected EC cell lines (HEC-1-A and Ishikawa) to either hypoxic (1% O₂) or normoxic (21% O₂) conditions. In vitro experiments demonstrated that hypoxia significantly enhanced cell proliferation and the capacities of migration and invasion compared to normoxia in both cell lines (Fig. 2A, B). Additionally, WB assays revealed that hypoxia upregulated HIF1A expression, along with lactate synthesis enzymes (LDHA and ENO2), transporter (MCT3), and glycolytic regulators (HK2, PGK1, and ALDOB) expression at protein levels (Fig. 2C). Consistently, lactate concentrations in hypoxic EC cell supernatant (SN) significantly increased compared to normoxia (Fig. 2D). IHC analysis of clinical specimens (*N* = 20) validated the upregulation of LDHA, ENO2, MCT3, HK2, PKM1, and ALDOB in EC tissues compared to normal tissues (Fig. 2E), further corroborating the bioinformatic and In vitro findings. To establish causality, HIF1A was silenced in Ishikawa cells using siRNA (siR-HIF1A-2, selected for optimal knockdown efficiency) (Fig. S3A). The results of the rescue experiment revealed that silencing of HIF1A partially abolished hypoxia-induced proliferation, lactification, and migratory & invasive phenotypes (Fig. S3B–D), and normalized glycolytic protein expression (Fig. S3E), establishing HIF1A as the key mediator of hypoxia-driven oncogenic reprogramming in EC.

### Hypoxia-induced HIF1A-MCT3 axis drives M2 macrophage polarization via lactate shuttling to promote EC progression

**Fig. 3:**
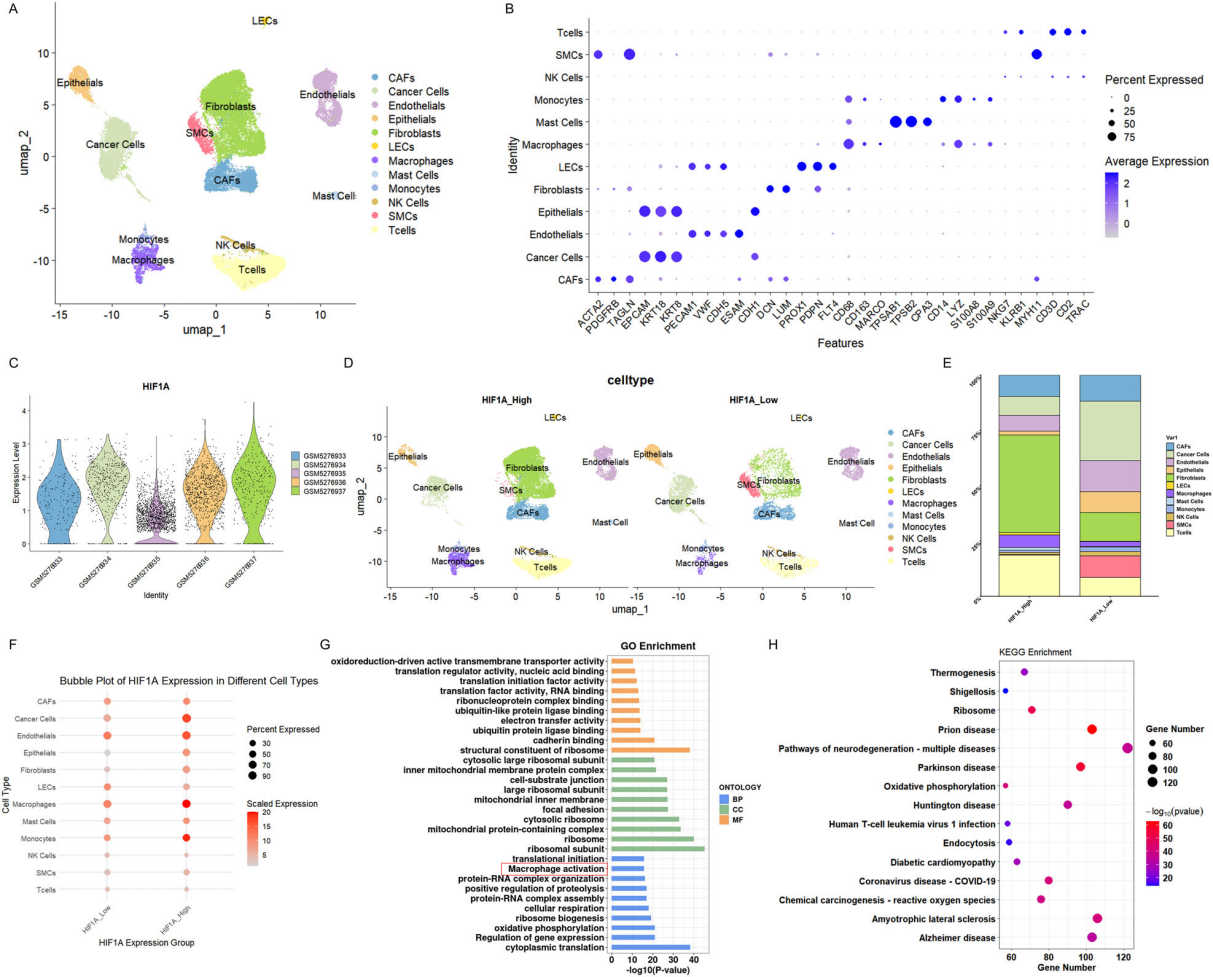
ScRNA-seq analysis reveals HIF1A-mediated regulation of macrophage polarization and gene networks in the EC microenvironment. **A** UMAP visualization & **B** Featureplot of 12 cell clusters identified after integration, quality control, and clustering of five EC single-cell datasets (GSM5276933-GSM5276937 from the GEO database). **C** Violin plot comparing HIF1A expression in cancer cells across samples, categorizing subgroups into HIF1A_High (GSM5276934, GSM5276936, GSM5276937) and HIF1A_Low (GSM5276933, GSM5276935). **D** Subgroup-specific UMAP visualization of HIF1A_High versus HIF1A_Low samples. **E** Bar chart depicting cell-type proportions across HIF1A subgroups. **F** Bubble chart comparing HIF1A expression in different cell types between HIF1A_High and HIF1A_Low subgroups. **G** GO and **H** KEGG enrichment analysis of macrophage-related DEGs in HIF1A-stratified tumor cells in ScRNA-seq data.

Based on the marked upregulation of the lactate efflux transporter MCT3 [32] in hypoxic EC cells, and given the MCT family’s role in lactate shuttle and TAM polarization [33, 34], we explored the EC cell-macrophage link. First, we analyzed five EC single-cell datasets (GSM5276933, GSM5276934, GSM5276935, GSM5276936, and GSM5276937) from the Gene Expression Omnibus (GEO) database. Following quality control, dimensionality reduction via PCA, Harmony integration, Louvain clustering, and Uniform Manifold Approximation and Projection (UMAP) visualization, we identified 12 distinct cell clusters (Fig. 3A, B), manually annotated as epithelial, lymphatic endothelial cells (LECs), immune (T/NK cells, monocytes, and macrophages), stromal (fibroblasts and endothelial cells), cancer-associated fibroblasts (CAFs), smooth-muscle-cell (SMCs), mast cells, and cancer cells. HIF1A expression in cancer cells was compared across samples, revealing lower expression in GSM5276933 and GSM5276935, visualized by a violin plot (Fig. 3C). Samples were thus classified into HIF1A_High (GSM5276934, GSM5276936, and GSM5276937) and HIF1A_Low (GSM5276933 and GSM5276935) subgroups. Inter-group-specific UMAP plots (Fig. 3D), bar chart of inter-group cell proportions (Fig. 3E), and bubble chart of inter-group expression of HIF1A (Fig. 3F) demonstrated significant differences in HIF1A expression between groups for macrophages. Supporting this observation, GO/KEGG enrichment analysis of macrophage-related DEGs in HIF1A-stratified tumor cells highlighted macrophage polarization as a significantly enriched pathway (Fig. 3G, H), highlighting the intricate interplay among HIF1A, macrophages, and the broader gene regulatory network within the EC microenvironment.

**Fig. 4:**
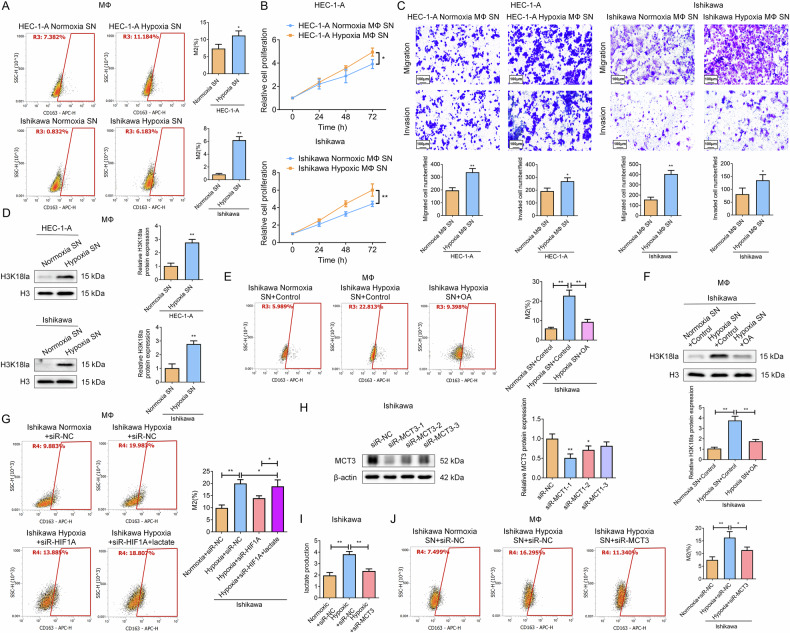
Hypoxic EC cell-derived lactate drives M2 macrophage polarization to promote EC progression via HIF1A and MCT3. **A** Flow cytometry analysis of CD163 in macrophages treated with hypoxic HEC-1-A/Ishikawa cell SN. N = 3; *P < 0.05, **P < 0.01. **B**, **C** Proliferation (**B**) and migration/invasion (**C**) of naive HEC-1-A/Ishikawa cells exposed to M2 macrophage SN derived from normoxic/hypoxic EC-treated macrophages. Scale bar: 100 μm; *N* = 3; *P < 0.05, **P < 0.01. (D-F) Macrophages were treated with SN from normoxic or hypoxic EC cells, with or without OA (10 mM) co-treatment. **D** H3K18la levels were analyzed by WB. **E** M2 polarization was assessed by CD163 expression using flow cytometry. **F** H3K18la expression was examined by WB under OA co-treatment conditions. *N* = 3; **P < 0.01. **G** Flow cytometry analysis of CD163 expression in macrophages treated with SN from hypoxic Ishikawa cells transfected with siR-NC, siR-HIF1A, or siR-HIF1A with lactate supplementation (10 mM). *N* = 3; *P < 0.05, **P < 0.01. **H–J** Ishikawa cells were transfected with either siR-NC or siR-MCT3 plasmids (siR-MCT3-1, selected for optimal knockdown efficiency), followed by treatment under normoxic or hypoxic conditions. Subsequently, the supernatants from these treated Ishikawa cells were used to treat macrophages. **H** MCT3 knockdown efficiency assessed in Ishikawa cells by WB assay. **I** Lactate levels in Ishikawa cell culture supernatant. **J** Macrophage polarization analyzed by CD163 expression using flow cytometry. *N* = 3; *P < 0.05, **P < 0.01.

Subsequently, flow cytometry revealed a significant increase in CD163 (M2 macrophage marker) expression in macrophages treated with hypoxic EC cell SN versus normoxic, validating that hypoxic tumor cells promote M2 macrophage polarization (Fig. 4A). Importantly, conditioned medium from macrophages treated with hypoxic EC cell SN significantly promoted naive EC cell proliferation, and migration&invasion compared to the control treatment (Fig. 4B, C). WB analysis further illustrated an elevation in histone H3 lactylation (H3K18la), a post-translational modification associated with gene expression regulation and glycolytic metabolism [35], in macrophages exposed to hypoxic EC cell SN (Fig. 4D). Co-treatment with the lactate synthesis inhibitor oxamic acid (OA) significantly reversed the promotion effects of hypoxic Ishikawa cell SN on M2 macrophage polarization and histone H3 lactylation (Fig. 4E, F). Critically, our data further demonstrated that HIF1A silenced under hypoxia suppressed M2 macrophage polarization, an effect rescued by lactate supplementation, complementing prior OA experiments (Fig. 4G). Together, these confirm that hypoxia-induced HIF1A-driven lactate acts as a key mediator promoting M2 polarization in the TME. Furthermore, we confirmed MCT3 as the critical lactate transporter in this axis, as its silencing trapped lactate intracellularly and effectively abrogated M2 polarization (Fig. 4H–J), collectively establishing that MCT3-mediated lactate shuttling under hypoxia-induced HIF1A drives M2 macrophage polarization, thereby promoting EC progression.

### RNA-seq reveals differential gene expression and pathway enrichment in hypoxia-induced macrophage polarization, with DNMT1 and NHE7 implicated in EC progression via single-cell and TCGA analyses

**Fig. 5:**
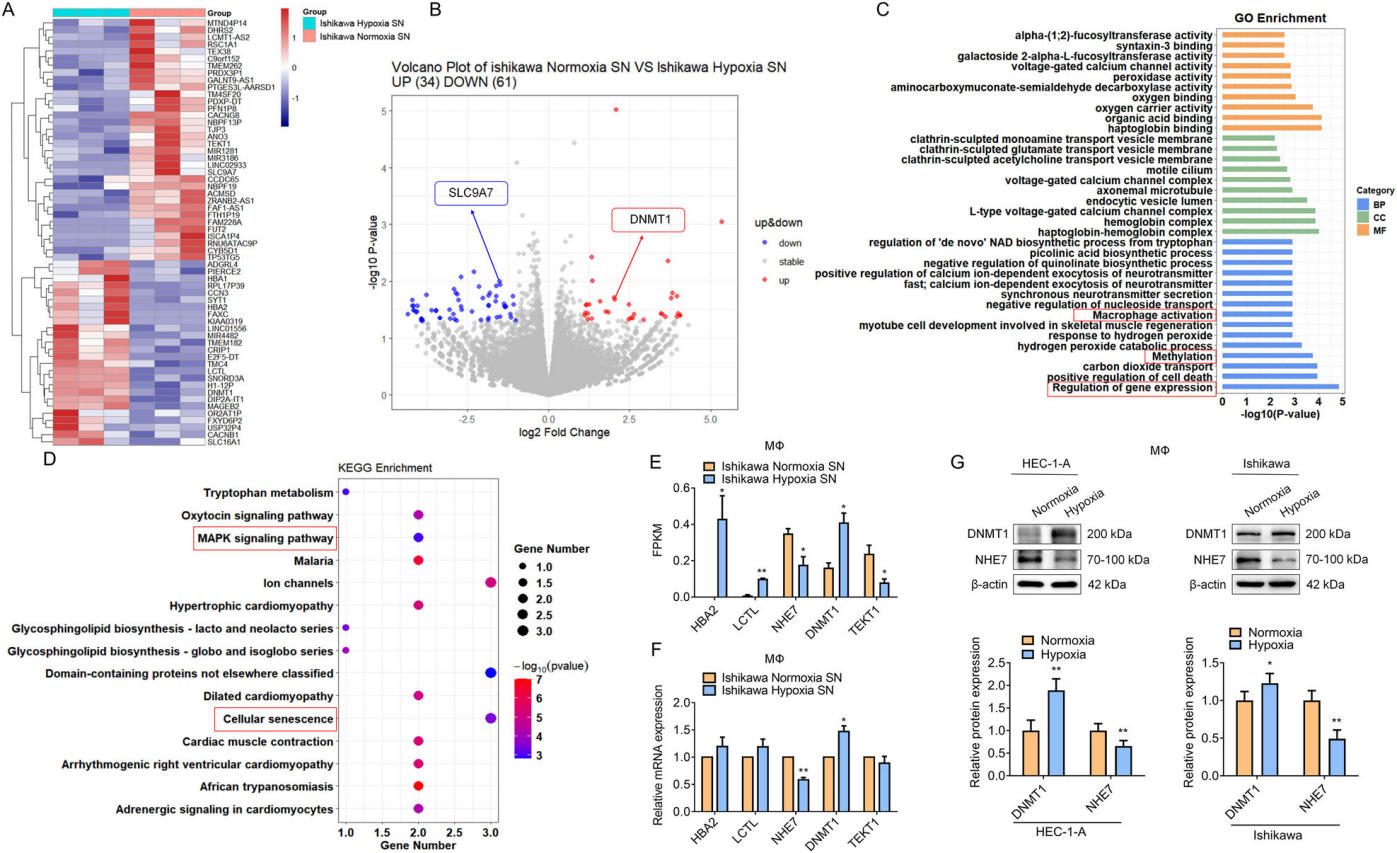
Transcriptomic and functional profiling of macrophages exposed to hypoxic EC microenvironment signals. **A**, **B** RNA-seq was performed on macrophages treated with hypoxic Ishikawa cell SN, and the related DEGs were presented by a heatmap (**A**) and a volcano plot (**B**), with normoxic conditions as control. **C**, **D**The functional roles of the DEGs were analyzed through GO (**C**) and KEGG (D) pathway enrichment analyses. **E** The expression levels of HBA2, LCTL, NHE7, DNMT1, and TEKT1 genes in macrophages induced by hypoxic EC were detected by FPKM. *N* = 3; *P < 0.05, **P < 0.01. **F** The expression levels of HBA2, LCTL, NHE7, DNMT1, and TEKT1 genes in macrophages induced by hypoxic EC were detected by RT-qPCR. N = 3; *P < 0.05, **P < 0.01. **G** The expression of DNMT1 and NHE7 proteins in macrophages induced by hypoxic EC SN was detected by WB. N = 3; *P < 0.05, **P < 0.01.

We next performed RNA-seq on macrophages exposed to hypoxic Ishikawa cell SN (with normoxia as control) to profile DEGs underlying the polarization mechanism. A heatmap depicted distinct gene expression profiles(Fig. 5A), and the distribution of these 95 DEGs was visualized using a volcano plot (Fig. 5B), with 34 upregulated and 61 downregulated genes. Notably, genes such as solute carrier family 9 member A7 (SLC9A7), also known as NHE7, hemoglobin subunit alpha 2 (HBA2), lactase-like (LCTL), DNA methyltransferase 1 (DNMT1), and tektin 1 (TEKT1) were among the top DEGs, suggesting their potential involvement in macrophage polarization and gene expression regulation. GO and KEGG pathway enrichment analyses further elucidated the functional roles of these DEGs, revealing enrichment in pathways such as gene expression regulation, macrophage polarization, cellular senescence, the MAPK pathway, and methylation (Fig. 5C, D). Fragments Per Kilobase of transcript per Million mapped reads (FPKM), Real-time quantitative polymerase chain reaction (RT-qPCR), and western blot (WB) analyses collectively confirmed DNMT1 upregulation and NHE7 downregulation in hypoxic EC-induced macrophages (Fig. 5E–G).

Further exploration, integrated analysis of scRNA-seq samples from EC (GSE173682; N = 11) revealed distinct cellular clusters via UMAP visualization (Fig. S4A). Re-clustering of M2 macrophages identified nine subpopulations. Strikingly, DNMT1 and NHE7 exhibited a mutually exclusive expression pattern, being highly enriched in separate subpopulations (7 and 8, respectively), confirming their negative correlation (Fig. S4B, C). In TCGA EC tumor samples, HIF1A showed significant positive correlations with NHE7 and DNMT1, and NHE7 exhibited significant positive correlations with DNMT1 and M2 macrophage marker CD163 (Fig. S5A). Notably, due to the nature of bulk transcriptomic profiling, these correlations represent global tumor-level associations rather than cell type-specific interactions. Critically, multivariate Cox regression analysis, with the expression of HIF1A, NHE7, DNMT1, and CD163 as independent variables and overall survival (OS) or Disease-Free Survival (DFS) as the dependent variable, revealed that NHE7 expression significantly impacted prognosis (HR = 1.84; Fig. S5B). Furthermore, ssGSEA demonstrated a positive correlation between the expression of DNMT1 and HIF1A and M2 macrophage infiltration, while a significant negative correlation was observed between NHE7 expression levels and M2 macrophage infiltration in the EC microenvironment (Figure S5C). Corroborating these findings, multiplex immunofluorescence (mIF) staining for NHE7, DNMT1, and the M2 macrophage marker CD206 in both EC and normal adjacent tissues provided consistent validation, which furthermore demonstrated a positive correlation between the expression of DNMT1 and and M2 macrophage infiltration, and a significant negative correlation between NHE7 expression levels and M2 macrophage infiltration in the EC microenvironment (Fig. S6). In vivo, we established a subcutaneous xenograft model using Ishikawa cells overexpressing HIF1A (Fig. S7A). The results demonstrated that HIF1A overexpression significantly promoted tumor growth, as evidenced by the macroscopic appearance of dissected tumors (Fig. S7B), as well as by tracking tumor volume (Fig. S7C) and measuring tumor weight (Fig. S7D). Furthermore, lactate levels were markedly increased in the HIF1A-overexpressing tumor tissues (Fig. S7E). Importantly, Chromatin Immunoprecipitation (ChIP) assays confirmed a significant enrichment of histone lactylation on the DNMT1 promoter (Fig. S7F), alongside a substantial increase in the methylation level of the NHE7 promoter (Fig. S7G) in these tumors. These In vivo findings collectively demonstrate that HIF1A overexpression drives tumor progression, elevates intratumoral lactate, and directly orchestrates the epigenetic silencing of NHE7 via a lactate-DNMT1 axis.

### MCT1-mediated lactate metabolism and NHE7-regulated gene expression drive hypoxic EC-induced macrophage polarization: insights from RNA- and scRNA-seq analyses

**Fig. 6:**
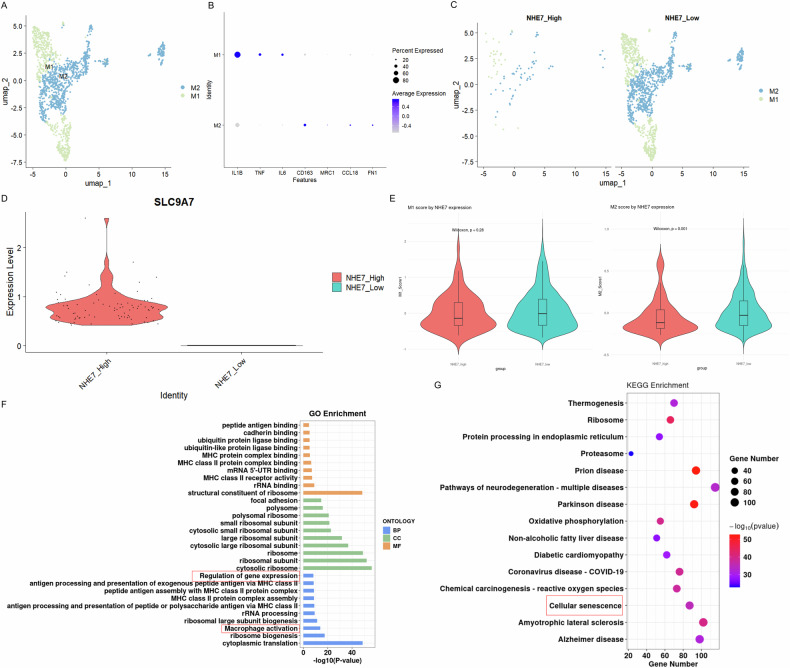
NHE7-stratified macrophage subpopulations and their functional heterogeneity in the EC microenvironment. **A**, **B** Re-clustering and manual annotation of macrophages from five EC single-cell datasets into M1 and M2 subpopulations, visualized by UMAP (**A**) and Featureplot (**B**). **C** UMAP stratification of macrophages into NHE7_High and NHE7_Low subgroups based on median NHE7 expression. **D** Violin plot showing the NHE7 expression in NHE7_High macrophages or NHE7_Low macrophages. **E** Boxplots comparing M1 and M2 macrophage marker scores between NHE7_High and NHE7_Low subgroups. **F**, **G** GO/KEGG enrichment analysis of DEGs between NHE7 subgroups.

For further exploration, macrophages from five EC single-cell datasets were re-clustered and manually annotated into M1 and M2 subpopulations (UMAP visualization; Fig. 6A, B). Macrophages were stratified into NHE7_High and NHE7_Low subgroups using median NHE7 expression. UMAP confirmed subgroup clustering (Fig. 6C), while violin plot validated significantly higher NHE7 expression in NHE7_High macrophages (Fig. 6D). Notably, boxplots comparing M1 and M2 macrophage marker scores between NHE7_high and _low expression groups revealed significantly higher M2 gene scores in the NHE7_low group (*p* = 0.001; Fig. 6E). Differential gene analysis between subgroups highlighted enrichment in pathways regulating gene expression, macrophage polarization, cellular senescence, and related processes (Fig. 6F, G).

**Fig. 7:**
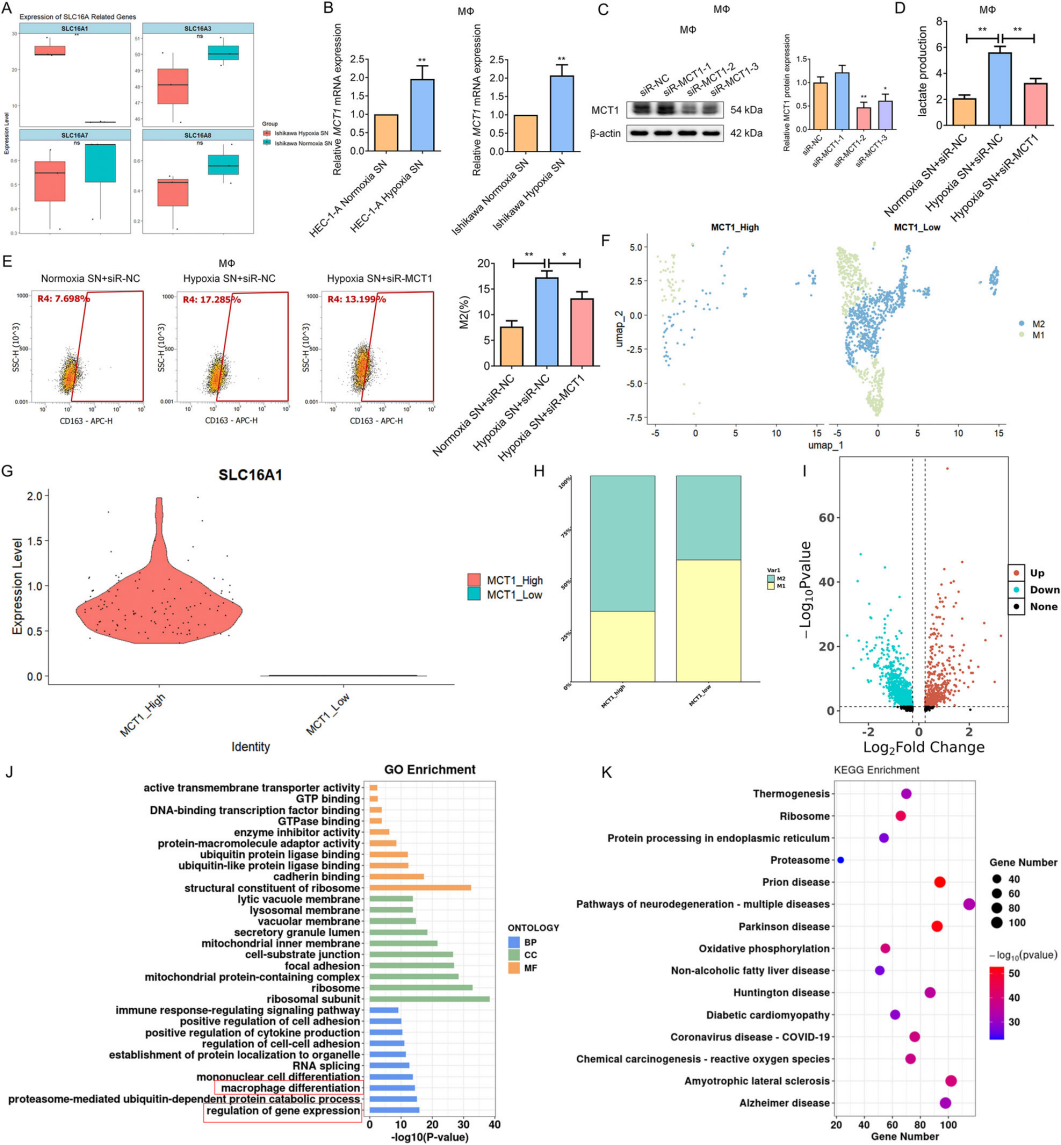
MCT1 upregulation by hypoxic EC cell-derived lactate mediates macrophage heterogeneity. **A** A boxplot analysis of the SLC16A-related gene expression in macrophages exposed to the SN of hypoxic EC cells in RNA-seq. **B** The mRNA expression level of the *SLC16A1* gene in macrophages treated with hypoxic EC cell SN was detected by RT-qPCR. *N* = 3; **P < 0.01. **C** WB analysis confirming MCT1 knockdown efficiency in macrophages (siR-MCT1-2 selected for optimal knockdown efficiency). *N* = 3; **P < 0.01. **D** Lactate levels were measured in the culture medium of control and MCT1-silenced macrophages treated with hypoxic EC cell SN to assess metabolism. *N* = 3; **P < 0.01. **E** Flow cytometry analysis of M2 macrophage polarization (CD163^+^ cells) under the culture medium of control and MCT1-silenced macrophages treated with hypoxic EC cell SN. N = 3; *P < 0.05, **P < 0.01. **F** UMAP visualization of macrophage stratification into MCT1_high and MCT1_low subgroups. **G** Violin plot showing the MCT1 expression in the MCT1 subgroups. **H** Bar chart quantifying the proportion of M1 and M2 macrophages within MCT1_High and MCT1_Low subgroups. I The MCT1-related macrophages in scRNA-seq data were analyzed, and the DEGs were presented by a volcano plot. **J**, **K** The functional roles of the MCT1-related DEGs were analyzed through GO (**J**) and KEGG (**K**) pathway enrichment analyses.

Subsequently, a boxplot analysis of lactate transporters (SLC16A family) expression from RNA-seq data revealed a significant upregulation of SLC16A1 (MCT1) in macrophages exposed to hypoxic EC cell SN (Fig. 7A), a finding validated by RT-qPCR in macrophages treated with SN from hypoxic EC cells (Fig. 7B). To establish its functional role, we silenced MCT1 in macrophages (Fig. 7C). This knockdown significantly rescued the hypoxic EC cell-driven phenotype, reducing both extracellular lactate accumulation (Fig. 7D) and subsequent M2 polarization (Fig. 7E), thereby establishing that MCT1 play a crucial role in facilitating hypoxic EC cell-derived lactate uptake by macrophages. Building on macrophage re-clustering (Fig. 6A, B), we stratified cells by MCT1 expression into MCT1_high and MCT1_low subgroups to further dissect MCT1-associated macrophage heterogeneity. UMAP visualization confirmed distinct subgroup clustering (Fig. 7F), while significantly elevated MCT1 expression in MCT1_high macrophages was validated by a violin plot (Fig. 7G). Crucially, quantification of M1/M2 proportions revealed a significantly higher percentage of M2 macrophages within the MCT1_High subgroup compared to MCT1_Low (Fig. 7H), supporting MCT1’s role in regulating the M1-M2 balance. Additionally, MCT1-related DEGs in subgroups were visualized in the volcano plot (Fig. 7I). GO and KEGG analyses revealed significant enrichment of MCT1-related DEGs involved in macrophage polarization and gene regulation (Fig. 7J, K). In conclusion, our data establish that MCT1-mediated lactate metabolism and NHE7-regulated gene expression drive hypoxic EC-induced macrophage polarization, elucidating a key mechanism of EC cell-macrophage crosstalk.

### NHE7 regulates macrophage polarization and senescence via MAPK signaling to influence EC malignancy

**Fig. 8:**
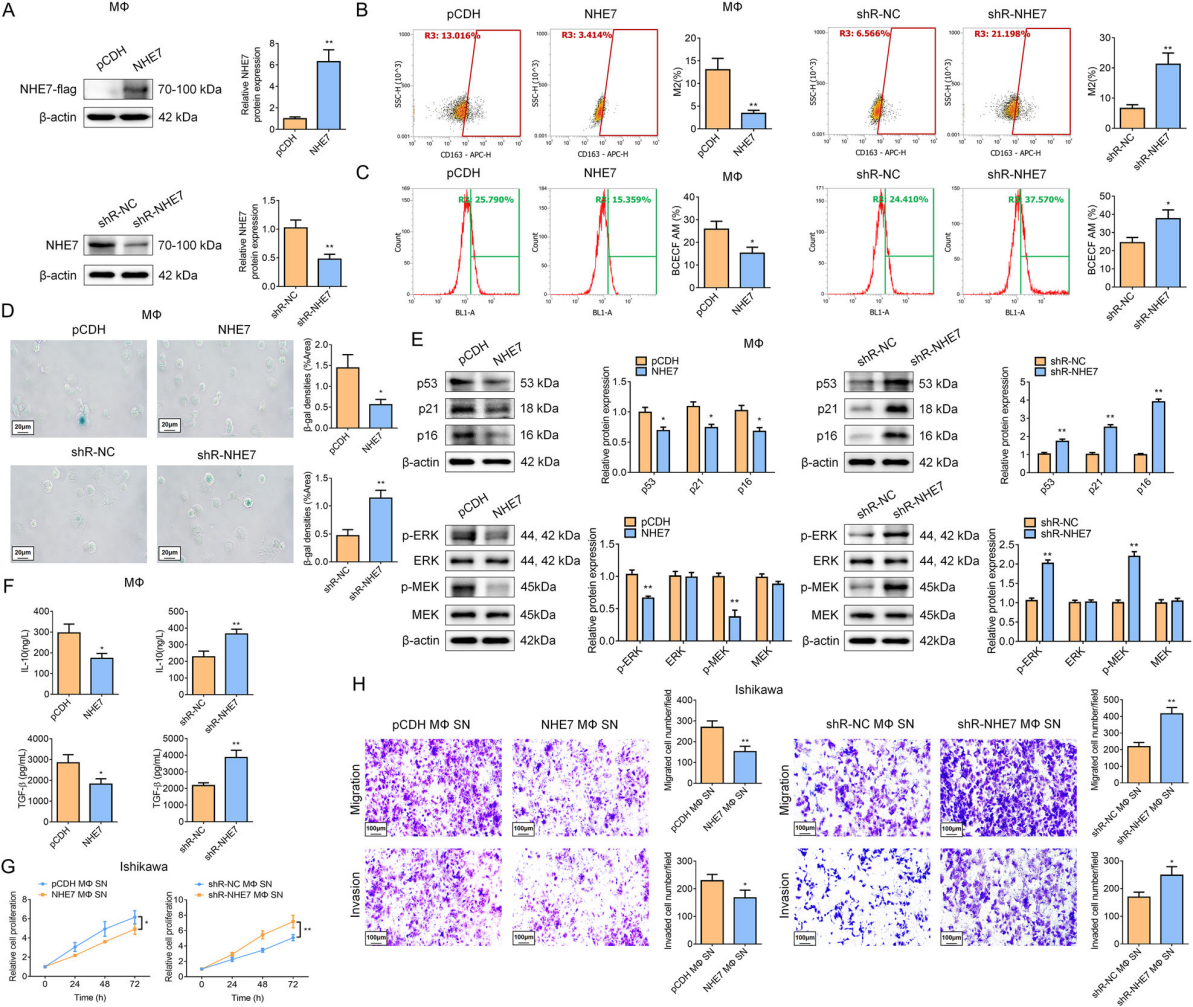
NHE7 regulates macrophage polarization and senescence via the MAPK signaling to influence EC malignancy. **A** The transfection efficiency of overexpression (NHE7) or silencing (shR-NHE7) in macrophages was detected by WB. *N* = 3; **P < 0.01. **B** Flow cytometry was used to detect the proportion changes of M2 macrophages with NHE7 overexpression or shR-NHE7. *N* = 3; **P < 0.01. **C** The pH-sensitive fluorescent probe BCECF AM was used to detect intracellular pH changes in macrophages with NHE7 overexpression or shR-NHE7. N = 3; *P < 0.05. **D** Detection of the effect of NHE7 on macrophage senescence by β-galactosidase staining. Scale bar: 20 μm; *N* = 3; *P < 0.05, **P < 0.01. **E** The impact of NHE7 on the expression of senescence-related markers (P53, P21, and P16) and MAPK-related markers (p-ERK, ERK, p-MEK, and MEK) was detected by WB. N = 3; *P < 0.05, **P < 0.01. **F** The effect of NHE7 on IL-10 and TGF-β in macrophage SN was detected by ELISA. N = 3; *P < 0.05, **P < 0.01. **G**, **H** The effects of SN from NHE7-overexpressing or NHE7-silenced macrophages on the proliferation (**G**), migration, and invasion (**H**) of EC cells. Scale bar: 100 μm; *N* = 3; *P < 0.05, **P < 0.01.

Building on our previous clinical observation that NHE7 expression correlates with patient age and disease stage in EC [28] (Table S1), we focused on its role as a known lysosomal Na^+^/H^+^ exchanger that critically regulates intracellular pH, cellular metabolism, and ultimately tumor maintenance [36]. First, we manipulated NHE7 expression in macrophages via overexpression (NHE7) or silencing (shR-NHE7), confirming transfection efficiency by WB assay (Fig. 8A). Subsequently, we found that NHE7 overexpression significantly reduced M2-polarized macrophages (CD163 cells decreased by 2.8-fold versus control), whereas NHE7 silenced markedly elevated M2 polarization (CD163 cells increased by 3.2-fold) (Fig. 8B), validating that NHE7 negatively regulates M2 macrophage differentiation. Using the pH-sensitive fluorescent probe BCECF AM, we observed that NHE7 overexpression significantly reduced intracellular pH (indicating a decrease in the macrophage proportion). Conversely, NHE7 silencing elevated intracellular pH (implying an increase in the macrophage proportion) (Fig. 8C). RNA-seq revealed senescence/MAPK pathway enrichment, prompting analysis of NHE7’s effects on senescence and MAPK phosphorylation in macrophages. Surprisingly, our data demonstrated that NHE7 overexpression reduced senescent macrophages, as evidenced by a decrease in β-galactosidase-positive staining, whereas NHE7 silencing had the opposite effect, increasing the number of senescent macrophages (Fig. 8D). Additionally, NHE7 overexpression reduced protein levels of senescence markers (p53, p21, and p16) and phosphorylation of Extracellular Regulated protein Kinase (ERK) and Mitogen-activated protein kinase kinase (MEK) (p-ERK and p-MEK), while silenced NHE7 elevated their expression (Fig. 8E). ELISA detection further revealed that NHE7 overexpression reduced Interleukin 10 (IL-10) and Transforming Growth Factor Beta 1 (TGF-β) secretion, both of them related to SASP [37], whereas NHE7 silencing increased their levels (Fig. 8F). These results suggest that NHE7 regulates the polarization, senescence, and the MAPK signaling in macrophages, potentially influencing their anti-inflammatory functions on EC cells. Next, SN from macrophages with altered NHE7 expression were applied to Ishikawa tumor cells to evaluate effects on cell proliferation, migration, and invasion. Expectedly, NHE7-overexpressing macrophage SN inhibited EC cell proliferation, migration, and invasion, while NHE7-silenced ones promoted these processes (Fig. 8G, H), suggesting NHE7-expressing macrophages have negative regulatory effects via secreted factors on tumor aggressiveness. To mechanistically dissect the NHE7-MAPK interplay, we employed both loss-of-function and gain-of-function approaches. First, MAPK inhibition (U0126) in NHE7-silenced macrophages significantly rescued the driven polarization, intracellular pH dysregulation, senescence, and MAPK phosphorylation (Fig. S8A–D). Additionally, U0126 partially mitigated the promotion effect of SN from NHE7-silencing macrophages on EC cell proliferation, migration, and invasion (Fig. S8E&F). Crucially, complementing this, NHE7 overexpression combined with MAPK activation (C16-PAF) inversely induced the phenotypic changes observed upon NHE7 silencing (Fig. S9), confirming that MAPK signaling is both necessary and sufficient for NHE7-mediated effects. These data conclusively establish MAPK signaling as a key downstream pathway through which NHE7 regulates macrophage function and tumor-promoting crosstalk.

### Lactate induces DNMT1 upregulation via H3K18la to epigenetically silence NHE7, driving macrophage dysfunction and tumor promotion

**Fig. 9:**
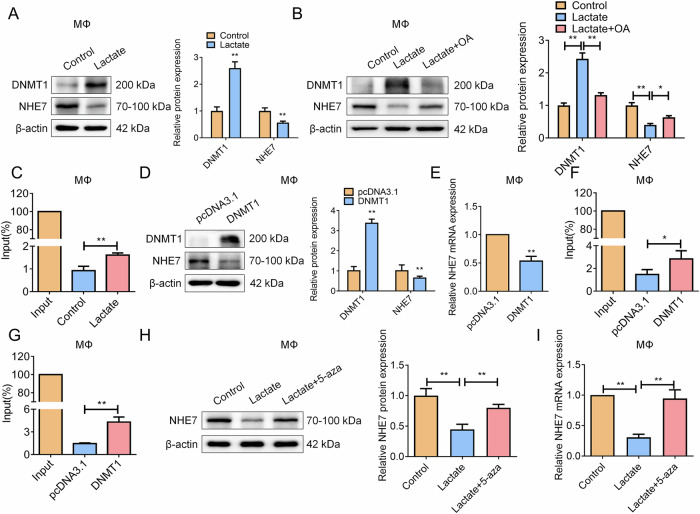
Lactate regulates macrophage polarization and senescence via lactylated DNMT1-mediated methylation inhibition of NHE7. **A** WB was used to detect the protein expression levels of DNMT1 and NHE7 in macrophages treated with the SN of EC cells exposed to hypoxia. *N* = 3; *P < 0.05, **P < 0.01. **B** WB was used to detect the effect of exogenous lactate on the protein expression of DNMT1 or NHE7 in macrophages. *N* = 3; **P < 0.01. **C** ChIP assay to detect the DNA expression level of the DNMT1 promoter. *N* = 3; **P < 0.01. **D** To detect the changes in protein expression levels of NHE7 or DNMT1 in macrophages with DNMT1 overexpression by WB. *N* = 3; **P < 0.01. **E** To detect the changes in mRNA levels of NHE7 in macrophages with DNMT1 overexpression by RT-qPCR. N = 3; **P < 0.01. **F**, **G** ChIP assay to validate the interaction between DNMT1 and the NHE7 promoter. **F** ChIP assay using a DNMT1 antibody showing the enrichment of DNMT1 at the NHE7 promoter in macrophages. **G** Analysis of DNA methylation levels at the NHE7 promoter region following the ChIP assay with DNMT1 antibody. *N* = 3; *P < 0.05, **P < 0.01. **H** After co-treatment with the DNA methyltransferase inhibitor 5-aza, WB was used to detect the protein level of NHE7. *N* = 3; **P < 0.01. **I** To detect the mRNA expression level of NHE7 after co-treatment with the DNA methyltransferase inhibitor 5-aza by RT-qPCR. *N* = 3; **P < 0.01.

Our RNA-seq data revealed that hypoxic EC-induced macrophage-related DEGs were enriched in gene expression regulation and methylation pathways. In vitro, we observed that exogenous lactate treatment (10 mM, 24 h) significantly increased DNMT1 and decreased NHE7 expression in macrophages (Fig. 9A). Conversely, inhibition of lactate production via OA co-treatment attenuated this effect (Fig. 9B), confirming lactate as a key mediator of DNMT1/NHE7 regulation in macrophages. To investigate the mechanism of lactate-mediated epigenetic regulation, we performed ChIP assay with an H3K18la antibody, which revealed a significant increase in H3K18la-specific enrichment at the DNMT1 promoter following lactate treatment in macrophages (Fig. 9C). Macrophages overexpressing DNMT1 (validated by WB assay, Fig. 9D) showed decreased NHE7 protein and mRNA levels (Fig. 9D, E), implicating an epigenetic modification of DNMT1 in regulating NHE7 expression. ChIP assay using DNMT1 antibodies confirmed its significant enrichment at the NHE7 (Fig. 9F), an event correlated with increased methylation of this regulatory region (Fig. 9G). Co-treatment with the DNA methyltransferase inhibitor 5-aza-2’-deoxycytidine (5-aza) significantly reversed lactate-mediated NHE7 suppression in its protein and mRNA levels (Fig. 9H, I), validating DNMT1-mediated methylation to NHE7 transcription.

**Fig. 10:**
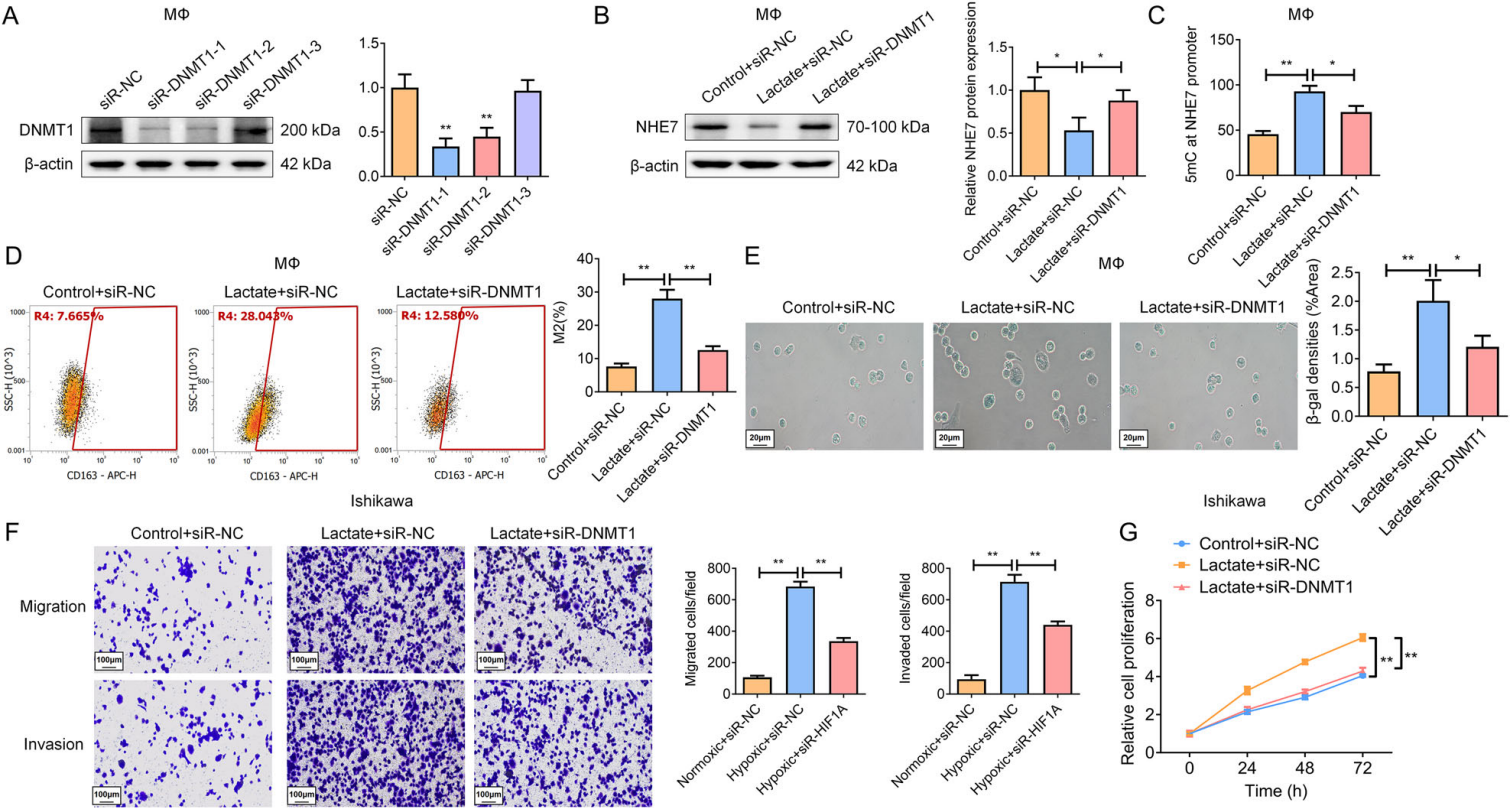
Silencing DNMT1 rescues lactate-induced NHE7 suppression and its functional consequences. **A** WB analysis confirming DNMT1 knockdown efficiency in macrophages (siR-DNMT1-1 selected for optimal knockdown efficiency). *N* = 3; **P < 0.01. **B** WB analysis of NHE7 protein levels in control (siR-NC) and DNMT1-silenced (siR-DNMT1) macrophages treated with or without lactate. N = 3; *P < 0.05. **C** Measurement of methylation levels (5mC) at the NHE7 promoter. N = 3; *P < 0.05, **P < 0.01. **D** Flow cytometry analysis of M2 macrophage polarization (CD163^+^ cells) under the indicated conditions. N = 3; **P < 0.01. **E** SA-β-gal staining to detect the senescence level of macrophages. N = 3; *P < 0.05, **P < 0.01. **F** Migration and invasion assays in Ishikawa cells upon DNMT1 knockdown. *N* = 3; *P < 0.05, **P < 0.01. **G** The proliferation ability of Ishikawa cells was detected using the MTT assay. *N* = 3; **P < 0.01.

To establish a direct causal link between lactate-induced DNMT1 activity and NHE7 suppression, we silenced DNMT1 in macrophages (Fig. 10A). While lactate treatment in control cells (Lactate+siR-NC) robustly decreased NHE7 protein expression, this effect was significantly abrogated in DNMT1-silenced cells (Lactate+siR-DNMT1) (Fig. 10B). Concordantly, DNMT1 silenced also rescued the lactate-induced hypermethylation of the NHE7 promoter, as measured by 5mC levels (Fig. 10C). Functionally, silencing DNMT1 reversed the lactate-driven promotion of M2 polarization and cellular senescence in macrophages (Fig. 10D, E). Finally, SN from Lactate+siR-DNMT1 macrophages lost the capacity to enhance EC cell proliferation, migration, and invasion that was observed with medium from Lactate+siR-NC macrophages (Fig. 10F, G). Furthermore, NHE7 overexpression in lactate-treated macrophages significantly attenuated M2 polarization (Fig. S10A), reduced senescence markers (β-gal activity and p53/p21/p16; Fig. S10B, C), and suppressed MAPK activation (p-MEK/p-ERK; Fig. S10D). Conditioned medium from these NHE7-overexpressing macrophages reversed the pro-tumorigenic effects of lactate-treated macrophage SN, inhibiting EC cell migration, invasion, and proliferation (Fig. S10E, F). Together, these data delineate a complete signaling axis wherein lactate orchestrates macrophage polarization & senescence and tumor-promoting function through H3K18la-mediated DNMT1 upregulation and subsequent NHE7 epigenetic silencing.

### NHE7 overexpression in macrophages rescues lactate-mediated tumor growth acceleration and macrophage polarization/senescence In vivo

**Fig. 11:**
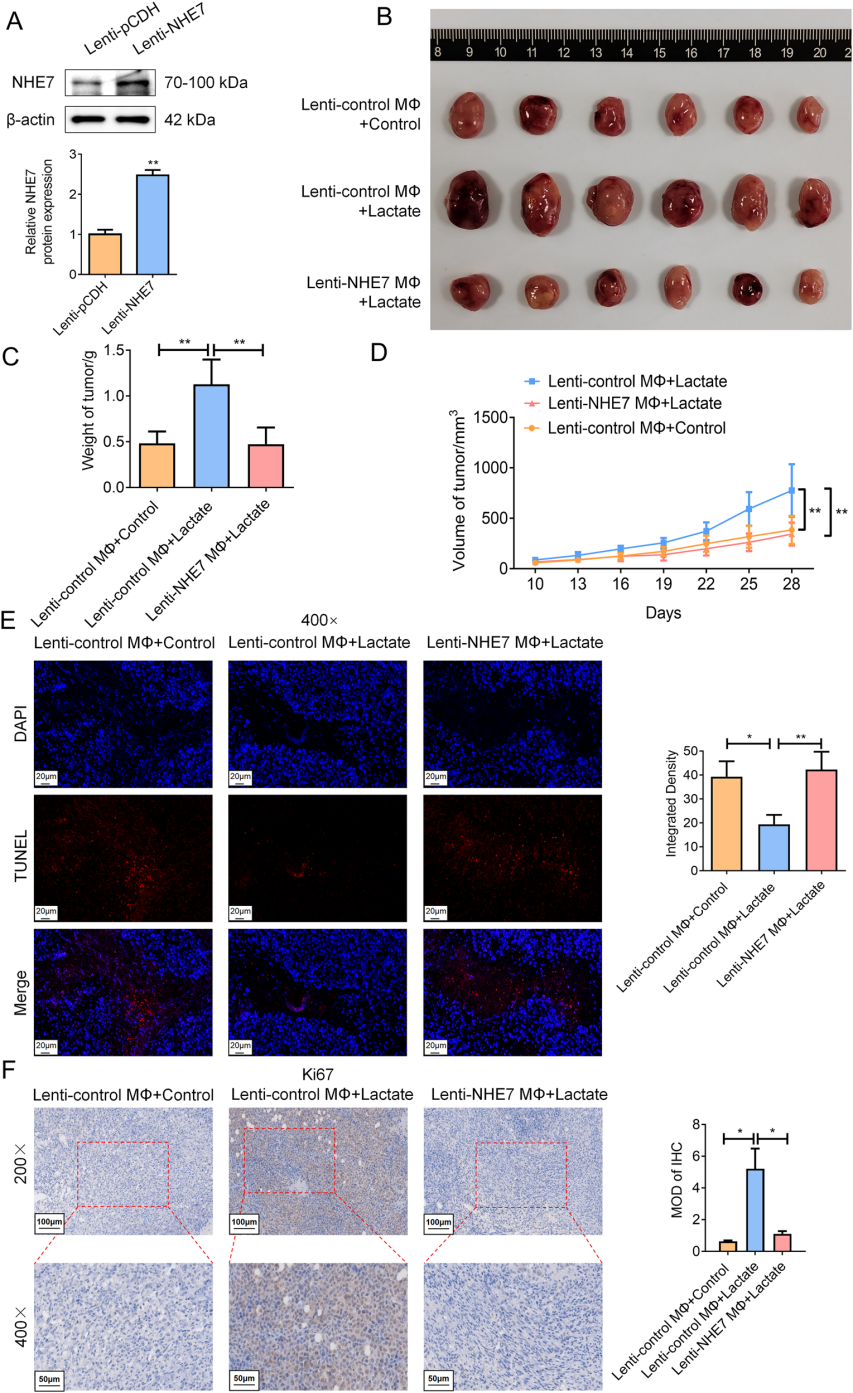
Stable NHE7 overexpression in macrophages suppresses lactate-induced tumor growth and promotes apoptosis in Ishikawa cell xenografts. We successfully generated xenograft tumors by co-inoculating NHE7-stably overexpressing macrophages (or control macrophages) with Ishikawa cells under the subcutaneous layer of nude mice. **A** To detect the efficiency of stable NHE7 overexpression in macrophages by WB. *N* = 3; **P < 0.01. **B–D** Final xenograft tumor size (**B**), tumor weight (**C**), and final tumor volume (**D**) were observed and counted. N = 6; **P < 0.01. **E** To detect cell apoptosis after treatment with lactate or co-treatment with NHE7 overexpression by the TUNEL assay. Scale bar: 20 μm; N = 3; *P < 0.05, **P < 0.01. **F** To detect the expression level of Ki67 in tumor tissues after treatment with lactate or co-treatment with NHE7 overexpression by IHC. Scale bar: 50 μm (200×), Scale bar: 20 μm (400×); *N* = 3; *P < 0.05.

**Fig. 12:**
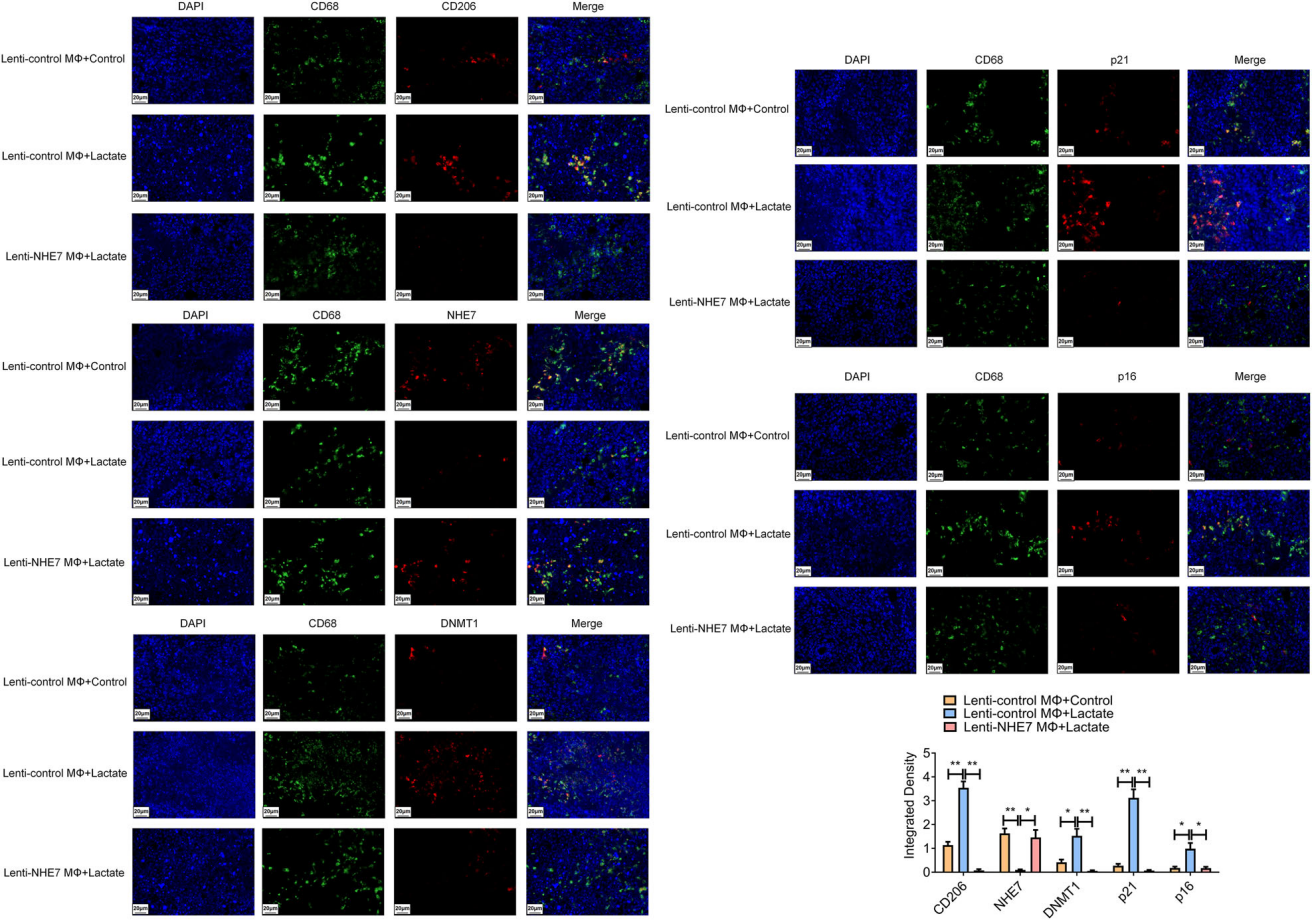
The overexpression of NHE7 combined with lactate treatment significantly weakens the performance of lactate in xenograft tumor tissues. To detect the expression levels of CD206, NHE7, DNMT1, and senescence markers (p16 and p21) in tumor tissues after treatment with lactate or co-treatment with NHE7 overexpression by IF. Scale bar: 20 μm; N = 3; *P < 0.05, **P < 0.01.

To verify our results In vivo, stable overexpression of NHE7 (Lenti-NHE7) in macrophages was successfully achieved, as confirmed by WB assay (Fig. 11A). Subcutaneous xenograft tumors were generated by co-inoculating NHE7-overexpressing macrophages (or control macrophages) with Ishikawa cells in nude mice. Post-tumor formation, lactate treatment significantly accelerated tumor growth compared to controls. Notably, NHE7-overexpressing co-transfection effectively reverted the facilitation effect of lactate treatment, as evidenced by tumor imaging at the experimental endpoint (Fig. 11B) and the record of tumor weight and volume (Fig. 11C, D). TUNEL assay demonstrated that lactate treatment reduced apoptotic cells versus control, whereas NHE7 overexpression co-treatment significantly impaired this result in xenograft tumor tissues (Fig. 11E). Additionally, IHC assays revealed elevated Ki67 expression (proliferation marker) in the lactate-treated tumor tissues. Conversely, NHE7 overexpression co-treatment significantly reversed this effect in xenograft tumor tissues (Fig. 11F). More importantly, multicolor immunofluorescence analysis of xenograft tumor tissues revealed that lactate-treated tumors exhibited increased M2 macrophage polarization (elevated CD206 fluorescence) and senescence (enhanced p21 and p16 fluorescence), accompanied by reduced NHE7 and elevated DNMT1 fluorescence. These effects were significantly reversed upon NHE7 co-overexpression (Fig. 12). Further supporting our In vitro findings, these In vivo results firmly establish that lactate drives tumor growth, inhibits apoptosis, and boosts proliferation by promoting M2 macrophage polarization and senescence via DNMT1-mediated NHE7 epigenetic silencing, while NHE7 overexpression effectively reverses these malignant effects, suggesting NHE7 as a promising therapeutic target.

## Discussion

The present study elucidated a multifaceted axis involving HIF1A-driven lactate metabolism, epigenetic regulation by DNMT1/NHE7, and M2 macrophage polarization & senescence in EC progression. Our findings revealed that hypoxia-induced HIF1A activation orchestrates metabolic reprogramming in EC cells, fostering a lactate-enriched TME that drives macrophage polarization, senescence evasion, and tumor aggressiveness, providing novel insights into the molecular mechanisms driving EC progression under hypoxic conditions.

Tumor cells’ adaptability to oxygen level changes is crucial for tumor growth [38]. Hypoxia is prevalent in the TME across diverse cancers [9, 39]. Oxygenation levels vary within tumors, leading to localized pathological hypoxic regions [40]. A central component in the cellular response to hypoxia is the transcription factor HIF1A, which orchestrates the expression of specific gene networks implicated in cellular energy production and metabolic processes [38]. Research has demonstrated that HIF1A contributes to tumor immune evasion and tumorigenesis [41, 42]. We established a robust hypoxic signature in EC, defined by 1,200 DEGs enriched in HIF1A signaling and glycolysis pathways. Crucially, HIF1A, itself a known marker of poor prognosis in solid tumors [43, 44], was assigned the highest weight in our model, cementing its central role in EC hypoxia. Additionally, we demonstrated that HIF1A is significantly upregulated in EC tissues and correlates with poor prognosis. Our differential gene expression analysis identified a cluster of genes involved in glycolysis, lactate production, and transport, suggesting a direct link between HIF1A and metabolic reprogramming in EC. In vitro assays demonstrated that HIF1A activation under hypoxia (1% O_2_) promotes glycolysis and lactate production via upregulation of glycolytic enzymes (e.g., LDHA, ENO2, HK2, PGK1, and ALDOA) in EC cells (HEC-1-A and Ishikawa), in line with its canonical role in metabolic adaptation [45]. Additionally, our findings aligned with previous studies highlighting HIF1A’s role in promoting tumor progression and metastasis under hypoxic conditions in EC [46]. The significant increase in lactate concentrations in hypoxic EC cell SN further supports HIF1A-mediated metabolic reprogramming in EC.

Beyond the known role of HIF1A in glycolysis, our work uncovered a non-canonical pathway where HIF1A-driven lactate secretion directly reprograms macrophage polarization. This process is mediated by upregulating the lactate monocarboxylic acid transporter MCT3 in EC cells and MCT1 in macrophages (MCT3: a protein that pumps out lactate; MCT1: a protein that pumps in lactate) [47]. Lactate, a byproduct of glycolysis, not only fuels tumor cell proliferation but also acts as a signaling molecule to polarize macrophages toward an M2 phenotype [14, 48, 49]. Recent research has highlighted the role of lactate in tumor-immune interactions, particularly in promoting an immunosuppressive microenvironment [50, 51]. Despite this, its communication mechanisms with macrophages in EC remain unexplored in the literature. A novel aspect of our study is the identification of the hypoxic EC cell-derived lactate shuttle mechanism in promoting M2 macrophage polarization in the EC microenvironment. Our data demonstrated that under hypoxic conditions, lactic acid produced by EC cells is pumped out via the MCT3 transporter, enabling its uptake by macrophages through MCT1; this process, driven by hypoxic EC cell-derived lactate, not only promotes macrophage polarization towards the M2 type but also increases the degree of H3K18 lactylation, suggesting that lactate modifies histone epigenetics to sustain M2 polarization, a mechanism previously implicated in macrophage immunometabolism [52]. Critically, lactate derived from hypoxic ECs serves as a central mediator that polarizes M2 macrophages, which in turn fuel a feed-forward loop to aggressively promote naive EC cell proliferation, migration, and invasion.

Our bioinformatics and functional analyses further identified NHE7, a Na^+^/H^+^ exchanger [53], as a critical regulator of hypoxic EC cell-induced macrophage polarization, senescence, and the MAPK signaling represents a key innovation. NHE7, a distinctive member of the NHE family that shuttles between the trans-Golgi network, endosomes, and plasma membrane to regulate the luminal pH of these organelles [36], is also considered a candidate molecule for proton leak regulation [54]. However, the proton-leak hypothesis regarding NHE7’s function remains controversial [55], due to conflicting reports on its opposite proton-loading effects in NHE7-transfected fibroblasts [56] and pancreatic ductal adenocarcinoma (MIA PaCa-2) cells [36], necessitating further clarification of NHE7’s contrasting functions [55]. Our data validated that macrophages derived from hypoxic ECs downregulate NHE7 expression, which modulates intracellular pH to promote their polarization toward the anti-inflammatory M2 phenotype. This polarization is crucial in determining the tissue repair and inflammatory outcomes in hypoxic conditions [57]. Additionally, NHE7’s involvement in p53/p21/p16 regulation suggests its role in the cell senescence process, potentially impacting tissue regeneration and fibrosis [58]. Furthermore, the MAPK pathway emerged as a central node integrating NHE7 and macrophage polarization & senescence. Collectively, our gain and loss-of-function studies definitively identify MAPK signaling as the central downstream effector through which NHE7 governs macrophage polarization and its subsequent pro-tumorigenic functions. This aligns with studies showing MAPK activation promotes M2 polarization [59], but our data uniquely position NHE7 as a pH-dependent regulator of MAPK signaling in macrophages. In terms of mechanism, integrated multi-omics and In vivo analyses delineate an HIF1A-driven, lactate-mediated epigenetic axis: elevated histone lactylation enhances DNMT1 expression, which subsequently silences NHE7 via promoter methylation, ultimately contributing to poor prognosis. While DNMT1 has been linked to macrophage plasticity, our study is among the first to tie its activity to metabolic cues (lactate) and NHE7 regulation. Furthermore, the In vivo experiments further confirmed our findings from the bioinformatics analysis and the In vitro experiments.

Our study offers several innovative insights into the molecular mechanisms underlying EC progression. Firstly, we identified HIF1A as a key regulator of lactate-mediated glycolytic reprogramming in EC, providing a new perspective on the role of hypoxia in tumor metabolism. Secondly, we elucidated the lactate shuttle mechanism in promoting M2 macrophage polarization, highlighting the importance of lactate in tumor-immune interactions. Thirdly, our discovery of DNMT1 methylation-mediated NHE7’s role in regulating macrophage polarization and senescence via the MAPK signaling pathway provides a new therapeutic target for EC. However, our study also has several limitations. Firstly, the In vitro and In vivo models used may not fully recapitulate the complexity of the human tumor microenvironment. Future studies should consider using patient-derived xenografts to better mimic the In vivo conditions. Secondly, while we identified several key molecules and pathways involved in EC progression, the precise mechanisms by which these molecules interact and regulate each other remain to be fully elucidated. Additional studies, including gene knockout or overexpression experiments in animal models, are needed to confirm the causal relationships observed in our study.

## Conclusion

This study demonstrates that hypoxic EC cells secrete lactate via HIF1A to induce macrophage senescence and M2 polarization through the DNMT1-NHE7 axis, accelerating tumor progression. Mechanistically, lactate upregulates DNMT1 via H3K18 lactylation, leading to epigenetic silencing of NHE7, which activates the MAPK pathway and drives pro-tumor macrophage reprogramming. Targeting the lactate shuttle and DNMT1/NHE7/MAPK axis may offer novel therapeutic strategies for EC by modulating the tumor microenvironment and immune response.

## Materials and methods

### Cell lines and cell culture

The HEC-1-A (YDT-0225), Ishikawa (YDT-0302), HEK-293T (YDT-0019), and THP-1 (YDT-0666) cell lines were obtained from INDIT Bio-Technology Co., Ltd. (Hangzhou, China). HEC-1-A cells were cultured in McCoy’s 5 A medium (Thermo Fisher, USA, 16600082), while Ishikawa and HEK-293T cells were maintained in MEM medium (BDBIO, China, C11095500BT). The culture media were supplemented with 10% fetal bovine serum (FBS; SERANA, China, s-FBS-x-015) and 1% penicillin/streptomycin (Gibco, USA, 15140-122). All cell lines were incubated at 37°C in a humidified atmosphere containing 5% CO₂ and 21% O₂ and confirmed to be free of mycoplasma contamination.

### Cell treatment and regents

Logarithmic growth phase EC cells were cultured under 1% O_2_ (hypoxia treatment) for 24 h, while cells under 21% O_2_ (normoxia treatment) served as normoxic controls. The supernatants from hypoxic EC cells were collected and used to treat macrophages for 24 h. After treatment, the macrophages were rinsed with serum-free medium and then maintained in fresh medium for an additional 24 h. The macrophage supernatants were then collected, centrifuged at 2500 rpm for 5 min to remove cell debris, and the resulting supernatants were used for subsequent functional assays.

For functional assays, cells were treated for 24 h with the lactic acid synthesis inhibitor OA (MedChemExpress, USA, HY-W013032A; 10 mM), the MAPK inhibitor U0126 (MedChemExpress, USA, HY-12031A; 10 μM), or the DNA methyltransferase inhibitor 5-aza (Selleckchem, USA, S1200; 1 μM). In separate experiments, cells were pretreated with the MAPK activator C16-PAF (MedChemExpress, USA, HY-108635; 5 μM) for 12 h. In addition, macrophages were exposed to sodium L-lactate (Selleckchem, USA, S6010; 10 mM, pH 6.8) for 24 h, with parallel control groups receiving pH-matched PBS (pH 6.8) to exclude potential confounding effects attributable to pH changes during exogenous lactate treatment.

For THP-1 cell treatment, the RPMI-1640 medium (BDBIO, China, L103-500) was additionally supplemented with 0.05 mM β-mercaptoethanol (2-ME; MedChemExpress, USA, HY-Y1311). To induce M0 macrophages, THP-1 monocytes were centrifuged, washed with PBS, and resuspended in RPMI-1640 medium. Cells (1.2 × 10^6^ per dish) were plated in 6-cm dishes containing 4 mL of medium supplemented with 100 ng/mL phorbol 12-myristate 13-acetate (PMA; Solarbio, China, 16561-29-8) for 24 h to induce differentiation. Differentiated M0 macrophages are denoted “MΦ“ throughout this study.

### WB assay

Extracted proteins (RIPA buffer: Thermo Fisher, USA, 87787) were quantified using a bicinchoninic acid (BCA) protein assay kit (Solarbio, China, PC0020), resolved by sodium dodecyl sulfate-polyacrylamide gel electrophoresis (SDS-PAGE), and transferred onto polyvinylidene difluoride (PVDF) membranes (Thermo Fisher, USA, 88585). After blocking with 5% (w/v) bovine serum albumin (BSA; Solarbio, China, A8020), membranes were successively probed with primary antibodies overnight at 4 °C and HRP-conjugated secondary antibodies for 2 h. Signals were detected using an enhanced chemiluminescence (ECL) substrate kit (Bio-Rad, USA, 1705060) and quantified (ImageJ) relative to β-actin. Antibodies are listed in Table 1.

**Table 1:** Details of antibodies.

Name	Brand	Number	Dilution rate	Applications
HIF1A	proteintech	20960-1-AP	1:5000/1:200	WB/ IHC
LDHA	proteintech	21799-1-AP	1:5000/1:200	WB/IHC
ENO2	proteintech	66150-1-Ig	1:5000/1:1000	WB/IHC
MCT3	NOVUS	NBP1-59885	1 μg/ml/1:200	WB/IHC
HK2	proteintech	22029-1-AP	1:2000/1:1000	WB/IHC
PGK1	proteintech	68035-1-Ig	1:10000/1:1000	WB/IHC
ALDOB	proteintech	18065-1-AP	1:10000/1:500	WB/IHC
β-actin	CST	4970 T	1:1000	WB
H3K18la	PTM BIO	PTM-1427RM	1:1000/1:50	WB/CHIP
H3	CST	14269S	1:1000	WB
SLC9A7	Thermo	PA5-106748	1:1000/1:200	WB/IF
SLC9A7	Elabscience	E-AB-12875	1:200	IHC
Flag	CST	14793S	1:1000	WB
p53	proteintech	21891-1-AP	1:2000	WB
p21	abcam	ab109520	1:5000/1:100	WB/IF
p16	Santa Cruz	sc-1661	1:1000/1:200	WB/IF
p-ERK	CST	4370 s	1:2000	WB
ERK	CST	4695 s	1:1000	WB
p-MEK	CST	9154 T	1:1000	WB
MEK	proteintech	11049-1-AP	1:20000	WB
DNMT1	CST	5032 T	1:1000/1:100	WB/ IF
CD68	proteintech	25747-1-AP	1:5000	IF
CD206	proteintech	18704-1-AP	1:500	IF
Ki67	Abclonal	A20018	1:500	IHC
Anti-rabbit IgG, HRP-linked Antibody	CST	7074P2	1: 2000	WB
Anti-mouse IgG, HRP-linked Antibody	CST	7076P2	1: 2000	WB
Anti-rabbit IgG Antibody	Servicebio	G1213	1:200	IHC
Tri-color Multiple Immunofluorescence Kit	Shanghai Recordbio	RC0086-23RM	1: 200	IF
CD163	Invitrogen	17-1639-42	5 µL(0.5 µg)/test	Flow cytometry
CD68	Invitrogen	12-0689-42	5 µL (0.5 µg)/test	Flow cytometry

### RT-qPCR assay

Total RNA was isolated using Trizol reagent (Ambion, China, 15596018), precipitated with isopropanol (Acros Organics, USA, 327270010), and washed with 75% ethanol. The air-dried RNA pellets were then resuspended in diethylpyrocarbonate (DEPC)-treated nuclease-free water (BioSharp, China, BL510B). Complementary DNA (cDNA) was synthesized using the RevertAid First Strand cDNA Synthesis Kit (Thermo Fisher, USA, K1622). RT-qPCR was performed with the SYBR Green Pro Taq HS Premixed qPCR Kit (Accurate, USA, AG11718), using β-actin as an internal reference gene. Relative gene expression was calculated via the 2^⁻ΔΔCT^ method. Primer sequences are provided in Table 2.

**Table 2:** Details of primer sequences.

Name	F (5’-3’)	R (3’-5’)
β-actin	AGCGGGAAATCGTGCGTG	CAGGGTACATGGTGGTGCC
SLC9A7	TCATGTATGGTGTGGTGAAG	GCAACAGCATCATTTAGGAC
HBA2	GGTCAACTTCAAGCTCCT	GTATTTGGAGGTCAGCAC
LCTL	GACGGCTACTACAAGGTCCAG	GGTCACGATGGGAGTGATGTT
DNMT1	CAATTCCGACTCGACCTATG	TCCCCTGTCCTTCTCCCT
TEKT1	AACTTGAGCAGCTTGTGAATGT	CAGCAGAGCCATAATGCCCT
SLC16A1	GGTGGAGGTCCTATCAGCAGT	CAGAAAGAAGCTGCAATCAAGC

### Cell transfection

To establish NHE7-overexpressing and DNMT1-overexpressing M0 macrophages, as well as HIF1A-overexpressing Ishikawa cells, plasmids encoding NHE7 (NM_001257291), DNMT1 (NM_001130823), HIF1A (NM_001530), and the empty pCDH vector (VT1480) were procured from Youbio (Hunan, China). The plasmids were transfected into M0 macrophages using Lipofectamine 2000 (Invitrogen, USA, 11668-019) and incubated for 24 h. For stable overexpression, recombinant lentiviral vectors (Lenti-NHE7, Lenti-HIF1A, and Lenti-control) were also obtained from Youbio. Lentiviral particles were generated by transfecting HEK-293T cells with Lenti-NHE7, Lenti-HIF1A, or Lenti-control, after which the viral supernatant was collected, filtered, and used to infect M0 macrophages or EC cells (for HIF1A overexpression). After 48 h, the medium was replaced, and puromycin (1 µg/mL; Yuanye, China, S17055) was added to select stable clones.

For NHE7 knockdown, short hairpin RNAs (shR-NHE7) and a negative control shRNA (shR-NC) were purchased from Tsingke Biotechnology (Beijing, China) and transfected using Lipofectamine 2000 for 24 h. In addition, small interfering RNAs (siRNAs) targeting HIF1A (siR-HIF1A-1/-2/-3), MCT1 (siR-MCT1-1/-2/-3), MCT3 (siR-MCT3-1/-2/-3), and DNMT1 (siR-DNMT1-1/-2/-3) were obtained from Tsingke and similarly transfected for 24 h using Lipofectamine 2000. Transfection efficiency was verified by WB assays. The detailed sequences of siRNAs and shRNAs are listed in Table 3.

**Table 3:** Detailed sequences of siRNAs and shRNAs.

Name	Sense	Anti-sense
shR-NC	GGTTCTCCGAACGTGTCACGT	ACGTGACACGTTCGGAGAACC
shR-NHE7	GACTGAACACTCACGCCTTTG	CAAAGGCGTGAGTGTTCAGTC
siR-NC	UUCUCCGAACGAGUCACGUTT	ACGUGACUCGUUCGGAGAATT
siR-HIF1A-1	CCGCTGGAGACACAATCATAT	ATATGATTGTGTCTCCAGCGG
siR-HIF1A-2	CCAGTTATGATTGTGAAGTTA	TAACTTCACAATCATAACTGG
siR-HIF1A-3	CGGCGAAGTAAAGAATCTGAA	TTCAGATTCTTTACTTCGCCG
siR-MCT1-1	GCAGGGAAAGATAAGTCTAAA	TTTAGACTTATCTTTCCCTGC
siR-MCT1-2	GCTCCGTATTGTTTGAAACAT	ATGTTTCAAACAATACGGAGC
siR-MCT1-3	CCAGCGAAGTGTCATGGATAT	ATATCCATGACACTTCGCTGG
siR-MCT3-1	CGTCTACATGTACGTGTTCAT	ATGAACACGTACATGTAGACG
siR-MCT3-2	GCTCATACAGGAGTTTGGGAT	ATCCCAAACTCCTGTATGAGC
siR-MCT3-3	CGTCTACATGTACGTGTTCAT	ATGAACACGTACATGTAGACG
siR-DNMT1-1	GCCGAATACATTCTGATGGAT	ATCCATCAGAATGTATTCGGC
siR-DNMT1-2	CGACTACATCAAAGGCAGCAA	TTGCTGCCTTTGATGTAGTCG
siR-DNMT1-3	CGAGAAGAATATCGAACTCTT	AAGAGTTCGATATTCTTCTCG

### Methyl thiazolyl tetrazolium (MTT) assay

Cell proliferation was assessed using the MTT assay. EC cells (3 × 10³ cells/well) were seeded in 96-well plates and, after adherence, cultured under normoxic or hypoxic conditions with or without macrophage supernatant. Ishikawa and HEC-1-A cells were incubated anaerobically for 24 h, followed by an additional 0-72 h culture period. MTT solution (50 μL; Sigma, M2128) was then added for 3 h, after which formazan crystals were dissolved in DMSO. Absorbance at 570 nm was measured using a microplate reader to determine cell viability.

### Flow cytometry assay

Flow cytometry was used to assess M2 macrophage polarization by measuring CD163 surface expression. After treatment, cells were enzymatically detached, fixed with 4% paraformaldehyde (Biosharp, China, BL539A), and blocked with 5% BSA. Cells were then immunostained with APC-conjugated anti-CD163, followed by a secondary antibody, and washed twice with PBS. Fluorescence was measured on an Attune NxT flow cytometer. In parallel, intracellular pH was assessed in cells loaded with BCECF-AM using the BL1-A channel. Detailed antibody information is listed in Table 1.

### Cell migration and invasion

Cell migration and invasion were evaluated using Transwell chambers (COSTAR, USA, 3422). Treated EC cells were resuspended in serum-free medium and seeded into the upper chamber (HEC-1-A: 1 × 10⁵ cells/well; Ishikawa: 8 × 10⁴ cells/well), with 10% FBS medium added to the lower chamber. After 24 h, non-migrated or non-invaded cells were removed, and membranes were fixed with methanol and stained with crystal violet (Aladdin, China, C110703). Images were captured using a microscope. For invasion assays, upper chambers were pre-coated with 100 μL Matrigel (Corning, USA, 356234) for 30 min before cell seeding.

### Lactic acid (LA) detection assay

L-LA concentrations in the culture supernatants of treated EC cells and in the supernatant of animal tissue homogenates were measured using a human LA ELISA kit (Solarbio, China, BC2230) following the manufacturer’s instructions. The working solution was incubated at 37 °C for 5 min, after which 30 μL of sample or standard was added and allowed to react at 37 °C in the dark for 10 min. Absorbance at 570 nm was then recorded using a SpectraMax M5 microplate reader.

### In vivo tumor xenograft study

All animal procedures were performed in accordance with China’s Guidelines for the Care and Use of Laboratory Animals and approved by the Ethics Committee of Women’s Hospital, Zhejiang University School of Medicine (Approval No. AE20250014). Thirty female BALB/c nude mice (6-8 weeks old; Charles River, Beijing) were maintained under specific pathogen-free (SPF) conditions (22 °C ± 1 °C, 50 ± 10% humidity, 12 h light/dark cycle) with ad libitum access to food and water. Following a 7-day acclimatization, 18 mice were randomized into three groups (n = 6): [1] Ishikawa cells + Lenti-control macrophages + saline (Lenti-control MΦ + control); [2] Ishikawa cells + Lenti-control macrophages + lactate (Lenti-control MΦ + lactate); [3] Ishikawa cells + Lenti-NHE7 macrophages + lactate (Lenti-NHE7 MΦ + lactate). Twelve additional mice were divided into two groups (n = 6): [1] Lenti-control Ishikawa cells + macrophages (Lenti-control + MΦ); [2] Lenti-HIF1A Ishikawa cells + macrophages (Lenti-HIF1A + MΦ). Tumors were established by subcutaneous co-injection of Ishikawa cells (4 × 10⁶) and macrophages (4 × 10⁶) in 200 μL PBS into the right flank. Once tumors reached 50 mm³ (calculated as long diameter × short diameter² × 1/2), mice received intratumoral injections of 20 mM sodium L-lactate (50 μL; Selleckchem, USA, S6010) or saline twice weekly. Tumor growth was monitored until volumes reached 1000 mm³, at which point mice were euthanized. Excised tumors were weighed, photographed, and processed for downstream analyses.

### ELISA assay

Macrophages with NHE7 overexpression or knockdown were trypsinized and plated. After 24 h, supernatants were collected to measure SASP factors using commercial ELISA kits (MEIMIAN, China). Standards and samples were added to pre-coated 96-well plates and incubated with primary antibodies (IL-10, No.: MM-006H1; TGF-β, No.: MM-1774H1) at 37 °C for 1 h, followed by HRP-conjugated secondary antibodies for 30 min. After substrate addition and reaction termination, absorbance at 450 nm was measured, and concentrations were calculated from standard curves.

### β-Galactosidase staining

Cell senescence was evaluated via β-galactosidase staining. Macrophages were fixed in 1% glutaraldehyde for 15 min at room temperature and washed three times with ice-cold PBS. Cells were then incubated overnight at 37 °C in the dark with freshly prepared staining solution containing X-gal (Beyotime, China, C0602). After three PBS washes, blue-stained cells were imaged under a light microscope.

### TUNEL staining

Cell apoptosis in xenograft tumor tissues was evaluated using the TUNEL assay. Briefly, paraffin-embedded tissue sections were deparaffinized in xylene and rehydrated through a graded ethanol series. After repair with proteinase K (Servicebio, China, G1205), the TUNEL reaction mixture (Servicebio, China, G1501) was applied, and sections were incubated in a humidified chamber at 37 °C for 1 h in the dark. Nuclei were counterstained with DAPI (Servicebio, China, G1012) for 10 min. Slides were dehydrated, cleared, and mounted. Apoptotic cells were visualized and imaged under a fluorescence microscope.

### IHC staining

IHC was performed to assess target protein expression in clinical samples (*N* = 20) or tumor tissues (*N* = 3). Samples were fixed in 4% paraformaldehyde for 1 week, dehydrated with ethanol/xylene, and paraffin-embedded. 5 μm sections were deparaffinized, rehydrated, and antigen-retrieved. Primary antibodies (detailed in Table 1) were applied overnight, followed by secondary antibody incubation and Hematoxylin (Servicebio, China, G1004) staining. Expression was observed and compared with controls under a microscope.

### ChIP assay

ChIP assays were performed to examine histone lactylation and DNA methylation. For analysis of H3K18 lactylation on the DNMT1 promoter in lactate-treated macrophages, cells were fixed with 1% formaldehyde, lysed, and sonicated to obtain 200-1000 bp chromatin fragments. After centrifugation, chromatin was precleared with blocked Protein A/G sepharose beads to minimize nonspecific binding, followed by immunoprecipitation with H3K18la antibodies (Table 1). DNMT1 binding to the NHE7 promoter was assessed in parallel using DNMT1 antibodies. After sequential washes, DNA-protein complexes were eluted, crosslinks reversed, and DNA purified using a DNA Cleanup Kit (Promega, USA, AS1450). To further evaluate NHE7 promoter methylation, methylated DNA was enriched using the EpiQuik™ Methylated DNA Immunoprecipitation (MeDIP) Kit (Epigentek, USA, p-1015), and methylation levels were subsequently quantified by qPCR. The same procedures were applied to tumor tissues derived from Lenti-HIF1A and Lenti-pCDH xenografts to determine DNMT1 promoter H3K18la enrichment and NHE7 promoter methylation levels In vivo. qPCR was performed on ChIP- or MeDIP-enriched DNA using primers targeting the DNMT1 promoter (Forward: GAATAAGGAGAGAGAAGTCA; Reverse: CCCAACTAAAGAGGTTTTG) and NHE7 promoter (Forward: TCTGGCGTCCCCTTTCGG; Reverse: TGTTGCCCTCGGGTCTGC). Data were normalized to input chromatin and presented as fold enrichment relative to IgG controls.

### Detection of DNA methylation

To assess NHE7 promoter methylation, genomic DNA was extracted from treated macrophages. Methylated DNA fragments containing 5-methylcytosine (5mC) were selectively enriched using the Methylamp Methylated DNA Capture Kit (Epigentek, USA, p-1025). The methylation status of the NHE7 promoter was subsequently quantified using the EpiQuik™ Quantitative PCR Fast Kit (Epigentek, USA, p-1029). qPCR signals were normalized to input DNA to allow comparative analysis of methylation levels across samples.

### Immunofluorescence (IF) staining

Xenograft tissues, along with clinical EC or normal tissues, were fixed in 4% paraformaldehyde for 20 min, permeabilized with 0.1% Triton X-100 (Beyotime, China, P0096) for 5 min, and blocked with 5% BSA for 90 min at 37 °C. The tissues were then incubated overnight at 4 °C with primary antibodies against CD68 (listed in Table 1). After washing, samples were treated with HRP-conjugated secondary antibodies for 1 h, followed by application of a three-color mIF kit (RecordBio^®^, China, RC0086-23RM). Subsequent to antibody elution and serum blocking, a second round of overnight incubation was performed using target antibodies specific to CD206, NHE7, DNMT1, p21, and p16 (Table 1). This was followed by another treatment with HRP-conjugated secondary antibodies and multiplex labeling. Nuclei were counterstained with DAPI (20 μg/mL; Servicebio, China, G1012) for 5 min. Finally, images were acquired using a Leica STELLARIS8 confocal microscope.

### Bioinformatics analysis

RNA-seq data of EC were downloaded from the TCGA database (https://portal.gdc.cancer.gov/), and tumor samples (n = 194) were selected for further analysis. A curated list of classical hypoxia-related genes was collected, and hypoxia enrichment scores for each sample were calculated using the “ssGSEA” algorithm implemented in the R package GSVA (v1.50.5). Based on the median hypoxia score, samples were stratified into high- and low-score groups. Boxplots of scores between sample groups were generated using the “ggplot2” package. Differential expression analysis between the two groups was performed using the “Limma” package, with significance thresholds set at |log_2_FC | > 1 and *p* < 0.05. Volcano plots were generated using “ggplot2”. KEGG and GO enrichment analyses for these DEGs were carried out using the “clusterProfiler” package. To identify genes most strongly associated with hypoxia activation, the Least Absolute Shrinkage and Selection Operator (LASSO) regression algorithm was applied using the “glmnet” package. For each sample, the hypoxia-related gene expression matrix (X) and hypoxia score vector (y) were used as input. LASSO with L1 regularization was employed to shrink coefficients and select informative variables while minimizing overfitting. Non-zero coefficients derived from the trained model were regarded as gene weights, and weight bar plots were visualized using “ggplot2”.

Transcriptomic and clinical data for EC were retrieved from the TCGA database. HIF1A expression (tumor versus normal tissues) was compared via “ggplot2” of the R package to generate boxplots. Patients were stratified by median HIF1A expression into high/low groups for Kaplan-Meier survival analysis using the “survival” and “survminer” packages. DEGs between HIF1A subgroups were identified via the “limma” package (*p*-value < 0.05 & |log_2_FC | >1), visualized by volcano plots (“ggplot2”) and heatmaps (“pheatmap”). Functional enrichment analysis of DEGs was performed using “clusterProfiler” for GO and KEGG pathways. Boxplots illustrating the expression of lactate production-, lactate transport-, and glycolysis-related genes in HIF1A subgroups were generated using the R package “ggplot2” and statistical comparisons between groups performed using the “Wilcoxon” test via the “ggpubr” package.

Single-cell data from five EC samples (GSM5276933, GSM5276934, GSM5276935, GSM5276936, and GSM5276937) downloaded from the GEO database (https://www.ncbi.nlm.nih.gov/geo/) were processed using “Seurat V5” in R. After quality control and cell filtering, data integration was performed via “Harmony”, followed by dimensionality reduction using Principal Component Analysis (PCA). Cell clusters were identified through “Louvain” community detection and visualized via UMAP, with manually annotated cell types. Tumor cells of single-cell data were classified into HIF1A_High or HIF1A_Low groups based on HIF1A expression. Violin plots of HIF1A expression in tumor cells of single-cell data were generated using “ggplot2”. Cells were stratified into HIF1A_High and HIF1A_Low groups based on HIF1A expression. UMAP visualization, bar plot (cells’ proportion of inter-group), and bubble plot (HIF1A expression of inter-group) were created via “ggplot2”. Macrophage-specific DEGs between HIF1A subgroups were identified using Seurat’s “FindMarkers” function, followed by GO/KEGG analysis with “clusterProfiler”.

Additionally, macrophages of single-cell data were re-clustered, artificially annotated, and visualized using UMAP. Re-clustered macrophages were stratified into MCT1/NHE7-High and MCT1/NHE7-Low subgroups based on median expression thresholds using “FetchData”. Inter-group UMAP plots for macrophages and violin plots for inter-group MCT1/NHE7 expression were generated using “ggplot2”. M1/M2 polarization was quantified using the “AddModuleScore” function with M1/M2 marker genes, and inter-subgroup differences in scores across NHE7 subgroups (high versus low) were visualized via box plots (“ggplot2”) and statistically assessed using the “Wilcoxon” test (“ggpubr”). M1/M2 proportions in MCT1 subgroups were quantified via bar plots (“ggplot2”). Macrophage-related DEGs within MCT1/NHE7 subgroups were identified via Seurat’s “FindMarkers”, followed by GO/KEGG analysis with “clusterProfiler”. Furthermore, macrophage-related DEGs in MCT1 subgroups were visualized as volcano plots using “ggplot2”.

Macrophages treated with hypoxic EC cell supernatant underwent RNA-seq. DEGs were analyzed using “limma” (*p*-value < 0.05 & |log2FC | >1) and visualized via volcano plots and heatmaps. Lactate metabolism genes (MCT1, MCT2, MCT3, and MCT4) were evaluated for expression differences using boxplots and “Wilcoxon” tests.

To further investigate the relationship between gene expression and clinical characteristics, one-way analysis of variance (ANOVA) was performed for each gene to assess differences in expression across clinical subgroups (age and tumor stage). The analysis was conducted using the “aov()” function in the R stats package, and boxplots were generated with “ggplot2” to visualize expression differences among groups.

For correlation analysis, expression matrices of selected genes were extracted and transformed using log_2_(FPKM + 1) to approximate normality. Pearson correlation coefficients were calculated using the “cor()” function, and significance was assessed using “cor.test()”. Multivariate Cox proportional hazards regression was performed using “coxph()” (survival package), with expression levels of the four target genes used as predictors and OS or DFS as outcome variables. To infer immune cell composition in the TME, “ssGSEA” (GSVA package) was applied to quantify immune infiltration scores for each sample. Samples were divided into high- and low-expression groups for NHE7, DNMT1, and HIF1A based on the median expression of each gene. Group comparisons were conducted using “t.test()”, evaluating differences in immune cell abundance.

ScRNA-seq data (GSE173682) containing 11 EC samples were downloaded from the GEO database (https://www.ncbi.nlm.nih.gov/geo/query/acc.cgi?acc=GSE173682). Raw 10x Genomics matrices were processed using “Seurat v5.1”. Data preprocessing included quality control filtering, normalization, integration, dimensionality reduction, and visualization. All samples were integrated using the “IntegrateLayers” function, followed by PCA using “RunPCA”. UMAP was applied for visualization and graph-based clustering using the top 30 principal components. Cell clusters were annotated according to canonical marker genes. InferCNV (https://github.com/broadinstitute/inferCNV) was used to identify large-scale chromosomal copy number variations (CNVs) from scRNA-seq data, with normal endothelial cells and fibroblasts serving as references. M2 macrophages were extracted and reclustered using the “FindClusters()” function in Seurat. Expression patterns of DNMT1 and NHE7 were mapped across these subpopulations.

### Statistical analysis

To ensure the reliability of our findings, each experiment was conducted at least three times. Data processing was performed using GraphPad 6.0 software (USA, SCR_002798), with T-tests for comparisons between two groups and one-way ANOVA for multiple group comparisons. Cohen’s d method defined the effect size calculations in the In vivo experiments (https://calculatorfree.net/zh-cn/math/effect-size-calculator.html). Data were presented as mean ± standard deviation (SD). Statistical significance was set at *p < 0.05 and **p < 0.01.

Conflict of Interest The authors declare no potential conflicts of interest.

## Materials availability

All data supporting this study are contained within this article and its Supplementary Information, including Supplementary Figures, Supplementary Table, PCR original data (Supplementary Material 1), uncropped blots (Supplementary Material 2), and the confirmation of publication and licensing rights for graphical abstract, created with BioRender.com (Supplementary Material 3). Additional data are available from the corresponding author upon reasonable request.

## Supplementary information

Supplementary Information

## Data Availability

All data supporting this study are contained within this article and its Supplementary Information, including Supplementary Figures, Supplementary Table, PCR original data (Supplementary Material 1), uncropped blots (Supplementary Material 2), and the confirmation of publication and licensing rights for graphical abstract, created with BioRender.com (Supplementary Material 3). Additional data are available from the corresponding author upon reasonable request.
