# Supplementary material for: Lactate transmission from hypoxic tumor cells promotes macrophage senescence and M2 polarization via the DNMT1-NHE7 axis to accelerate endometrial cancer progression
Source: Cell Death Dis. 2026 Jan 30;17(1):185. doi: 10.1038/s41419-026-08411-y (PMC12876988; doi:10.1038/s41419-026-08411-y)
Supplement: Supplementary file 1 — Supplementary Information [file 41419_2026_8411_MOESM1_ESM.pdf]

Supplementary Figures:

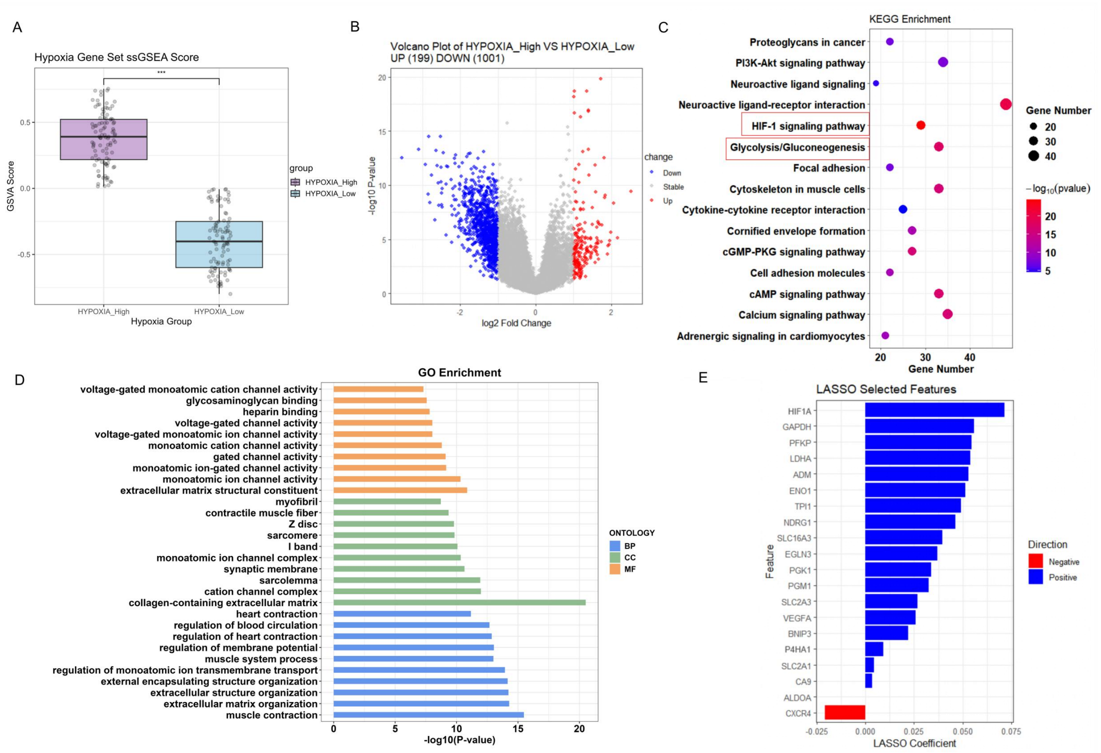

Figure S1. Hypoxia score-based stratification and functional characterization of EC. Collected classic hypoxia-related genes and applied the ssGSEA algorithm to score each sample for hypoxia-related gene sets. 194 tumor patients were divided into High- and Low-score groups based on the median hypoxia-related gene set score within the tumors. (A) A boxplot visualizing the score distribution between these groups was generated using the ggplot2 package. (B) Volcano plot displaying DEGs between hypoxia_High and Low EC groups from the TCGA cohort. (C-D) Functional enrichment analysis of hypoxia-associated DEGs for (C) KEGG pathways and (D) GO biological processes. (E) Gene weights in the LASSO regression model used to derive the hypoxia score, highlighting HIF1A as the feature with the highest contribution.

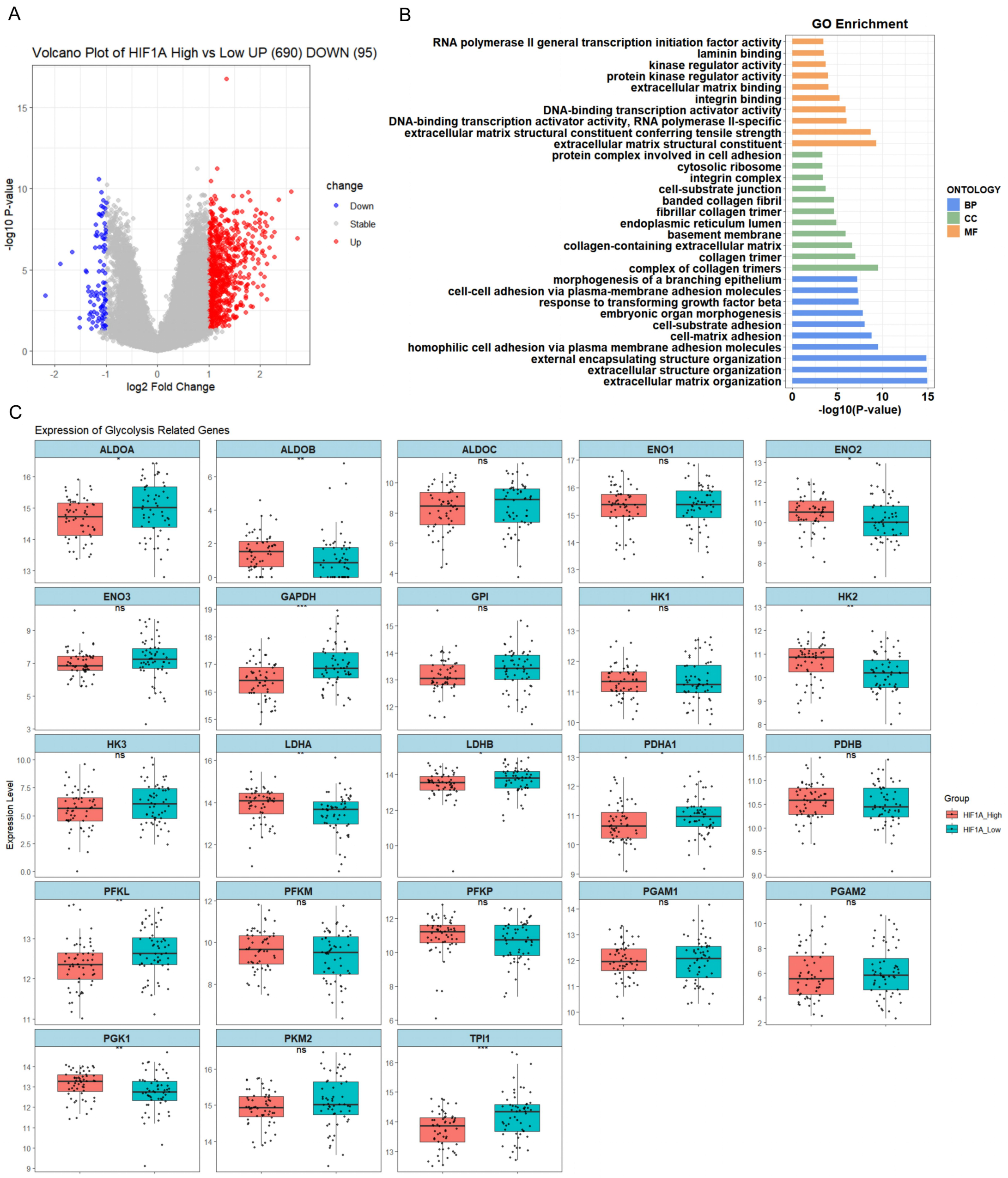

Figure S2. DEGs and the expression of glycolysis-related enzymes were analyzed in EC with high/low HIF1A expression. (A) A volcano plot depicting DEGs in EC stratified by High and Low HIF1A expression. (B) Biological processes of DEGs enriched by HIF1A in EC were clarified through GO enrichment analysis. (C) Box plots were used to analyze the expression of glycolysis-related genes in EC cells with HIF1A_high or _low expression. *$P < 0.05$, **$P < 0.01$.

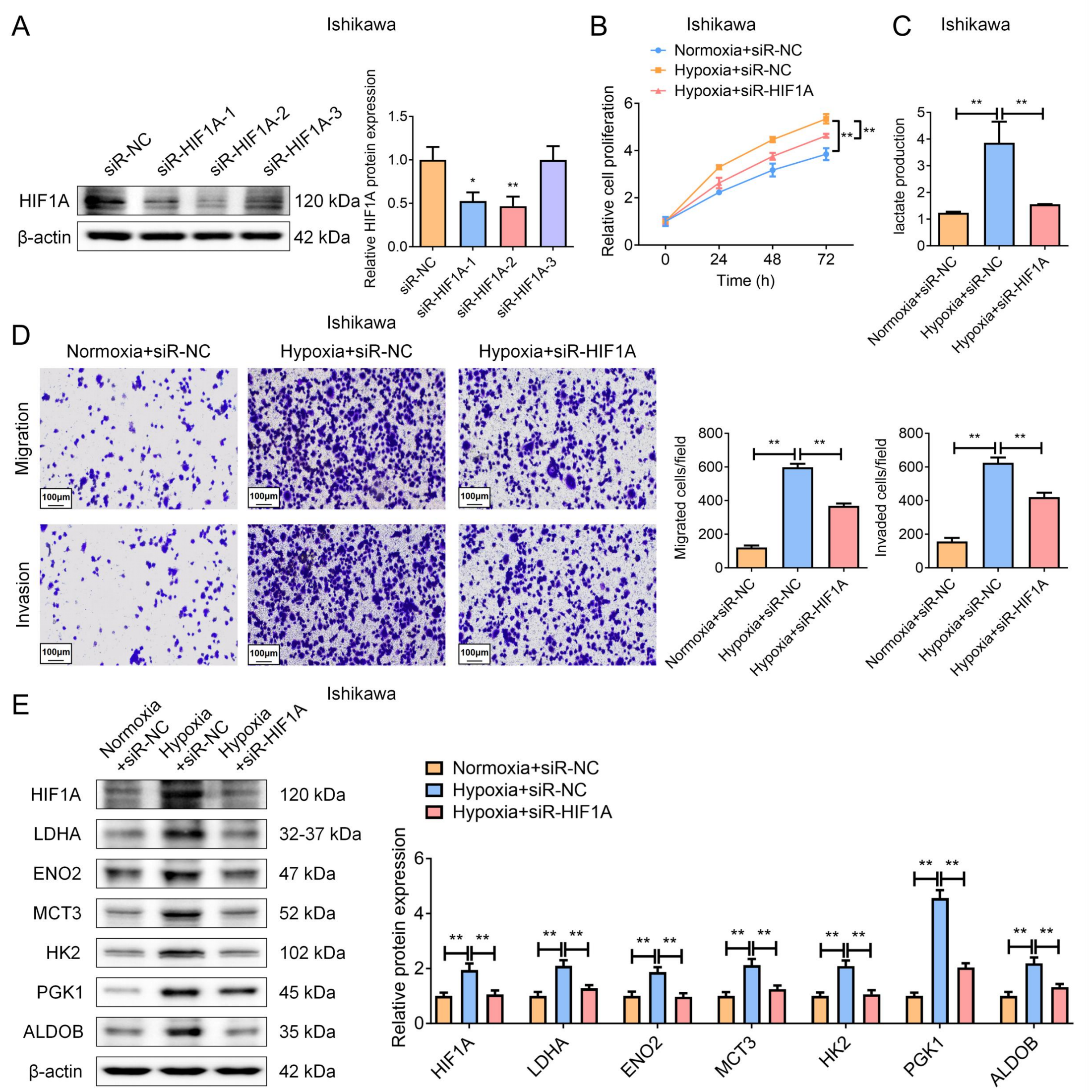

Figure S3. Functional validation of HIF1A in hypoxia-driven oncogenic phenotypes. (A) Validation of HIF1A knockdown efficiency in Ishikawa cells via siRNA. (B-E) Assessment of phenotypic and molecular changes under hypoxia following HIF1A silencing: (B) cell proliferation, (C) lactate level in the cell SN, (D) migration and invasion capabilities (Scale bar: 100 μm), and (E) expression of key glycolytic proteins by WB. Experimental groups: Normoxic + siR-NC (control), Hypoxic + siR-NC, Hypoxic + siR-HIF1A. N = 3; *$P < 0.05$, **$P < 0.01$.

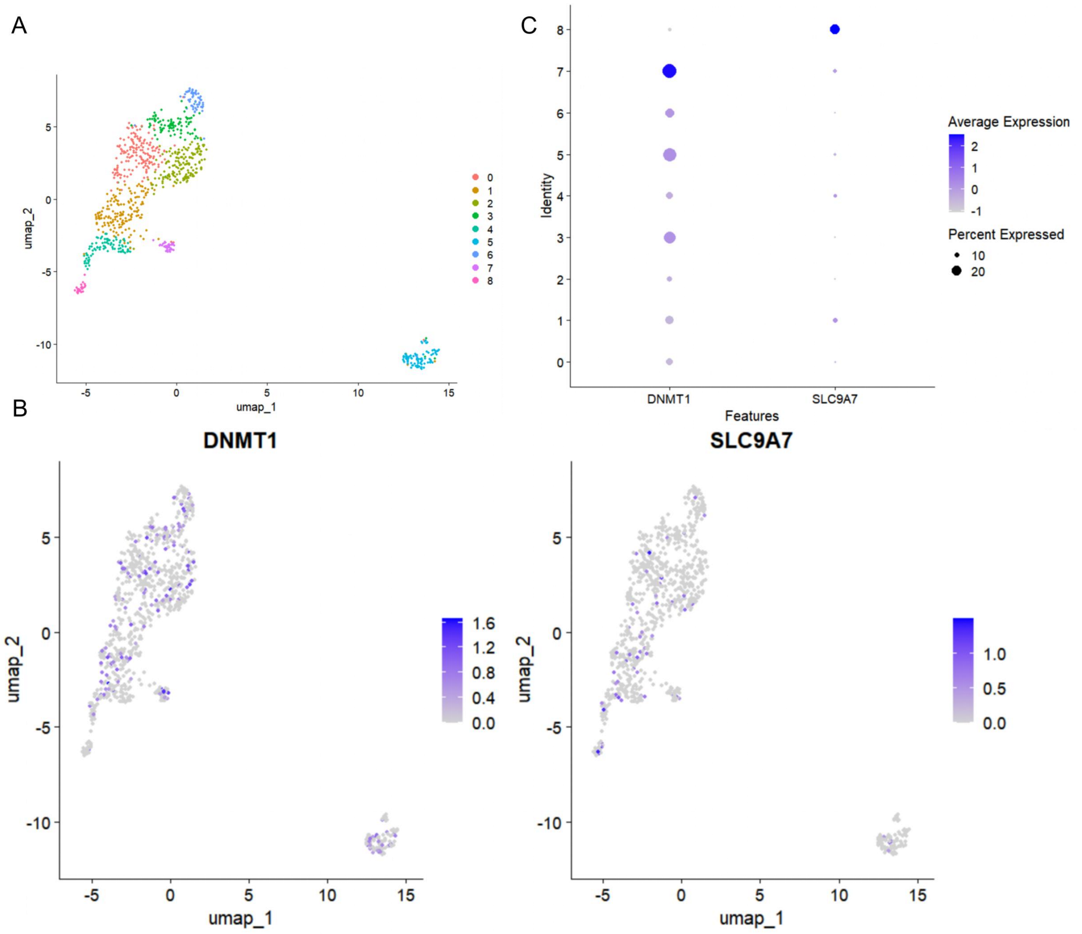

Figure S4. ScRNA-seq Analysis Identifies M2 Macrophage Subpopulations Expressing DNMT1 and NHE7 in EC. (A) UMAP visualization of cellular heterogeneity in 11 human EC samples (GSE173682 dataset), revealing distinct cell clusters. (B) UMAP visualization of nine subpopulations identified through re-clustering of M2 macrophages. (C) Feature plots displaying the expression patterns of DNMT1 and NHE7 across the M2 macrophage subpopulations.

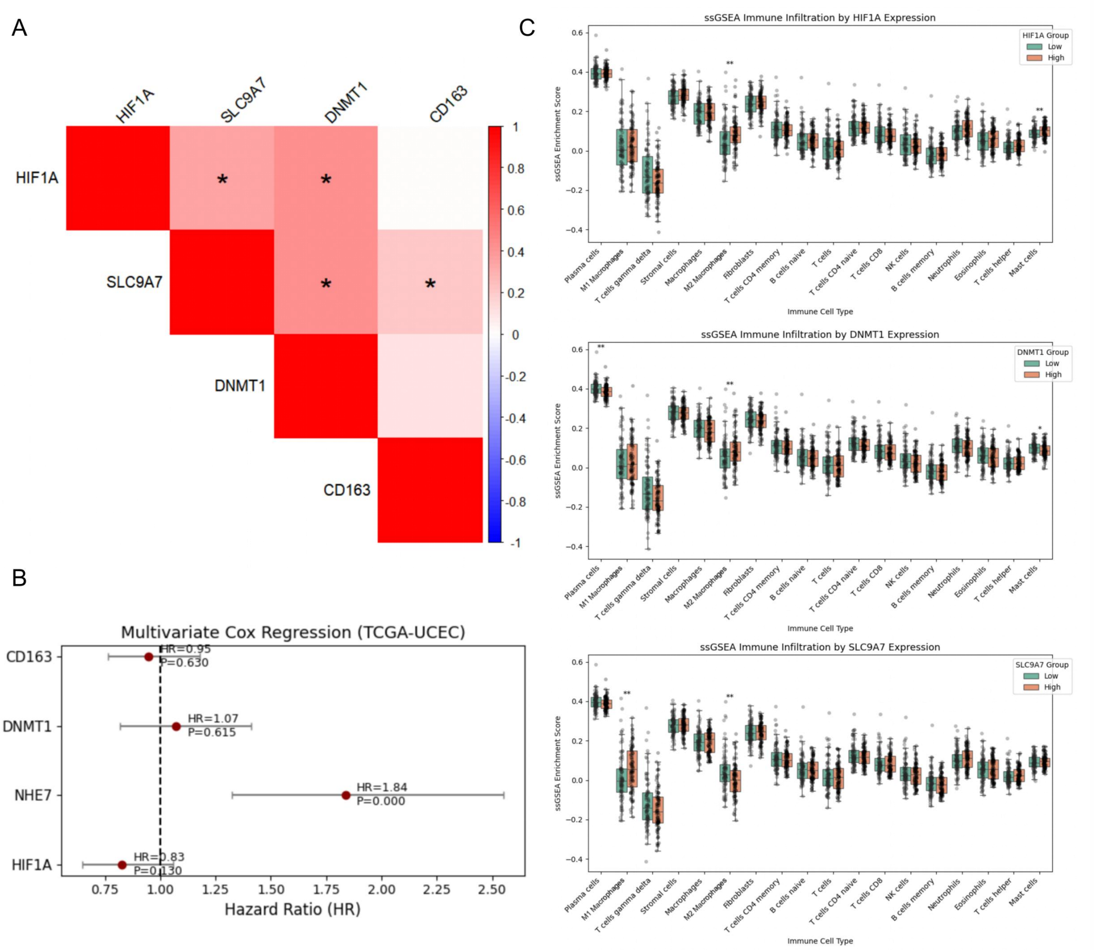

Figure S5. Correlation and Prognostic Impact of HIF1A, NHE7, DNMT1, and CD163 in TCGA EC Cohort. (A) Correlation analysis in TCGA EC samples shows the relationships between the expression levels of HIF1A, NHE7, DNMT1, and CD163. (B) Multivariate Cox regression analysis evaluating the impact of HIF1A, NHE7, DNMT1, and CD163 expression on overall survival (OS) and disease-free survival (DFS). (C) Single-sample GSEA (ssGSEA) reveals the association between the expression of HIF1A, DNMT1, NHE7, and the level of M2 macrophage infiltration.

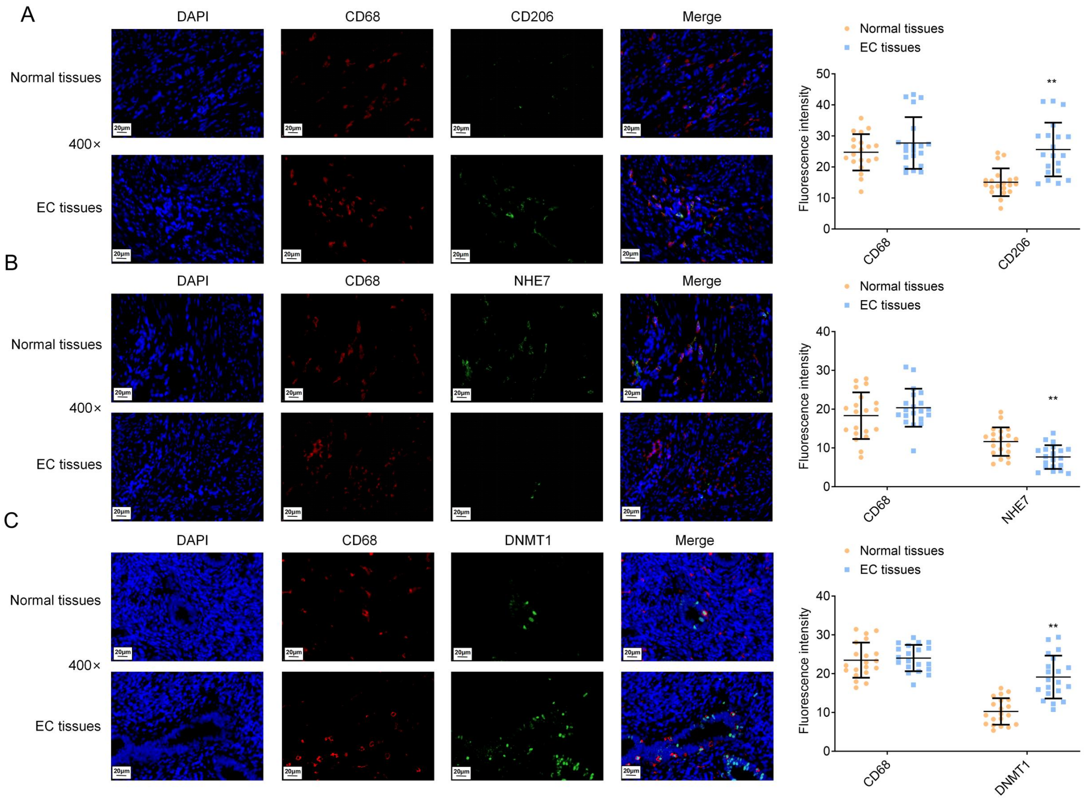

Figure S6. Multiplex immunofluorescence validates DNMT1 and NHE7 expression patterns and their associations with M2 macrophage infiltration. (A&B&C) Multiplex Immunofluorescence staining was used to detect the expression level of NHE7, DNMT1, and CD206 in human endometrial carcinoma tissues and matched adjacent normal tissues.

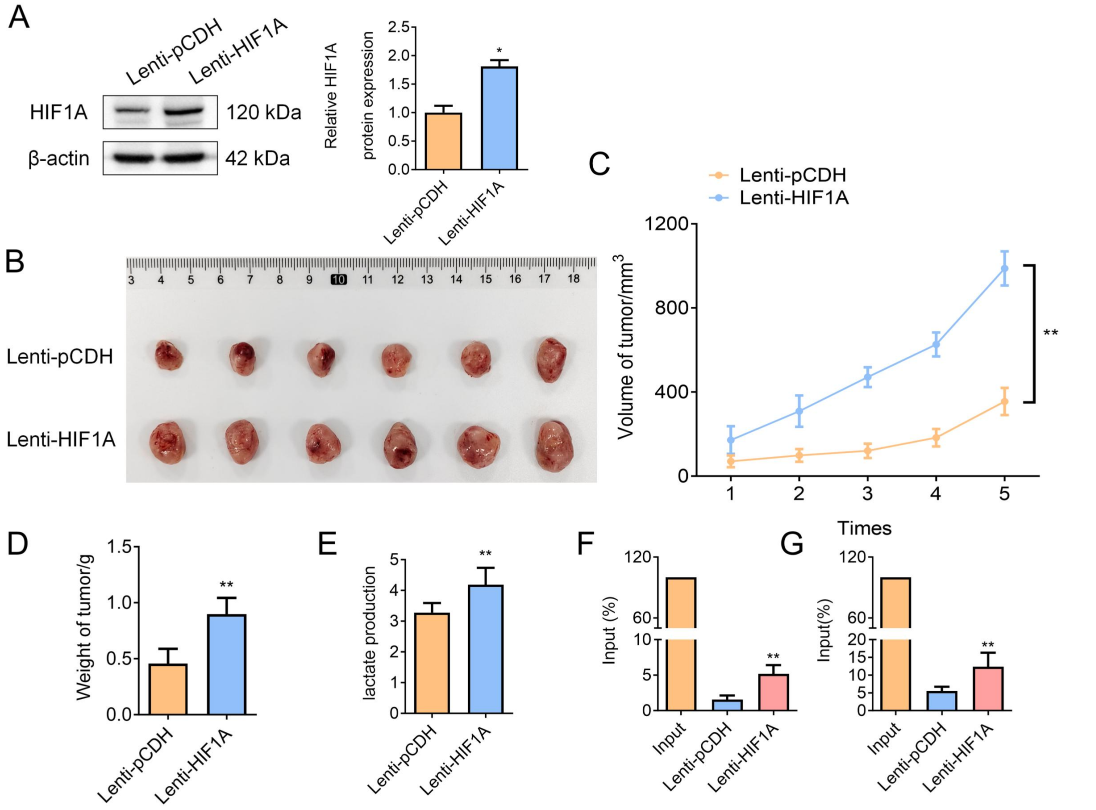

Figure S7. *In vivo* assessment of HIF1A overexpression in a subcutaneous xenograft model and associated epigenetic analyses. We successfully generated xenograft tumors by inoculating HIF1A-stably overexpressing Ishikawa cells under the subcutaneous layer of nude mice. (A) To detect the efficiency of stable HIF1A overexpression in Ishikawa cells by WB. N = 3; $^{**}P < 0.01$. (B-D) Final xenograft tumor size (B), tumor weight (C), and final tumor volume (D) were observed and counted. N = 6; $^{**}P < 0.01$. (E) Lactate levels were measured in tumor tissue to assess metabolism. N = 3; $^{**}P < 0.01$. (F) ChIP assay quantifying the enrichment of histone lactylation on the DNMT1 promoter in tumor tissues. N = 3; $^{**}P < 0.01$. (G) Analysis of the methylation level on the NHE7 promoter in tumor tissues. N = 3; $^{**}P < 0.01$.

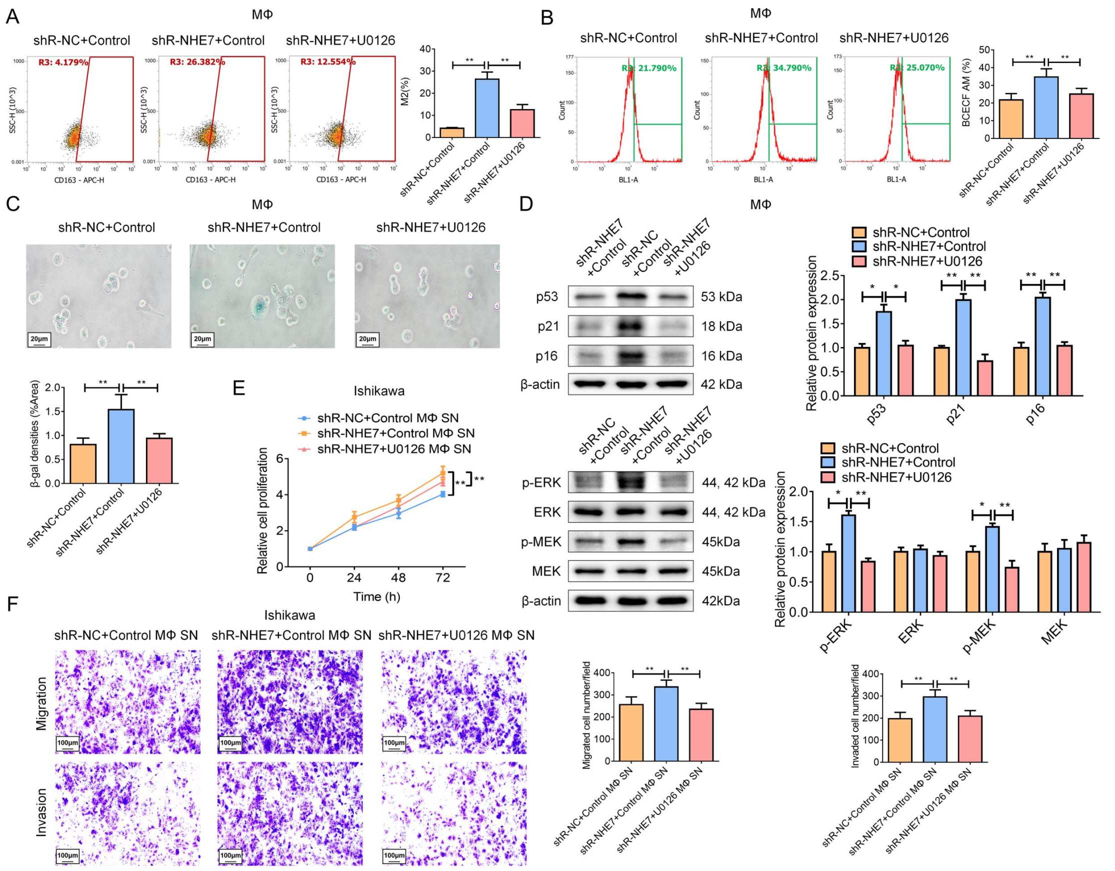

Figure S8. MAPK pathway inhibition rescues the effects of NHE7 silencing in macrophages. (A) To detect the expression of CD163 in NHE7-silenced macrophages treated with the MEK inhibitor U0126 by flow cytometry. N = 3; $^{**}P < 0.01$. (B) Flow cytometry was used to detect the intracellular pH in NHE7-silenced macrophages treated with the MEK inhibitor U0126. (C) SA-β-gal staining to detect the senescence level of macrophages after U0126 administration in NHE7-silenced macrophages. N = 3; $^{**}P < 0.01$. (D) To detect the effect of U0126 or NHE7 knockdown treatment on the protein expression of macrophage senescence markers (p53, p21, and p16) and MAPK-related markers (p-ERK, ERK, p-MEK, and MEK) in macrophages by WB. N = 3; $^{*}P < 0.05$, $^{**}P < 0.01$. (E-F) To detect the effect of U0126 or NHE7 knockdown treatment on Ishikawa cells proliferation (E) and

migration (F) by Transwell and MTT assays. Scale bar: 100 μm; N = 3; *P < 0.05, **P < 0.01.

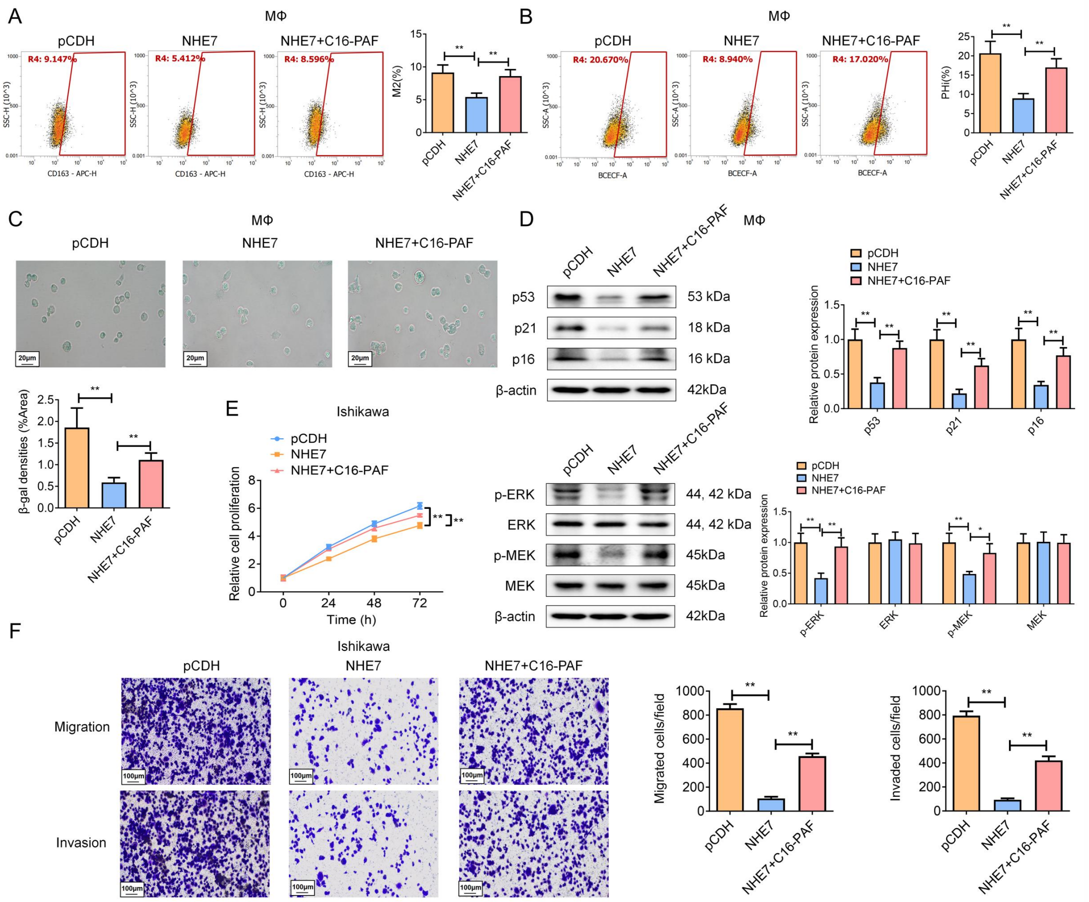

Figure S9. MAPK activation counteracts the regulatory effects induced by NHE7 overexpression. (A) To detect the expression of CD163 in macrophages treated with MAPK activation (C16-PAF) or NHE7 overexpression by flow cytometry. N = 3; **P < 0.01. (B) Flow cytometry was used to detect the intracellular pH in NHE7 overexpression macrophages treated with the MEK activation C16-PAF. (C) To detect the effect of MAPK activation (C16-PAF) or NHE7 overexpression treatment on macrophage senescence by β-galactosidase staining. N = 3; **P < 0.01. (D) To detect the effect of C16-PAF or NHE7 overexpression treatment on the protein expression of

macrophage senescence markers (p53, p21, and p16) and MAPK-related markers (p-ERK, ERK, p-MEK, and MEK) in macrophages by WB. N = 3; *P < 0.05, **P < 0.01. (E-F) To detect the effect of C16-PAF or NHE7 overexpression treatment on Ishikawa cells proliferation (E) and migration (F) by Transwell and MTT assays. Scale bar: 100 μm; N = 3; *P < 0.05, **P < 0.01.

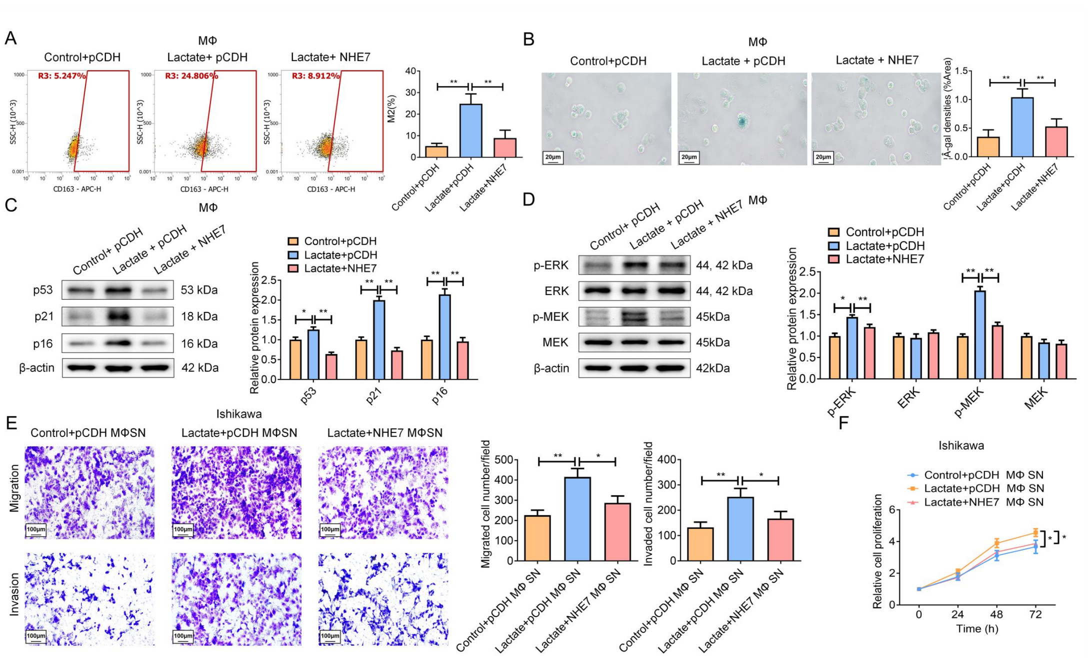

Figure S10. The pivotal role of NHE7 in governing lactate-driven macrophage plasticity and EC malignancy. (A) To detect the expression of CD163 in macrophages treated with lactate or NHE7 overexpression by flow cytometry. N = 3; **P < 0.01. (B) To detect the effect of lactate or NHE7 overexpression treatment on macrophage senescence by β-galactosidase staining. N = 3; **P < 0.01. (C) To detect the effect of lactate or NHE7 overexpression treatment on the protein expression of macrophage senescence markers (p53, p21, and p16) in macrophages by WB. N = 3; *P < 0.05, **P < 0.01. (D) To detect the effect of lactate or NHE7 overexpression

treatment on the protein expression of MAPK-related markers (p-ERK, ERK, p-MEK, and MEK) in macrophages by WB. N = 3; *$P < 0.05$, **$P < 0.01$. (E-F) To detect the effect of lactate or NHE7 overexpression treatment on macrophage migration (E) and proliferation (F) by Transwell and MTT assays. Scale bar: 100 μm; N = 3; *$P < 0.05$, **$P < 0.01$.

Supplementary Table：

Table S1. The clinical relevance of NHE7 expression in EC.

Character	Levels	Low expression of *NHE7*	High expression of *NHE7*	*p*
Age	≤65	168 (30.7%)	140 (25.6%)	0.018
	>65	106 (19.4%)	133 (24.3%)	
Stage	I	192 (34.9%)	150 (27.3%)	0.000
	II	22 (4.0%)	30 (5.5%)	
	III	51 (9.3%)	76 (13.8%)	
	IV	10 (1.8%)	19 (3.5%)	

Supplementary Material 1. PCR original data

HBA2	SYBR			
Well	SampleName	Ct		
	Normoxia	21.68268662	Hypoxia	21.46240301
	Normoxia	21.0499183	Hypoxia	21.91011144
	Normoxia	21.01278544	Hypoxia	21.52594311
	avg	21.24846345	avg	21.63281919
	sd	0.376506349	sd	0.242234559

LCTL	SYBR			
Well	SampleName	Ct		
	Normoxia	22.64788618	Hypoxia	22.73921434
	Normoxia	22.30508382	Hypoxia	22.98487023
	Normoxia	22.01699912	Hypoxia	22.38823761
	avg	22.32332304	avg	22.7041074
	sd	0.315838761	sd	0.299861627

NHE7	SYBR			
Well	SampleName	Ct		
	Normoxia	22.61474964	Hypoxia	23.81737674
	Normoxia	22.11543074	Hypoxia	23.89922278
	Normoxia	22.80917794	Hypoxia	23.99341669
	avg	22.51311944	avg	23.90333874
	sd	0.357865643	sd	0.088092121

DNMT1	SYBR			
Well	SampleName	Ct		
	Normoxia	23.1788679	Hypoxia	23.03924185
	Normoxia	23.18123631	Hypoxia	23.64278214
	Normoxia	23.35975533	Hypoxia	23.21750145
	avg	23.23995318	avg	23.29984181
	sd	0.103758462	sd	0.310080908

TEKT1	SYBR			
Well	SampleName	Ct		
	Normoxia	23.05110407	Hypoxia	23.75406145
	Normoxia	23.18684885	Hypoxia	24.79421282
	Normoxia	23.48409804	Hypoxia	23.59166376
	avg	23.24068365	avg	24.04664601
	sd	0.221460117	sd	0.652483987

ACTIN	SYBR			
Well	SampleName	Ct		
	Normoxia	14.755	Hypoxia	15.068
	Normoxia	14.322	Hypoxia	15.532
	Normoxia	14.647	Hypoxia	14.946
	avg	14.57482624	avg	15.18205477
	sd	0.225668014	sd	0.309358249

	HBA2	Actin			
Group	Ct sample	Ct internal c	ΔCt	ΔΔCt	2-ΔΔCt
Normoxia	21.68268662	14.755	6.9	0.0	1
Hypoxia	21.46240301	15.068	6.4	-0.5	1.447123894
Normoxia	21.0499183	14.322	6.7	0.0	1
Hypoxia	21.91011144	15.532	6.4	-0.4	1.274784319
Normoxia	21.01278544	14.647	6.4	0.0	1
Hypoxia	21.52594311	14.946	6.6	0.2	0.861651731

	LCTL	Actin			
Group	Ct sample	Ct internal c	ΔCt	ΔΔCt	2-ΔΔCt
Normoxia	22.64788618	14.755	7.9	0.0	1
Hypoxia	22.73921434	15.068	7.7	-0.2	1.166007181
Normoxia	22.30508382	14.322	8.0	0.0	1
Hypoxia	22.98487023	15.532	7.5	-0.5	1.444589815
Normoxia	22.01699912	14.647	7.4	0.0	1
Hypoxia	22.38823761	14.946	7.4	0.1	0.95072233

	NHE7	Actin			
Group	Ct sample	Ct internal c	ΔCt	ΔΔCt	2-ΔΔCt
Normoxia	22.61474964	14.755	7.9	0.0	1
Hypoxia	23.81737674	15.068	8.7	0.9	0.539718011
Normoxia	22.11543074	14.322	7.8	0.0	1
Hypoxia	23.89922278	15.532	8.4	0.6	0.672056429
Normoxia	22.80917794	14.647	8.2	0.0	1
Hypoxia	23.99341669	14.946	9.0	0.9	0.541148214

	DNMT1	Actin			
Group	Ct sample	Ct internal c	ΔCt	ΔΔCt	2-ΔΔCt
Normoxia	23.1788679	14.755	8.4	0.0	1
Hypoxia	23.03924185	15.068	8.0	-0.5	1.368438768
Normoxia	23.18123631	14.322	8.9	0.0	1
Hypoxia	23.64278214	15.532	8.1	-0.7	1.680511598
Normoxia	23.35975533	14.647	8.7	0.0	1
Hypoxia	23.21750145	14.946	8.3	-0.4	1.357157651

	TEKT1	Actin			
Group	Ct sample	Ct internal c	ΔCt	ΔΔCt	2-ΔΔCt
Normoxia	23.05110407	14.755	8.3	0.0	1
Hypoxia	23.75406145	15.068	8.7	0.4	0.763101811
Normoxia	23.18684885	14.322	8.9	0.0	1
Hypoxia	24.79421282	15.532	9.3	0.4	0.759479194
Normoxia	23.48409804	14.647	8.8	0.0	1
Hypoxia	23.59166376	14.946	8.6	-0.2	1.141371709

HEC-1-A

SLC16A8	SYBR			
Well	SampleName	Ct		
	Normoxia	24.38822597	Hypoxia	24.24679167
	Normoxia	24.1138582	Hypoxia	24.23339685
	Normoxia	24.45131708	Hypoxia	24.41909935
	avg	24.31780042	avg	24.29976262
	sd	0.179414164	sd	0.103565416

ACTIN	SYBR			
Well	SampleName	Ct		
	Normoxia	15.864	Hypoxia	16.504
	Normoxia	15.471	Hypoxia	16.837
	Normoxia	15.937	Hypoxia	16.753
	avg	15.75725587	avg	16.69790247
	sd	0.250453297	sd	0.173340296

	SLC16A8	Actin			
Group	Ct sample	Ct internal c	ΔCt	ΔΔCt	2-ΔΔCt
Normoxia	24.38822597	15.864	8.5	0.0	1
Hypoxia	24.24679167	16.504	7.7	-0.8	1.71883412
Normoxia	24.1138582	15.471	8.6	0.0	1
Hypoxia	24.23339685	16.837	7.4	-1.2	2.371877981
Normoxia	24.45131708	15.937	8.5	0.0	1
Hypoxia	24.41909935	16.753	7.7	-0.8	1.800743977

Ishikawa

SLC16A8	SYBR			
Well	SampleName	Ct		
	Normoxia	22.17818084	Hypoxia	21.75046658
	Normoxia	22.51005139	Hypoxia	21.84008717
	Normoxia	22.62079957	Hypoxia	21.76233922
	avg	22.43634393	avg	21.78429765
	sd	0.230331121	sd	0.048678458

ACTIN	SYBR			
Well	SampleName	Ct		
	Normoxia	16.058	Hypoxia	16.776
	Normoxia	16.197	Hypoxia	16.326
	Normoxia	16.308	Hypoxia	16.631
	avg	16.18773378	avg	16.57748058
	sd	0.125297631	sd	0.229681222

	SLC16A8	Actin			
Group	Ct sample	Ct internal c	ΔCt	ΔΔCt	2-ΔΔCt
Normoxia	22.17818084	16.058	6.1	0.0	1
Hypoxia	21.75046658	16.776	5.0	-1.1	2.212337255
Normoxia	22.51005139	16.197	6.3	0.0	1
Hypoxia	21.84008717	16.326	5.5	-0.8	1.739171892
Normoxia	22.62079957	16.308	6.3	0.0	1
Hypoxia	21.76233922	16.631	5.1	-1.2	2.267979165

DNMT1	SYBR			
Well	SampleName	Ct		
	pcDNA3.1	22.37665558	DNMT1	16.390391
	pcDNA3.2	22.26326533	DNMT1	16.9122639
	pcDNA3.3	22.70344744	DNMT1	16.4807217
	avg	22.44778945	avg	16.5944589
	sd	0.228549981	sd	0.27890852

NHE7	SYBR			
Well	SampleName	Ct		
	pcDNA3.1	21.84063058	DNMT1	23.2223917
	pcDNA3.2	21.51328347	DNMT1	23.6746392
	pcDNA3.3	21.65873006	DNMT1	23.1738092
	avg	21.67088137	avg	23.3569467
	sd	0.164011508	sd	0.27620006

ACTIN	SYBR			
Well	SampleName	Ct		
	pcDNA3.1	15.669	DNMT1	16.321
	pcDNA3.2	15.221	DNMT1	16.544
	pcDNA3.3	15.671	DNMT1	16.003
	avg	15.52044812	avg	16.2893331
	sd	0.258900888	sd	0.27222491

	DNMT1	Actin			
组别	Ct sample	Ct internal c	ΔCt	ΔΔCt	2-ΔΔCt
pcDNA3.1	22.37665558	15.669	6.7	0.0	1
DNMT1	16.39039098	16.321	0.1	-6.6	99.63022178
pcDNA3.1	22.26326533	15.221	7.0	0.0	1
DNMT1	16.91226395	16.544	0.4	-6.7	102.0929705
pcDNA3.1	22.70344744	15.671	7.0	0.0	1
DNMT1	16.48072167	16.003	0.5	-6.6	93.98739882

	NHE7	Actin			
组别	Ct sample	Ct internal c	ΔCt	ΔΔCt	2-ΔΔCt
pcDNA3.1	21.84063058	15.669	6.2	0.0	1
DNMT1	23.22239171	16.321	6.9	0.7	0.603106965
pcDNA3.1	21.51328347	15.221	6.3	0.0	1
DNMT1	23.67463924	16.544	7.1	0.8	0.559179186
pcDNA3.1	21.65873006	15.671	6.0	0.0	1
DNMT1	23.17380917	16.003	7.2	1.2	0.440309706

NHE7	SYBR	
Well	SampleName	Ct
	Control	22.6952186654
	Control	22.4613943265
	Control	22.1505446251
	avg	22.43571921
	sd	0.273243228

ACTIN	SYBR	
Well	SampleName	Ct
	Control	15.5755151387
	Control	15.7216436019
	Control	15.1530691674
	avg	15.4834093
	sd	0.295265704

	NHE7	Actin
group	Ct sample	Ct internal c
Control	22.69521867	15.57551514
lactate	23.38232095	14.65732904
Lactate+5-aza	22.89209553	15.67779784
Control	22.46139433	15.72164360
lactate	23.65334871	14.92428635
Lactate+5-aza	22.29108997	15.24302698
Control	22.15054463	15.15306917
lactate	23.03228808	14.50111581
Lactate+5-aza	22.82028285	15.94806899

lactate	23.3823209518
lactate	23.6533487063
lactate	23.0322880814
avg	23.35598591
sd	0.311366706

lactate	14.6573290446
lactate	14.9242863506
lactate	14.5011158085
avg	14.69424373
sd	0.213986793

ΔCt	ΔΔCt	2-ΔΔCt
7.1	0.0	1
8.7	1.6	0.328669986
7.2	0.1	0.936535663
6.7	0.0	1
8.7	2.0	0.251859031
7.0	0.3	0.807585963
7.0	0.0	1
8.5	1.5	0.345391189
6.9	-0.1	1.090705483

Lactate+5-aza	22.89209553
Lactate+5-aza	22.29108997
Lactate+5-aza	22.82028285
avg	22.66782278
sd	0.328230064

Lactate+5-aza	15.67779784
Lactate+5-aza	15.24302698
Lactate+5-aza	15.94806899
avg	15.6229646
sd	0.355705033

Supplementary Material 2. Uncropped blot images from Westernblotting experiments

Fig 2C

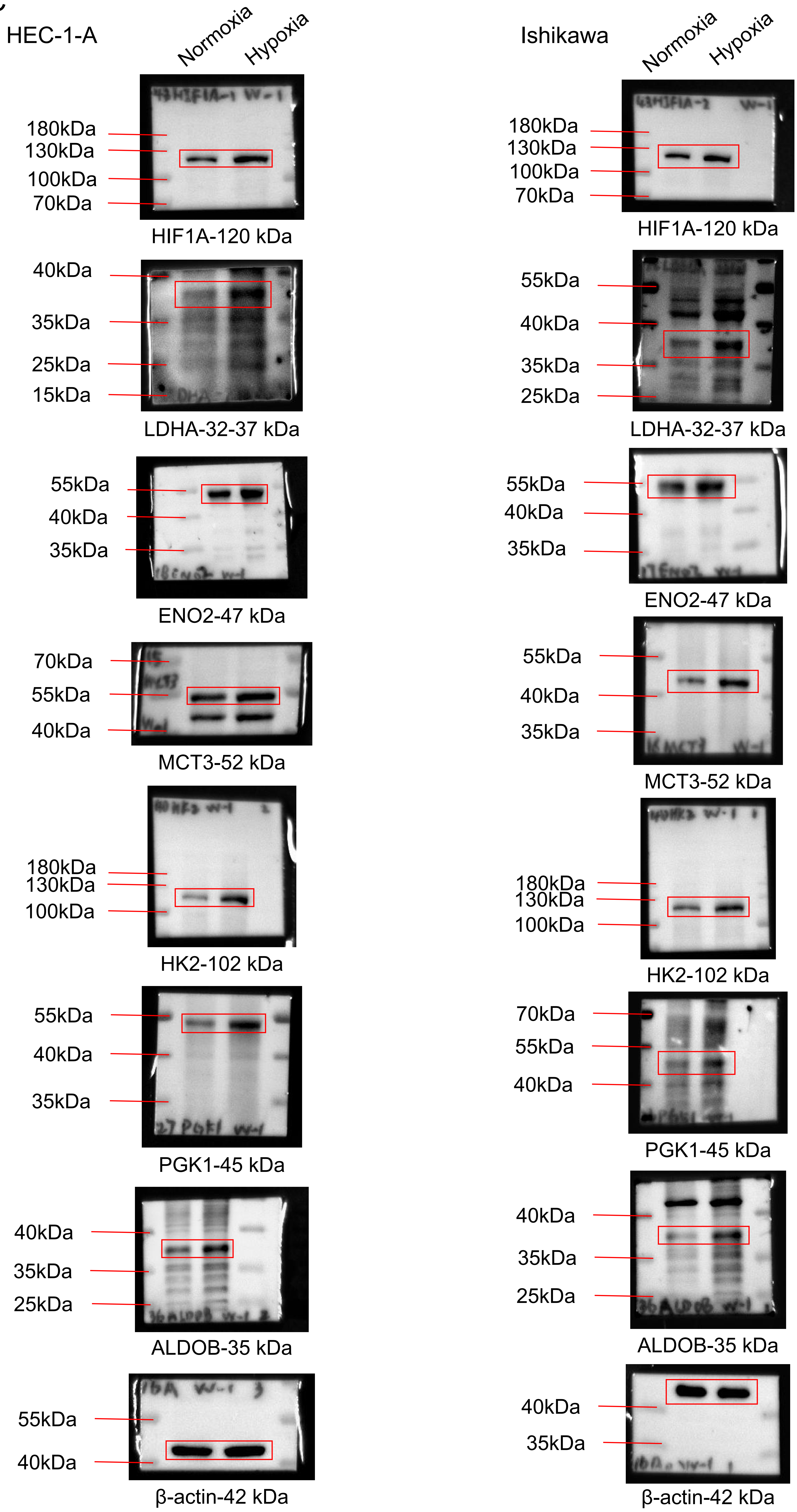

Fig S3A

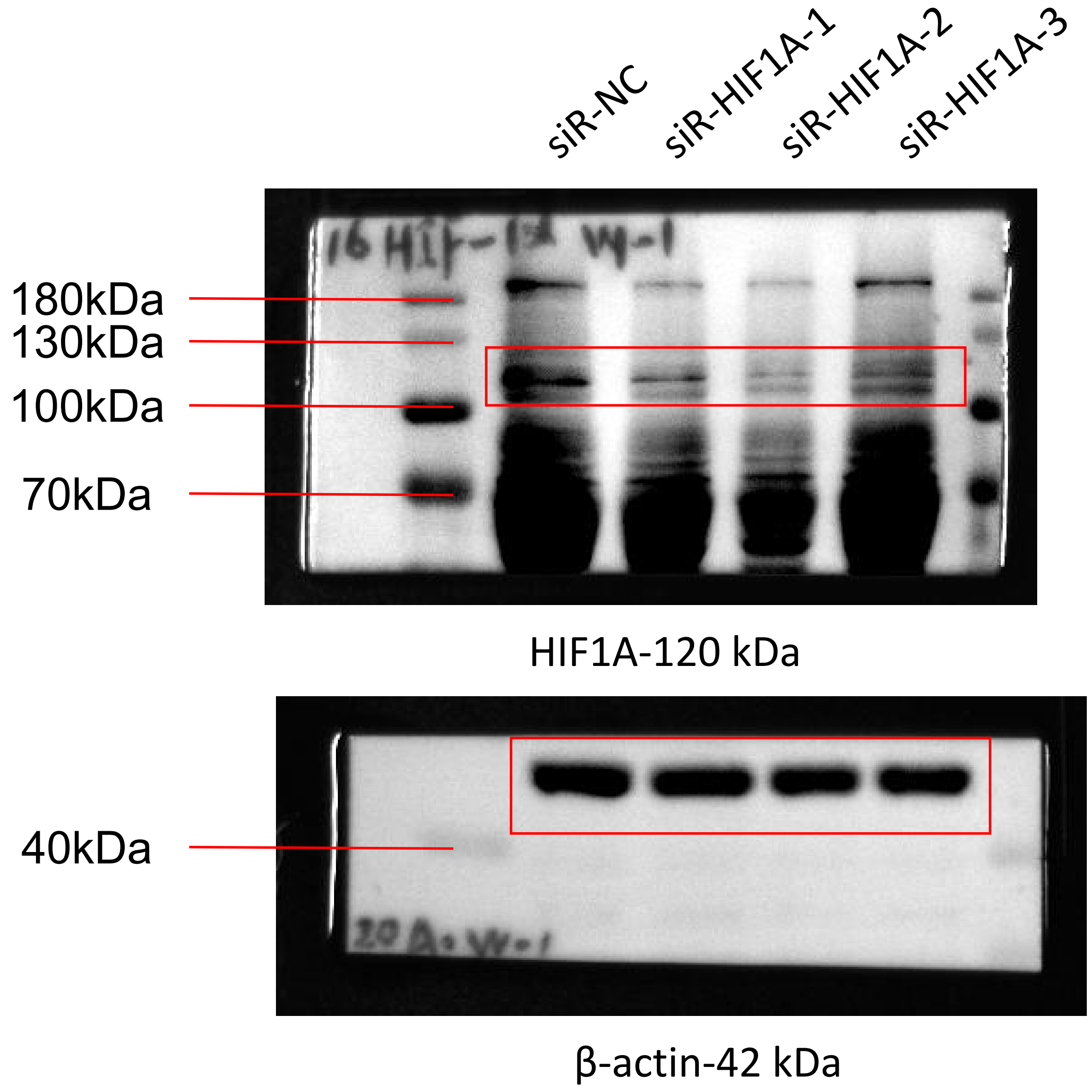

Fig S3E

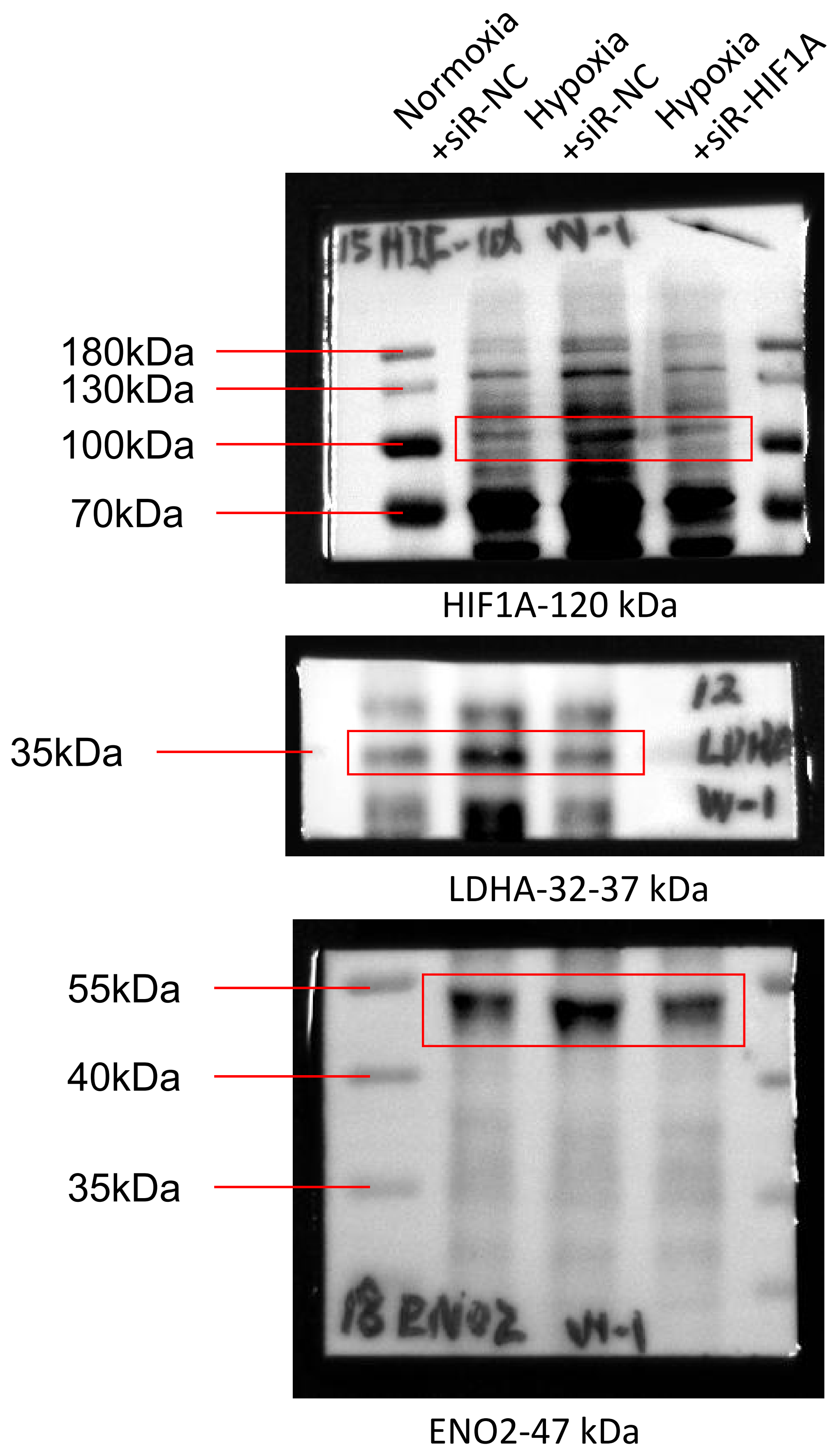

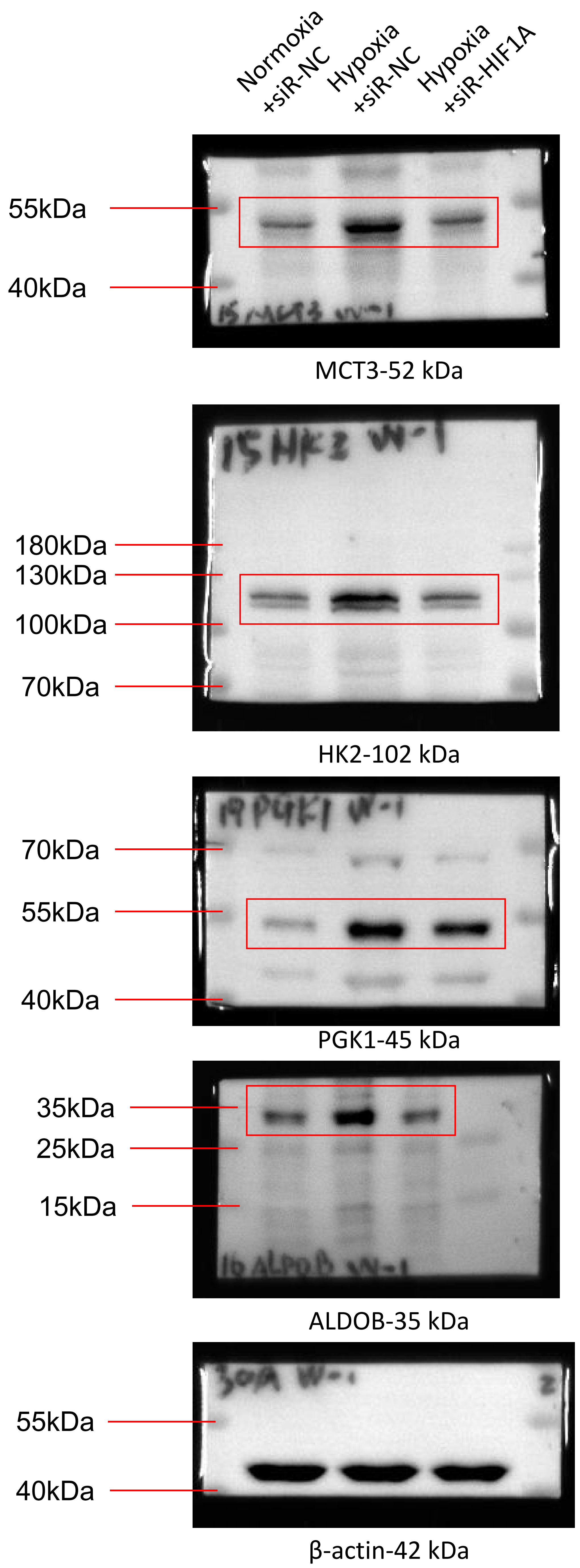

Fig 4D

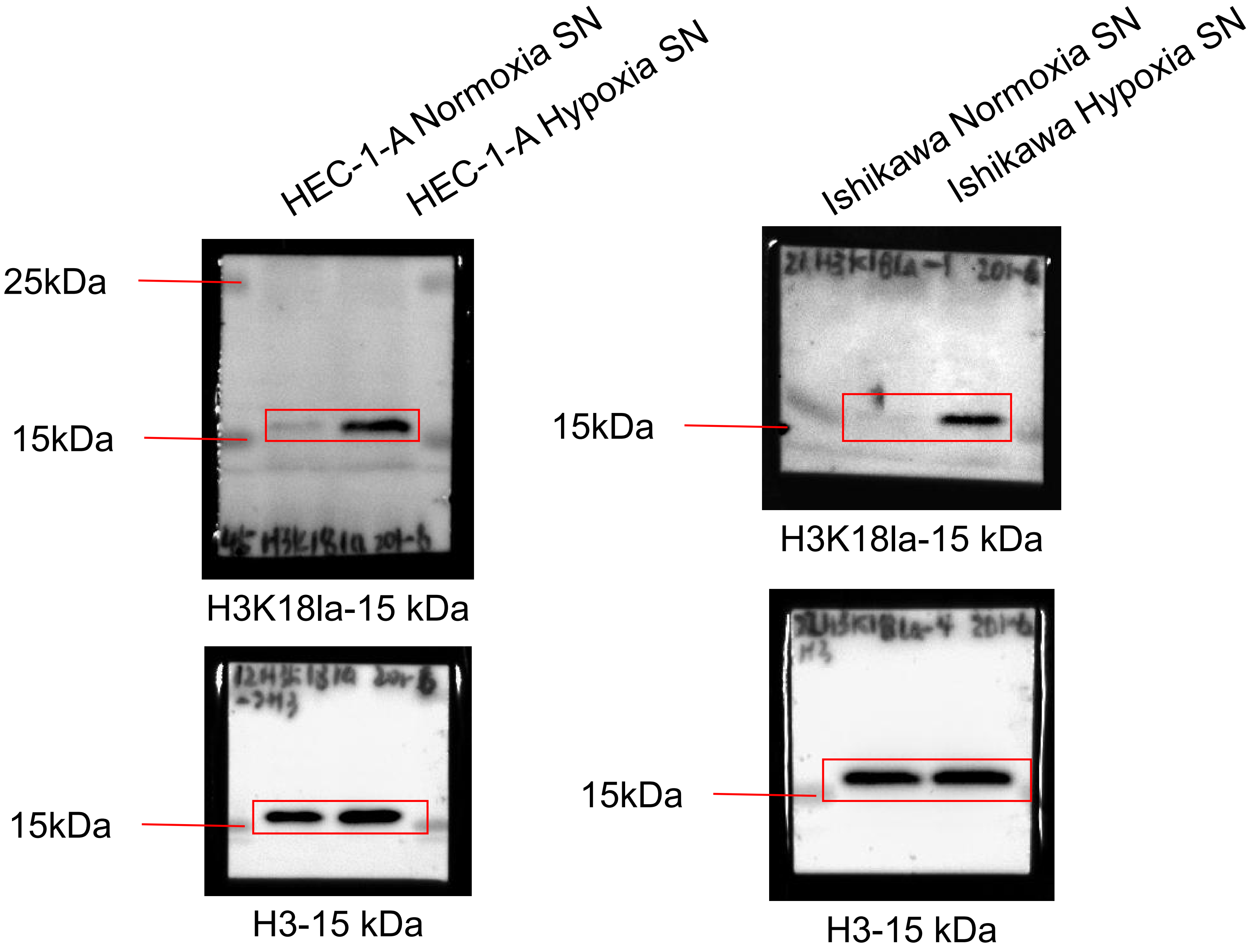

Fig 4F

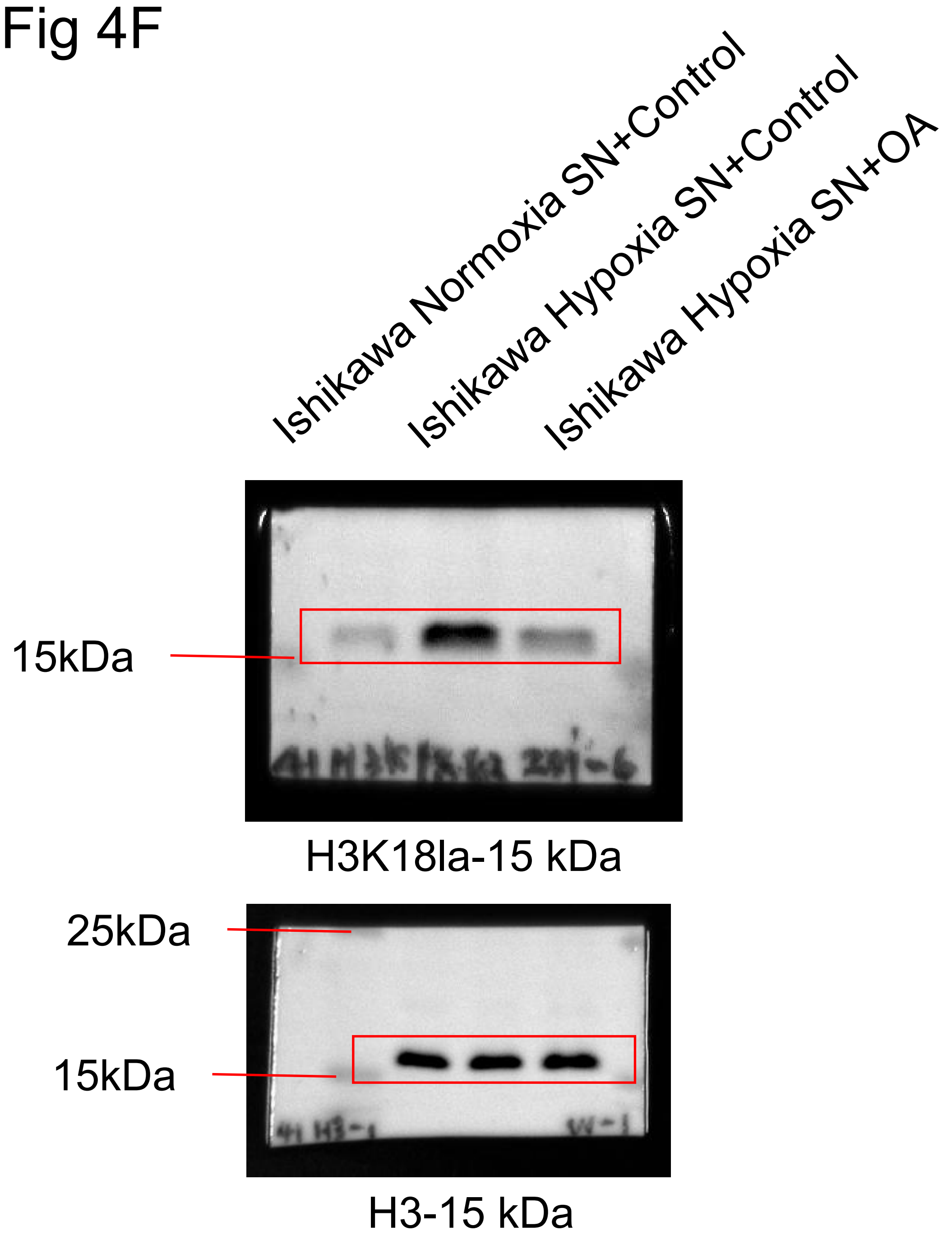

Fig 4H

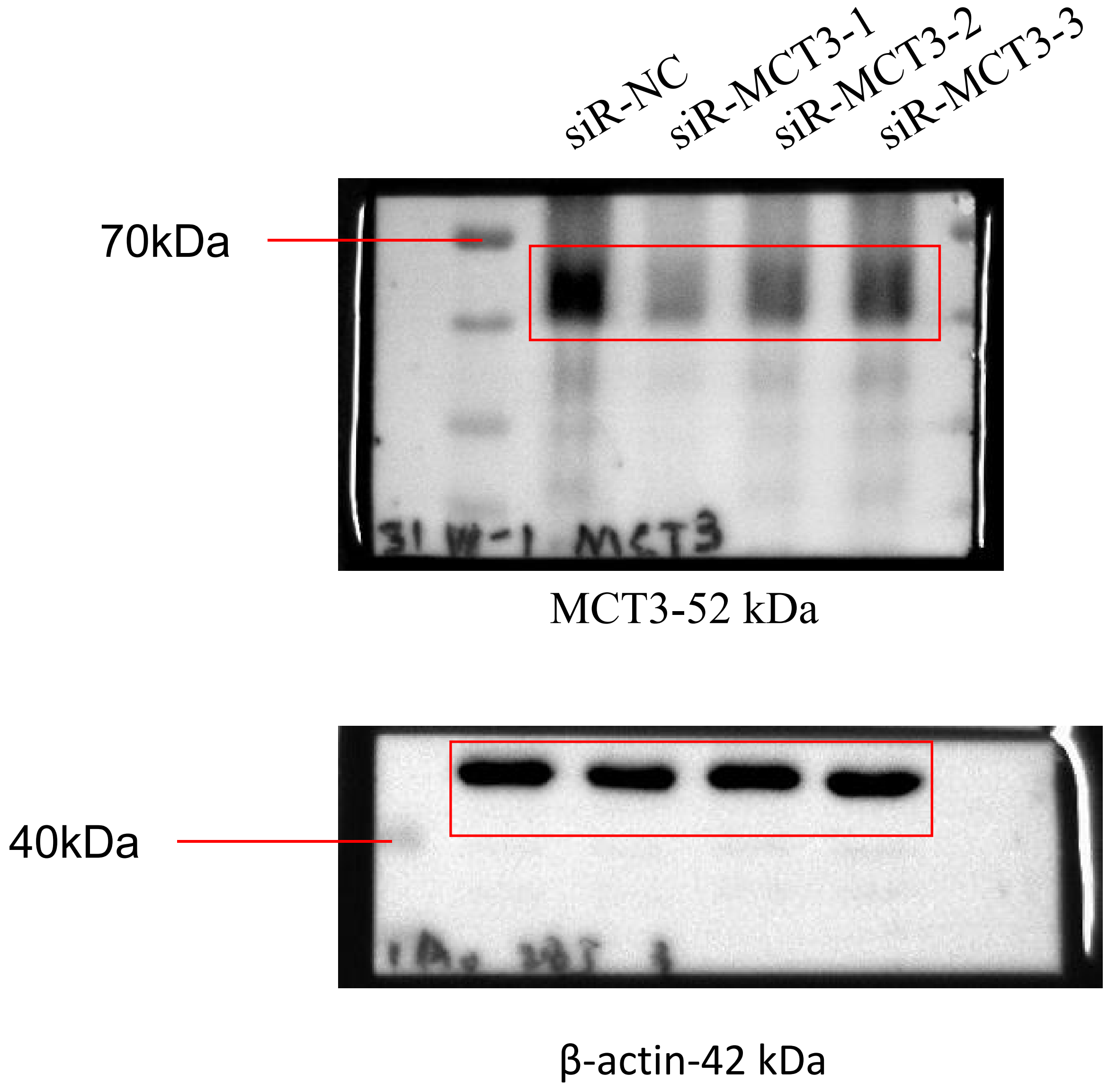

Fig 5G

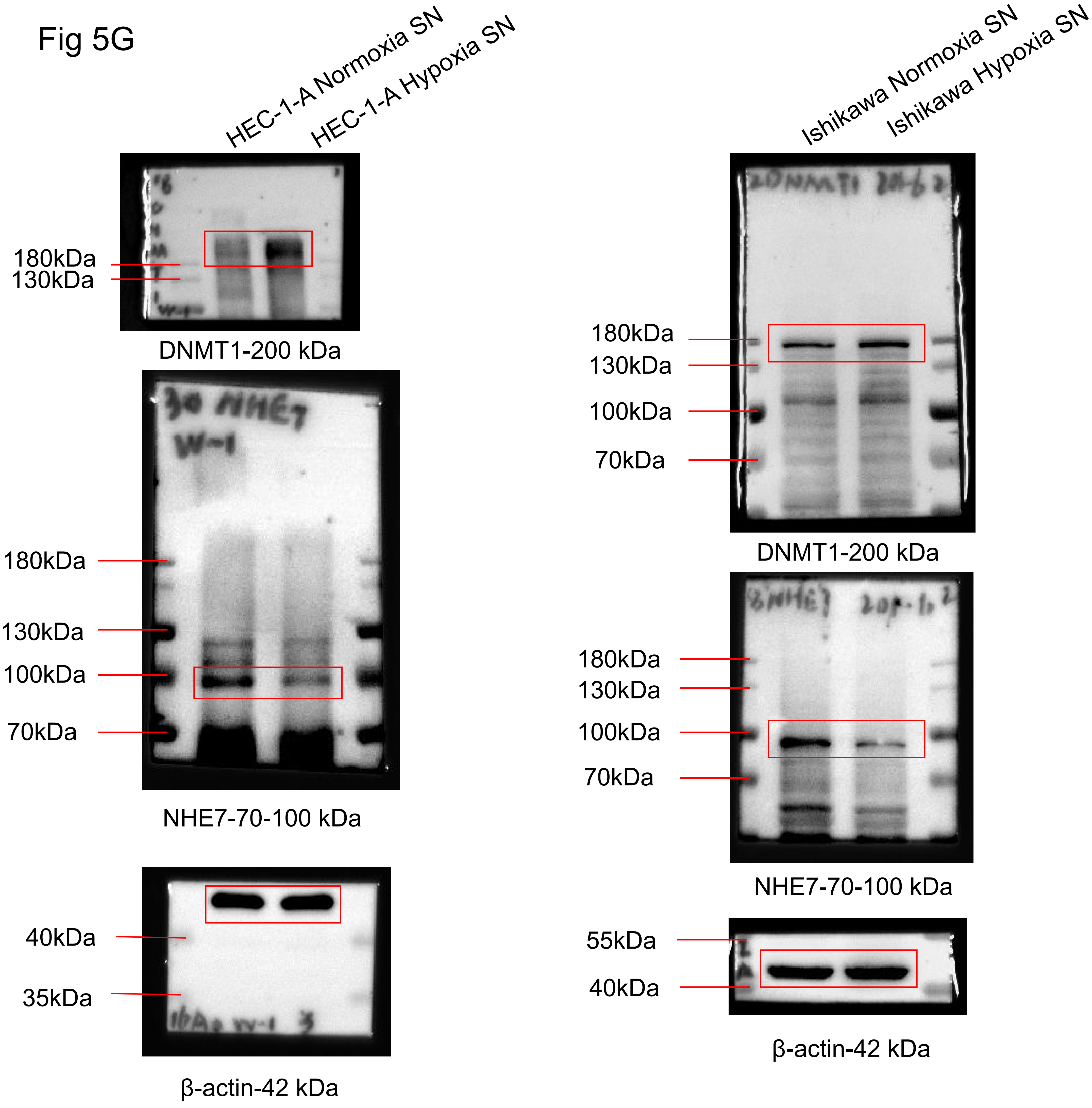
HEC-1-A Normoxia SN
HEC-1-A Hypoxia SN
Ishikawa Normoxia SN
Ishikawa Hypoxia SN
180kDa
130kDa
DNMT1-200 kDa
180kDa
130kDa
100kDa
70kDa
NHE7-70-100 kDa
40kDa
35kDa
β-actin-42 kDa
180kDa
130kDa
100kDa
70kDa
DNMT1-200 kDa
180kDa
130kDa
100kDa
70kDa
NHE7-70-100 kDa
55kDa
40kDa
β-actin-42 kDa

Fig 7

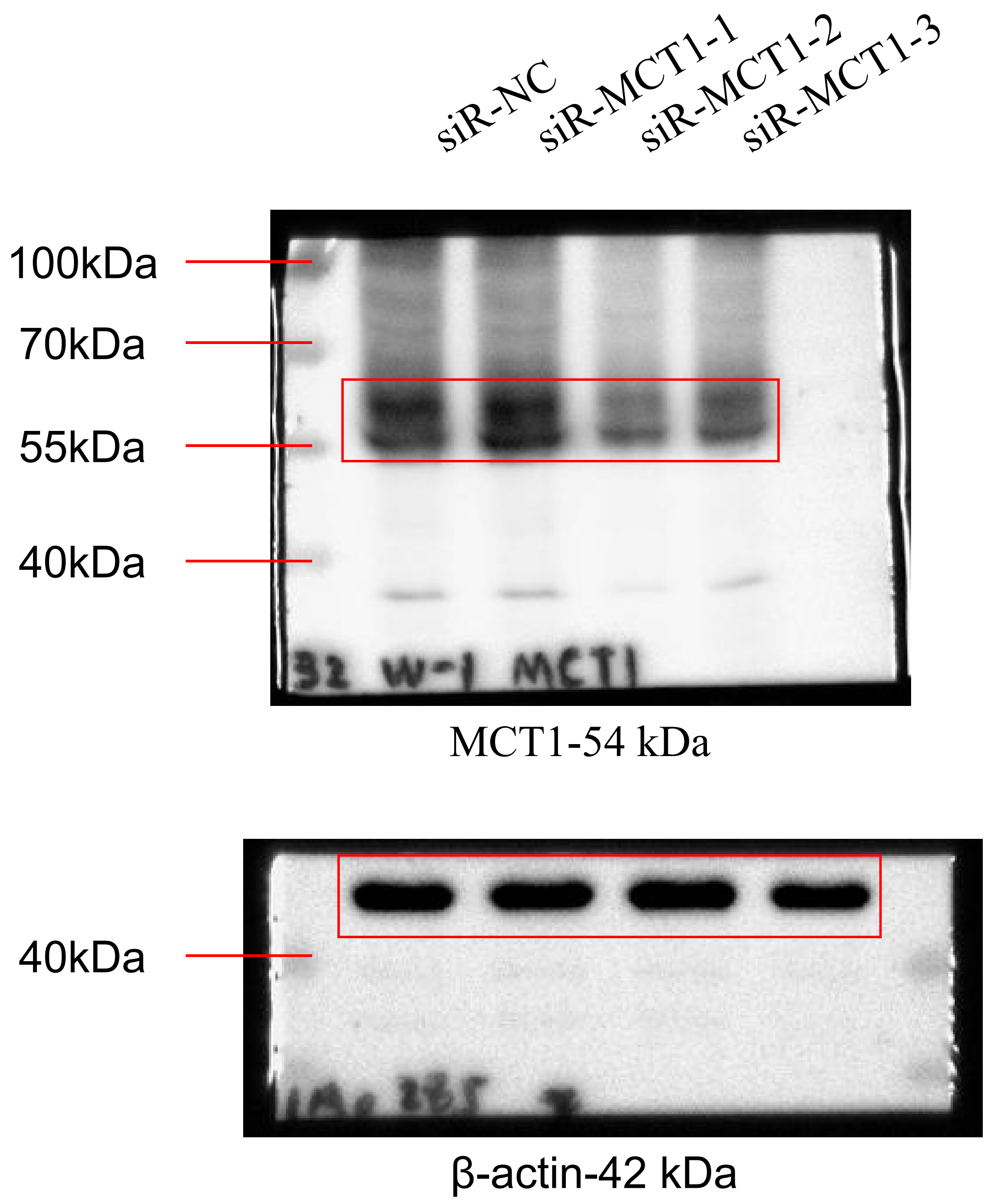
siR-NC
siR-MCT1-1
siR-MCT1-2
siR-MCT1-3
100kDa
70kDa
55kDa
40kDa
MCT1-54 kDa
40kDa
β-actin-42 kDa

Fig S7

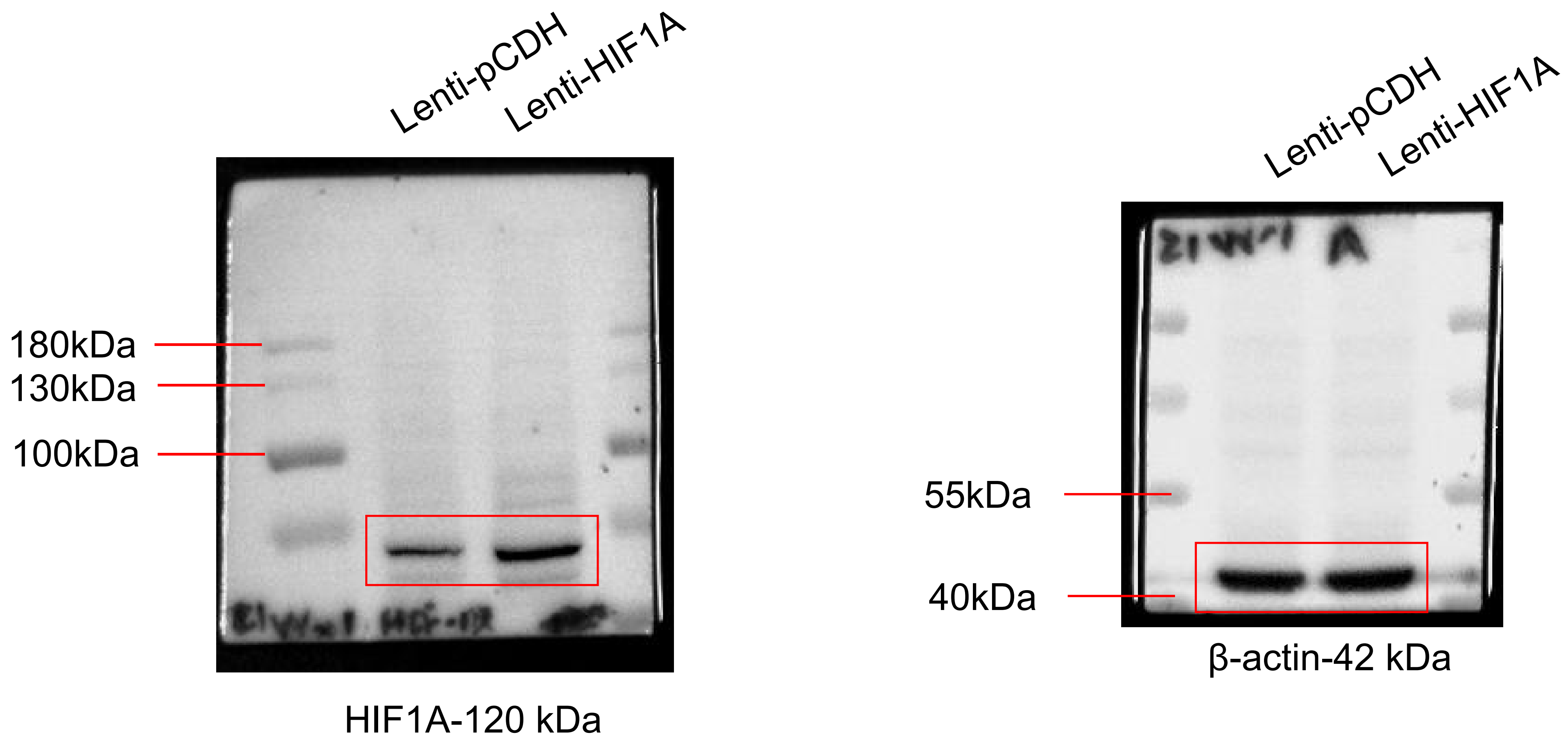
Lenti-pCDH
Lenti-HIF1A
180kDa
130kDa
100kDa
HIF1A-120 kDa
Lenti-pCDH
Lenti-HIF1A
55kDa
40kDa
β-actin-42 kDa

Fig 8A

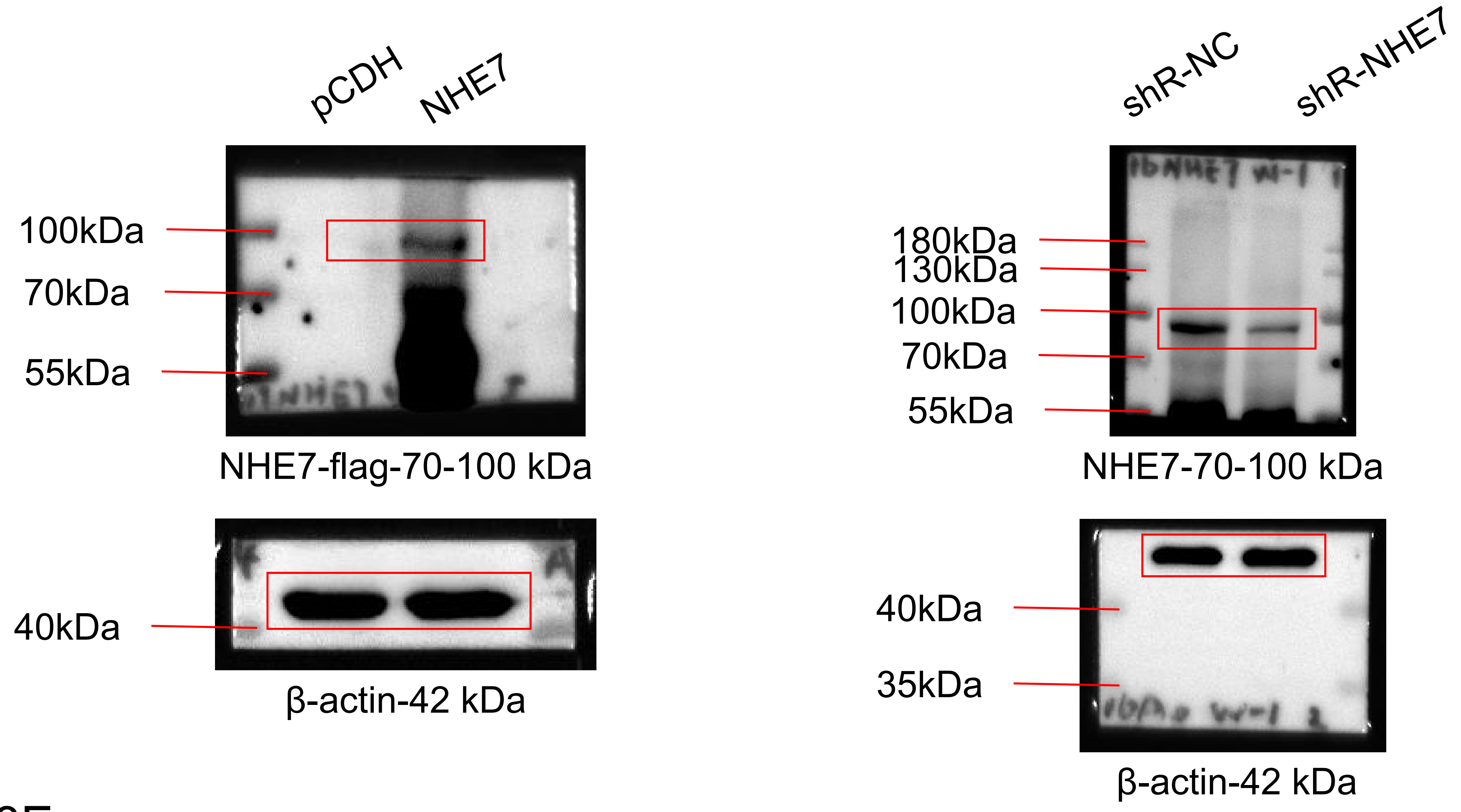

pCDH
NHE7
100kDa
70kDa
55kDa
NHE7-flag-70-100 kDa
40kDa
β-actin-42 kDa
shR-NC
shR-NHE7
180kDa
130kDa
100kDa
70kDa
55kDa
NHE7-70-100 kDa
40kDa
35kDa
β-actin-42 kDa

Fig 8E

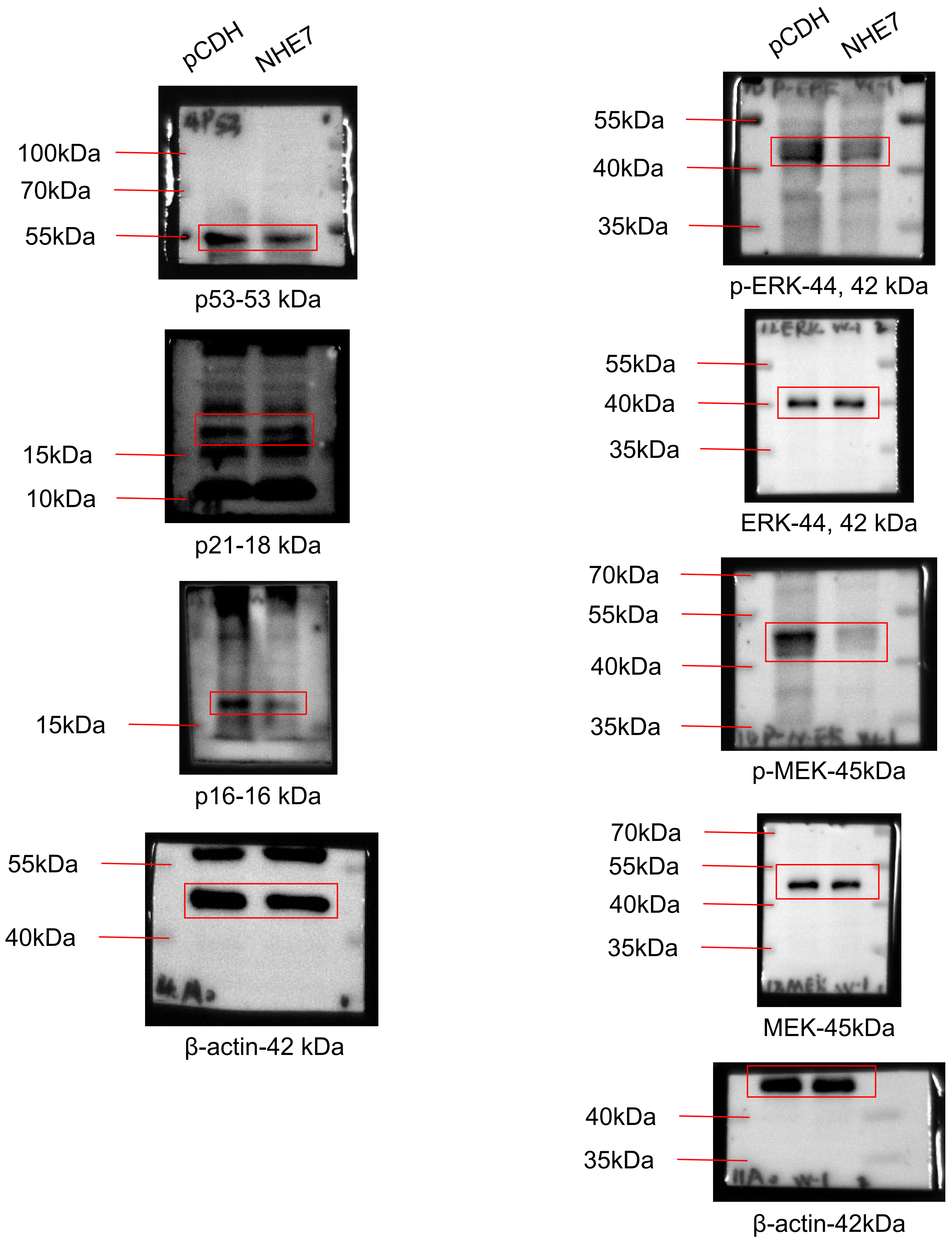

pCDH
NHE7
100kDa
70kDa
55kDa
p53-53 kDa
15kDa
10kDa
p21-18 kDa
15kDa
p16-16 kDa
55kDa
40kDa
β-actin-42 kDa
pCDH
NHE7
55kDa
40kDa
35kDa
p-ERK-44, 42 kDa
55kDa
40kDa
35kDa
ERK-44, 42 kDa
70kDa
55kDa
40kDa
35kDa
p-MEK-45kDa
70kDa
55kDa
40kDa
35kDa
MEK-45kDa
40kDa
35kDa
β-actin-42kDa

Fig 8E

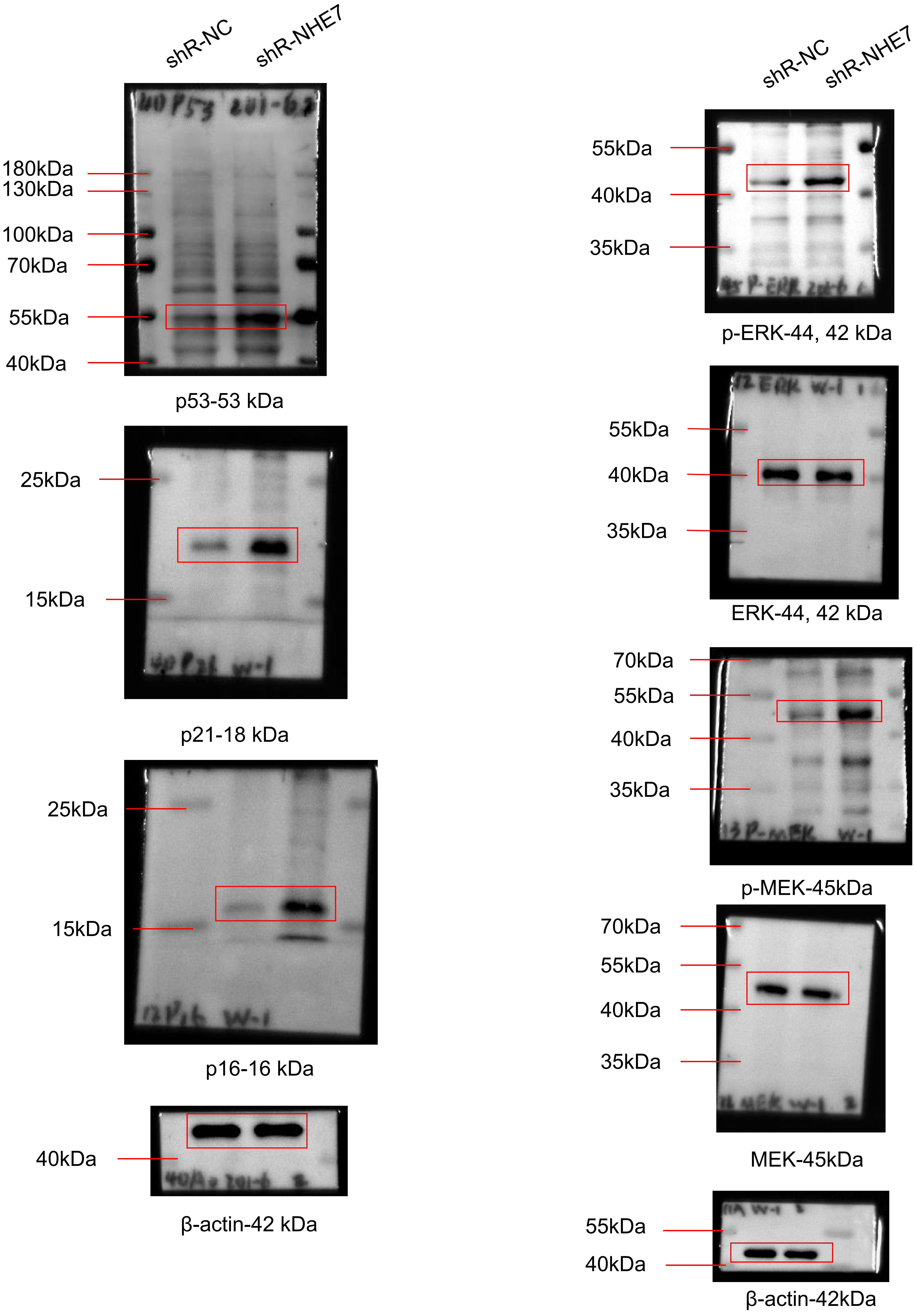
shR-NC
shR-NHE7
180kDa
130kDa
100kDa
70kDa
55kDa
40kDa
p53-53 kDa
25kDa
15kDa
p21-18 kDa
25kDa
15kDa
p16-16 kDa
40kDa
β-actin-42 kDa
shR-NC
shR-NHE7
55kDa
40kDa
35kDa
p-ERK-44, 42 kDa
55kDa
40kDa
35kDa
ERK-44, 42 kDa
70kDa
55kDa
40kDa
35kDa
p-MEK-45kDa
70kDa
55kDa
40kDa
35kDa
MEK-45kDa
55kDa
40kDa
β-actin-42kDa

Fig S8D

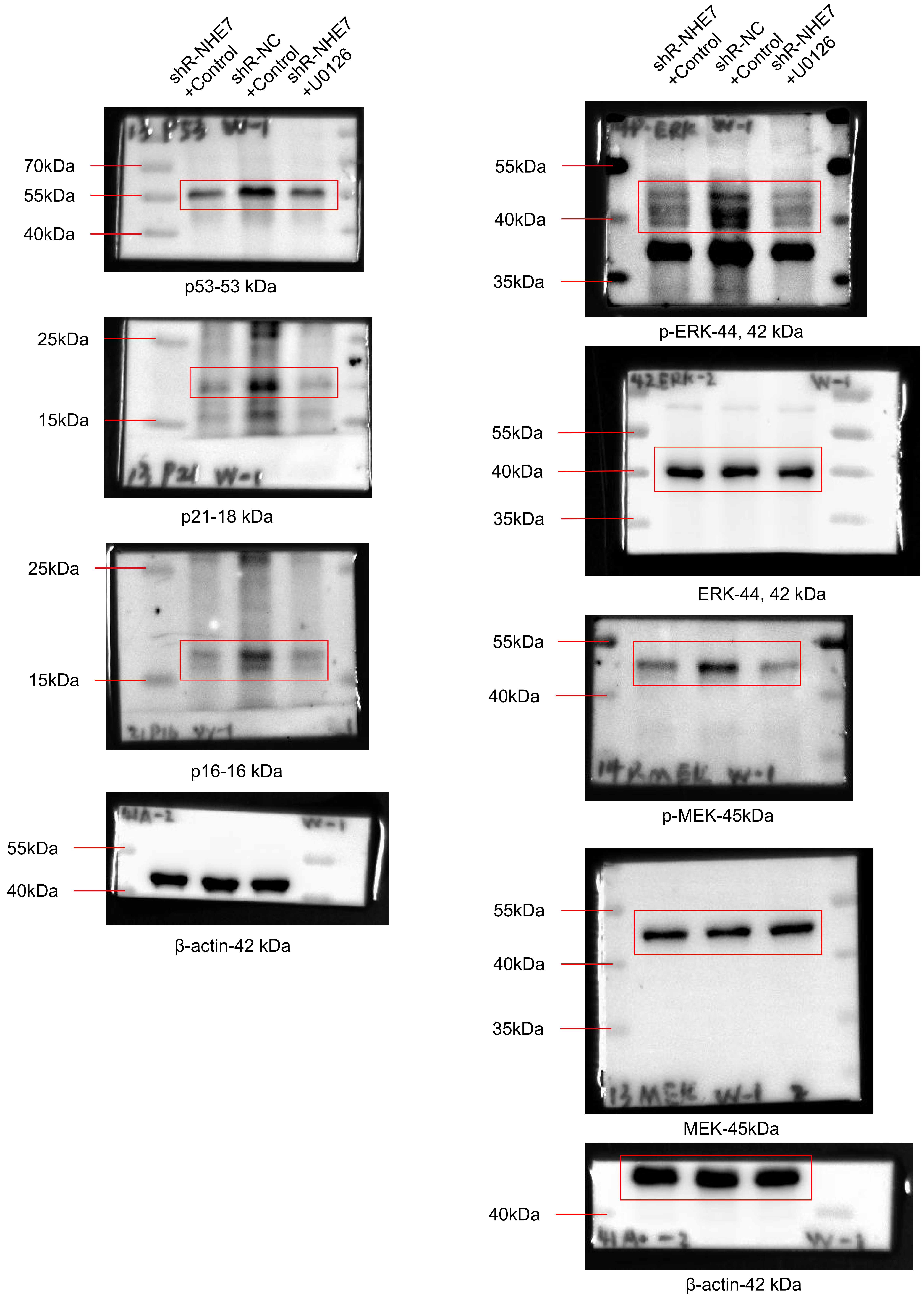
shR-NHE7
+Control
shR-NC
+Control
shR-NHE7
+U0126
70kDa
55kDa
40kDa
p53-53 kDa
25kDa
15kDa
p21-18 kDa
25kDa
15kDa
p16-16 kDa
55kDa
40kDa
β-actin-42 kDa
shR-NHE7
+Control
shR-NC
+Control
shR-NHE7
+U0126
55kDa
40kDa
35kDa
p-ERK-44, 42 kDa
55kDa
40kDa
35kDa
ERK-44, 42 kDa
55kDa
40kDa
p-MEK-45kDa
55kDa
40kDa
35kDa
MEK-45kDa
40kDa
β-actin-42 kDa

Fig 9A

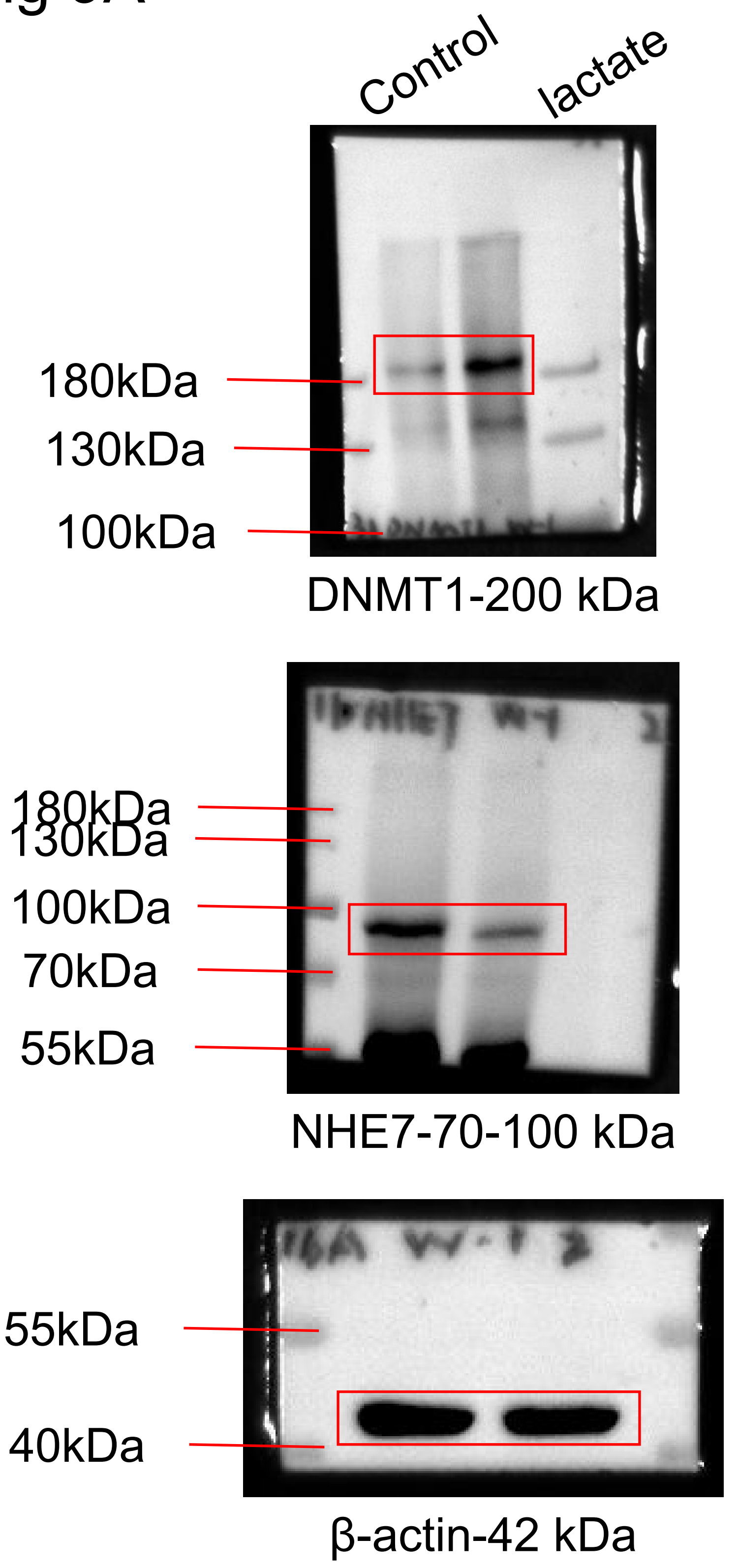

Fig 9B

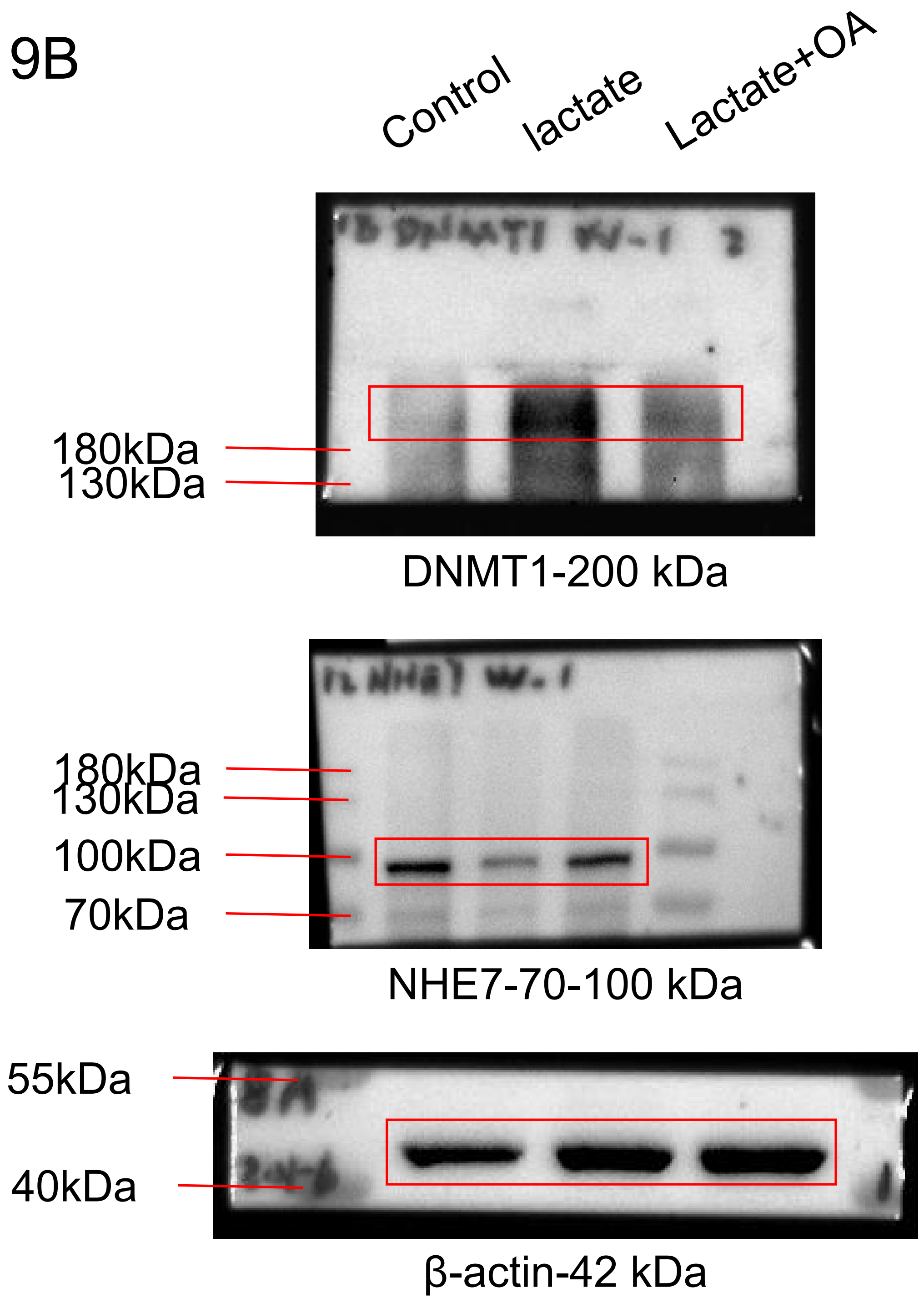

Fig 9D

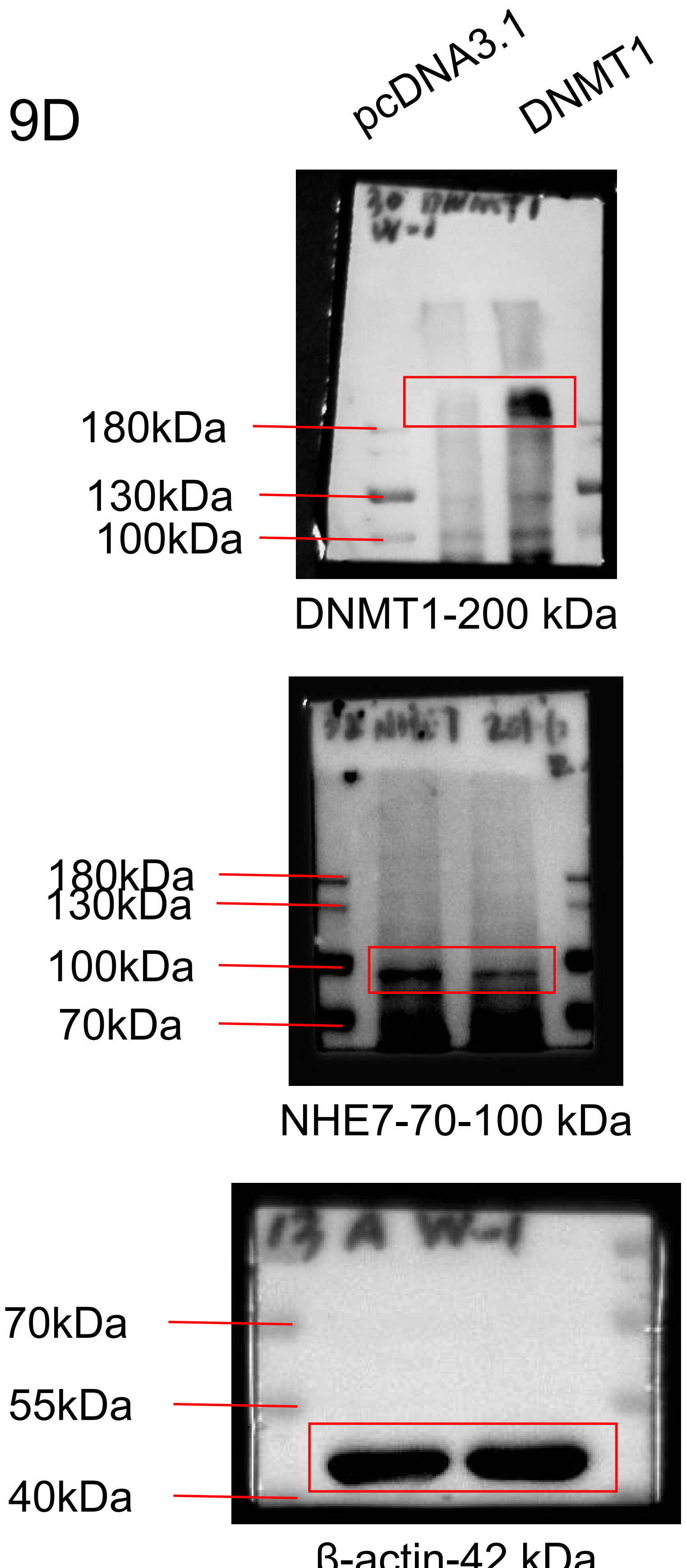

Fig 9H

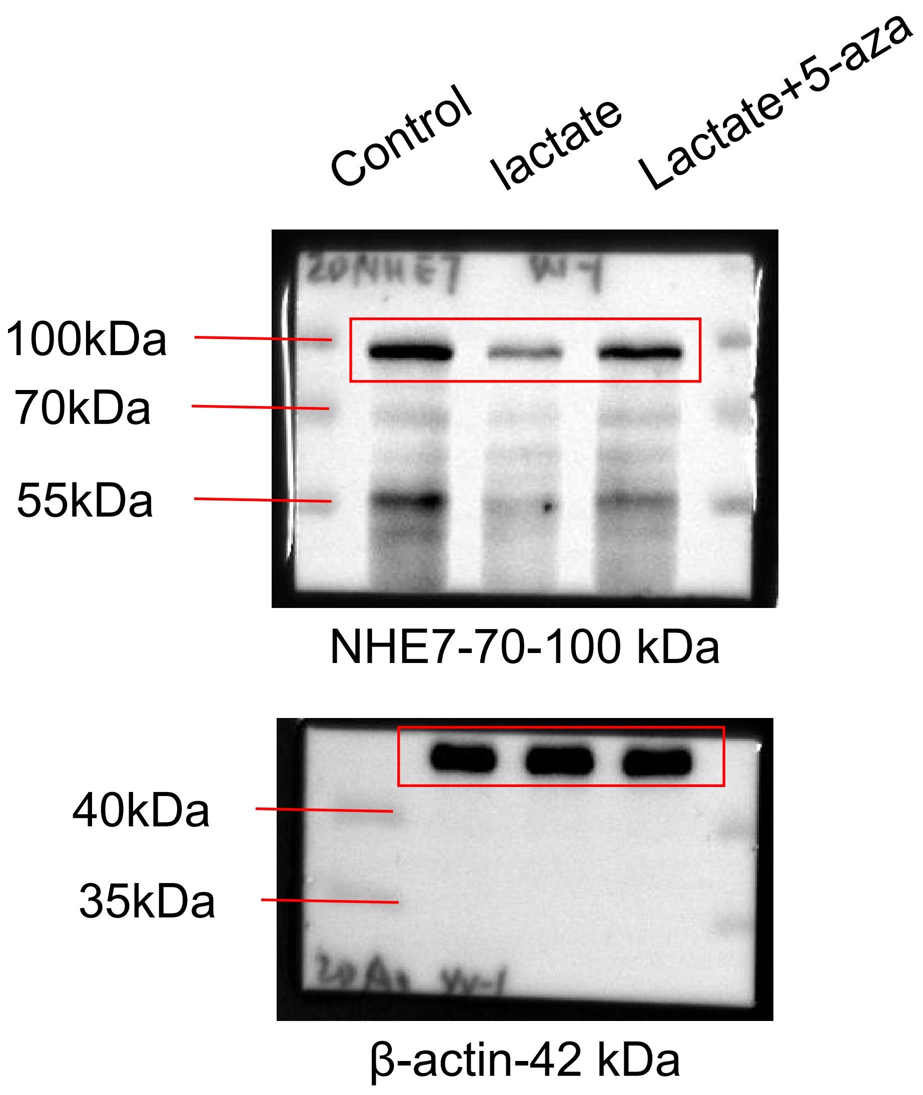

Fig S9D

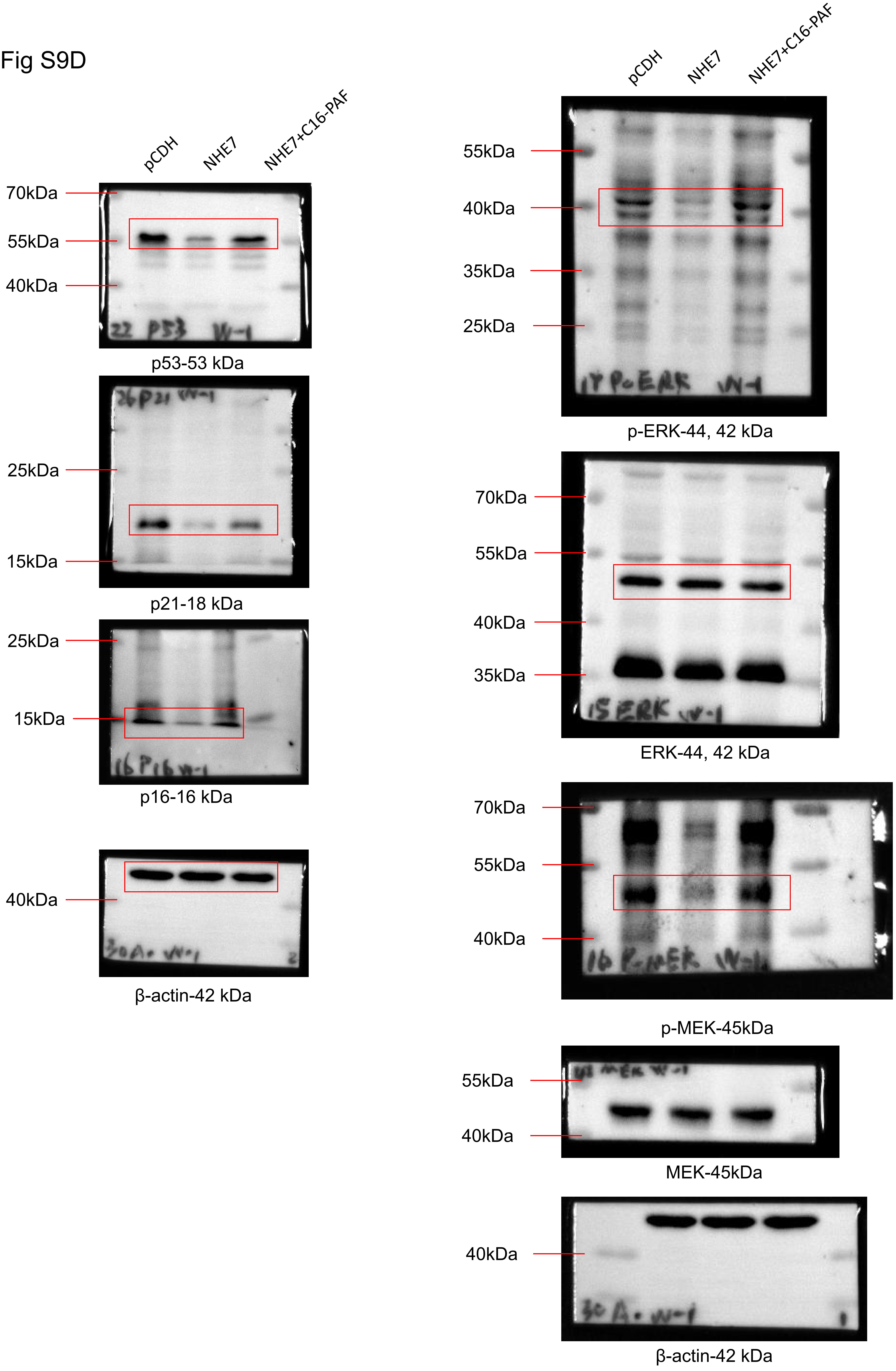
pCDH
NHE7
NHE7+C16-PAF
70kDa
55kDa
40kDa
p53-53 kDa
25kDa
15kDa
p21-18 kDa
25kDa
15kDa
p16-16 kDa
40kDa
β-actin-42 kDa
pCDH
NHE7
NHE7+C16-PAF
55kDa
40kDa
35kDa
25kDa
p-ERK-44, 42 kDa
70kDa
55kDa
40kDa
35kDa
ERK-44, 42 kDa
70kDa
55kDa
40kDa
p-MEK-45kDa
55kDa
40kDa
MEK-45kDa
40kDa
β-actin-42 kDa

Fig 10A

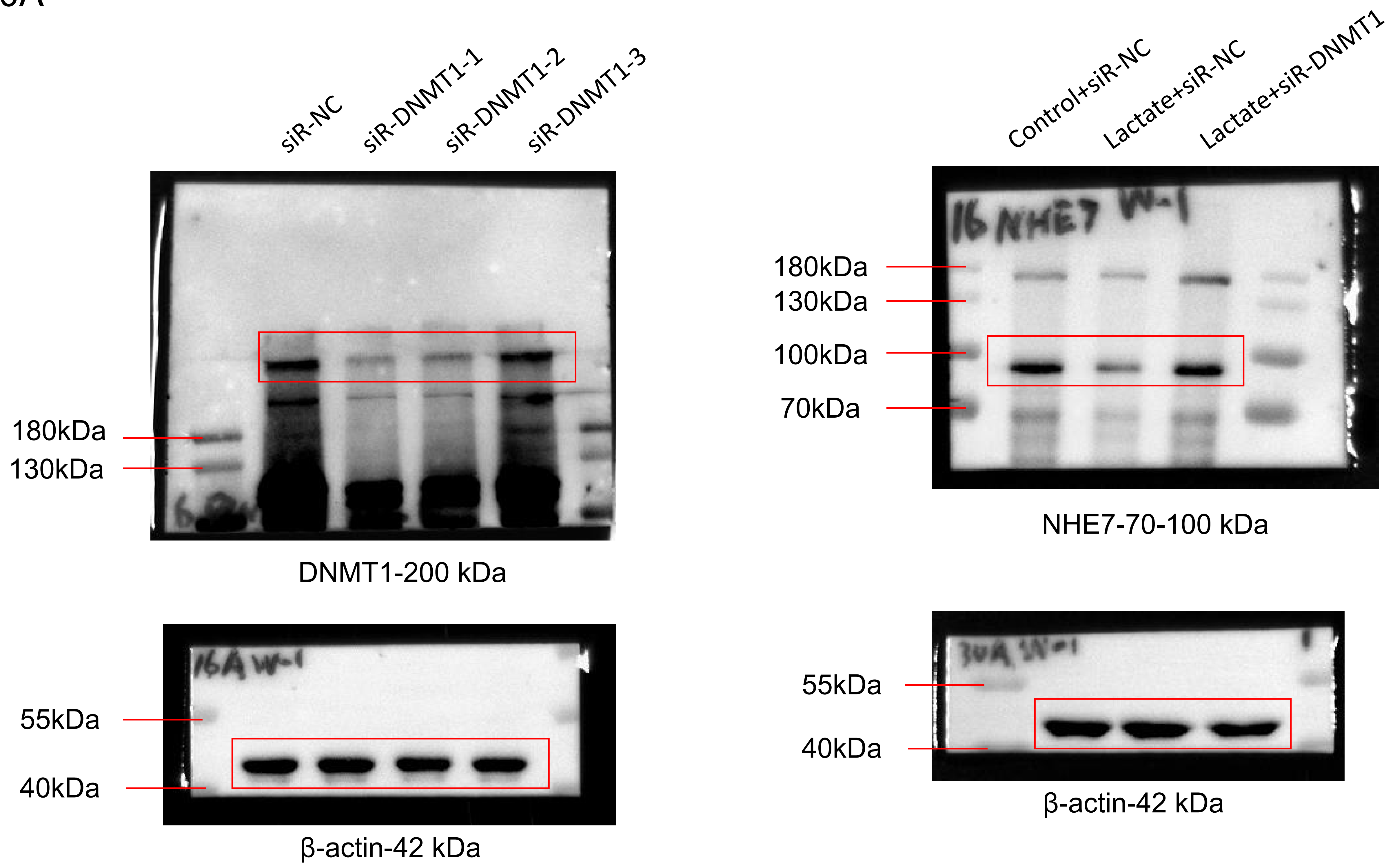
siR-NC
siR-DNMT1-1
siR-DNMT1-2
siR-DNMT1-3
180kDa
130kDa
DNMT1-200 kDa
55kDa
40kDa
β-actin-42 kDa
Control+siR-NC
Lactate+siR-NC
Lactate+siR-DNMT1
180kDa
130kDa
100kDa
70kDa
NHE7-70-100 kDa
55kDa
40kDa
β-actin-42 kDa

Fig S10C

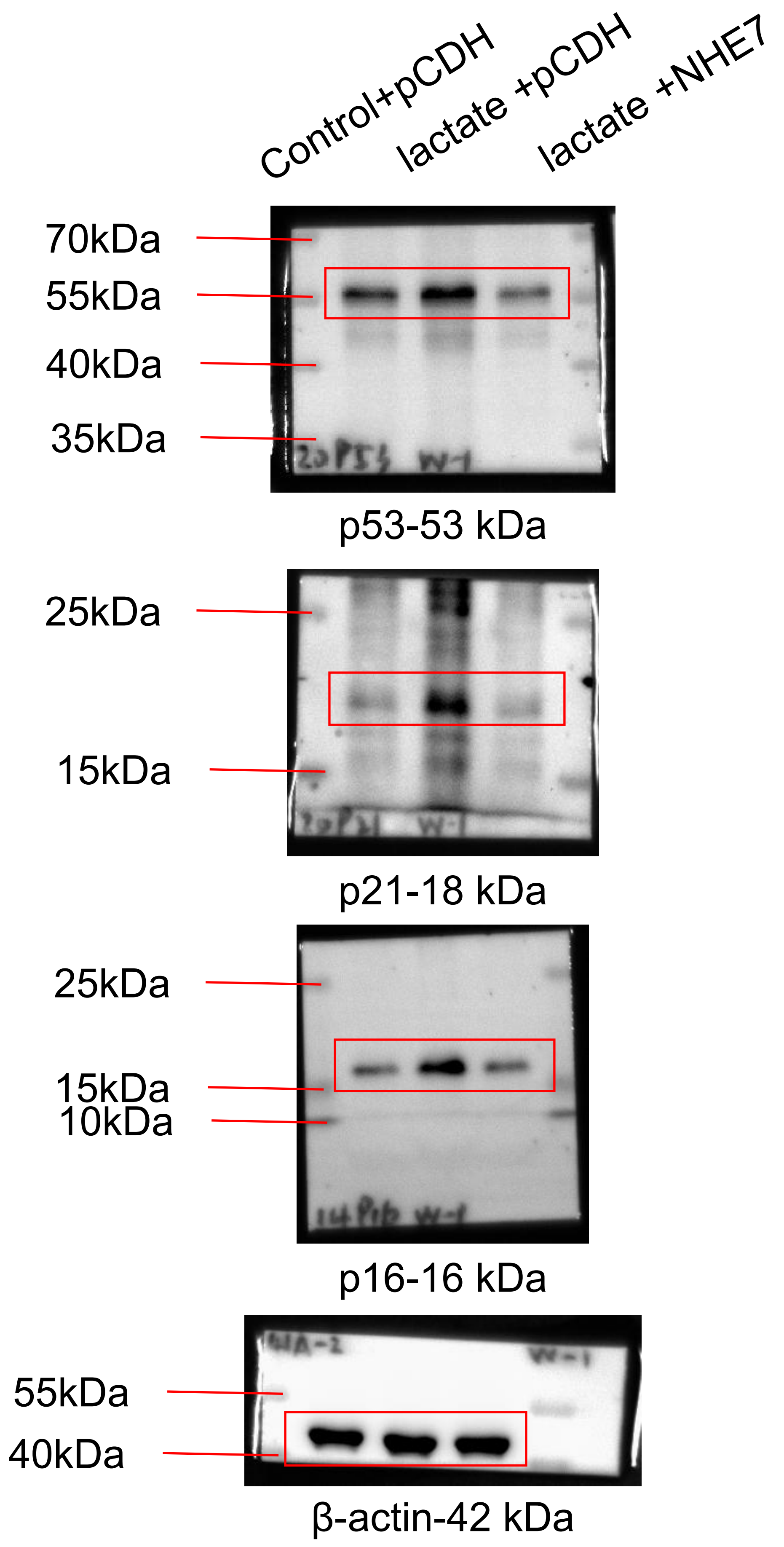

Fig S10D

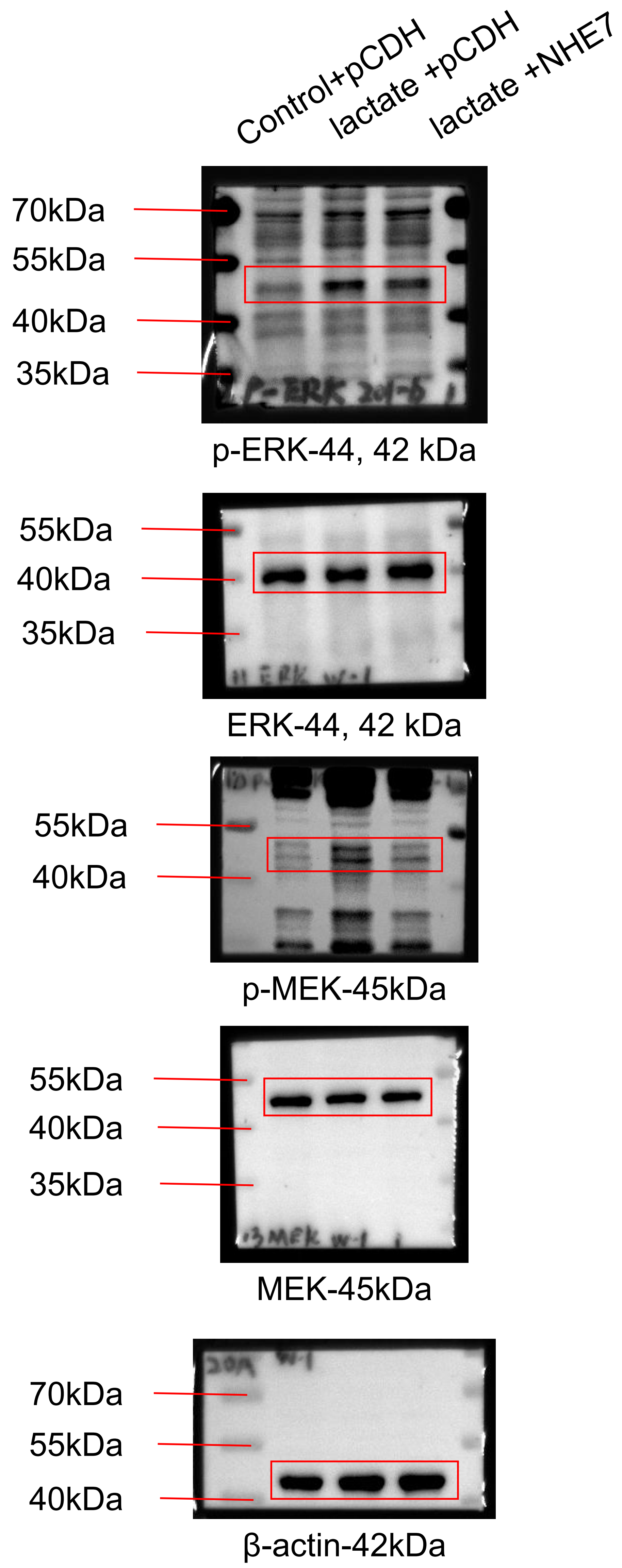

Fig 11A

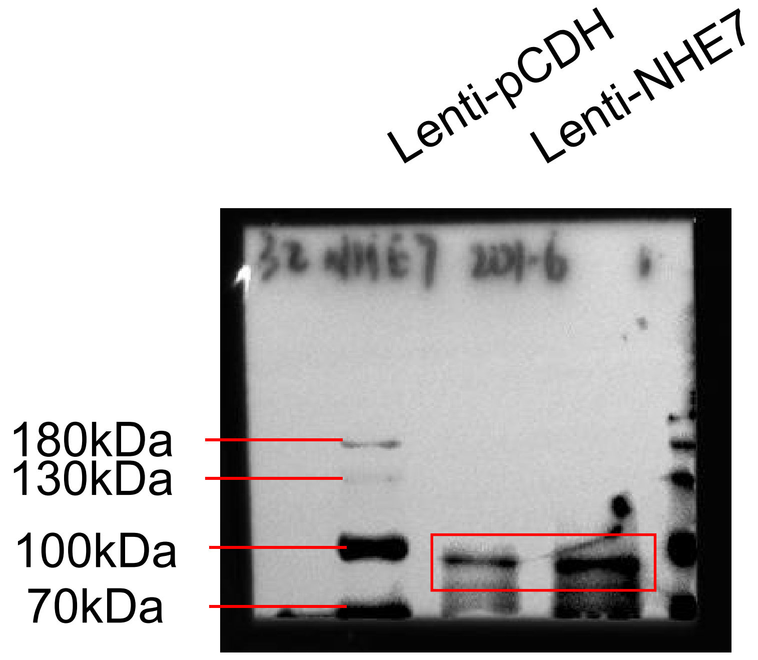

NHE7-70-100 kDa

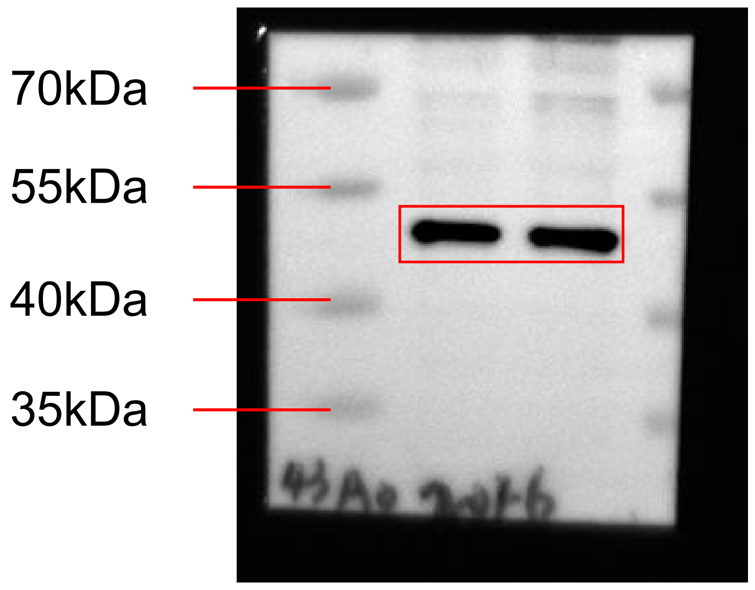

β-actin-42 kDa

Supplementary Material 3. The confirmation of publication and licensing rights for graphical abstract, created with BioRender.com

49 Spadina Ave. Suite 200
Toronto ON M5V 2J1 Canada
www.biorender.com

Confirmation of Publication and Licensing Rights - Open Access

November 26th, 2025

Subscription Type:	*Individual - Academic*
Agreement number:	*BA291JOAAM*
Publisher Name:	*Cell Death & Disease*
Figure Title:	*Graphical Abstract*
Citation to Use:	*Created in BioRender. Lu, L. (2026) https://BioRender.com/t8gqarp*

To whom this may concern,

This document ("Confirmation") hereby confirms that Science Suite Inc. dba BioRender ("BioRender") has granted the following BioRender user: Lee Lu ("User") a BioRender Academic Publication License in accordance with BioRender's Terms of Service and Academic License Terms ("License Terms") to permit such User to do the following on the condition that all requirements in this Confirmation are met:

1) publish their Completed Graphics created in the BioRender Services containing both User Content and BioRender Content (as both are defined in the License Terms) in publications (journals, textbooks, websites, etc.); and
2) sublicense such Completed Graphics under "open access" publication sublicensing models such as CC-BY 4.0 and more restrictive models, so long as the conditions set forth herein are fully met.

Requirements of User:

1) All Completed Graphics to be published in any publication (journals, textbooks, websites, etc.) must be accompanied by the following citation either as a caption, footnote or reference for each figure that includes a Completed Graphic:
   "Created in BioRender. Lu, L. (2026) https://BioRender.com/t8gqarp ".
2) All terms of the License Terms including all Prohibited Uses are fully complied with. E.g. For Academic License Users, no commercial uses (beyond publication in journals, textbooks or websites) are permitted without obtaining or switching to a BioRender Industry Plan.
3) A Reader (defined below) may request that the User allow their figure to be a public template for Readers to view, copy, and modify the figure. It is up to the User to determine what level of access to grant.

Open-Access Journal Readers:

Open-Access journal readers ("Reader") who wish to view and/or re-use a particular Completed Graphic in an Open-Access journal subject to CC-BY sublicensing may do so by clicking on the URL link in the applicable citation for the subject Completed Graphic.

The re-use/modification options below are available after the Reader requests the User to adapt their

figure as a BioRender template and the User has granted such access.

1) View-Only/Free Plan Use: A Reader who wishes to only view the Completed Graphic may do so in the BioRender Services as either a BioRender Free Plan user or simply as a viewer. By becoming a BioRender Free Plan user, the Reader may view, modify and re-use the Completed Graphic as permitted under BioRender's Basic License Terms (e.g. personal use only, no publishing or commercial use permitted).
2) Re-Use/Publish with No Modifications: For any re-use and re-publication of a Completed Graphic with no modification(s) to the Completed Graphic made by the Reader, a Reader may do so by citing the original author using the citation noted above with the Completed Graphic. The Reader must also comply with the underlying License Terms which apply to the Completed Graphic as noted above (e.g. no commercial use for Academic License).
3) Re-Use/Publish with Modifications: For any re-use and re-publication of a Completed Graphic with a modification(s) made by the Reader, the Reader may do so by becoming a BioRender user themselves under either an Academic or Industry Plan, citing the original author using the citation noted above with the Completed Graphic and complying with the applicable License Terms.

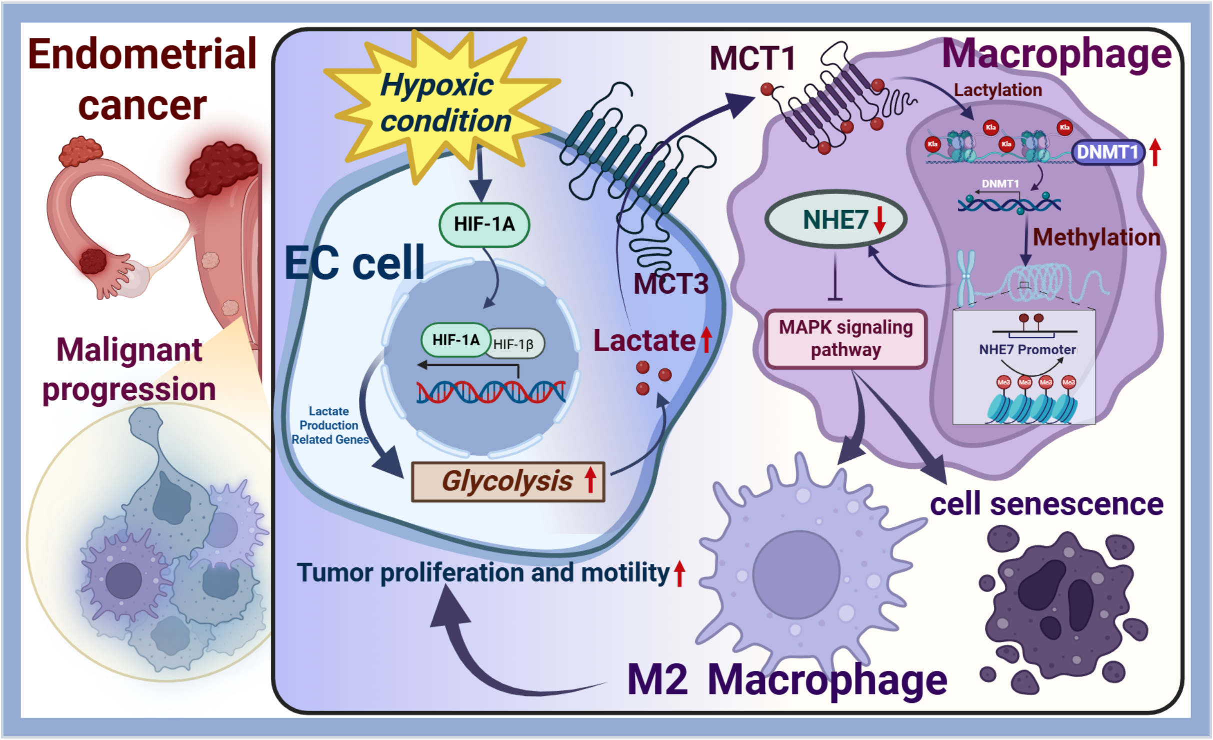

For any questions regarding this document, or other questions about publishing with BioRender, please refer to our BioRender Publication Guide, or contact BioRender Support at support@biorender.com.
